# Quantum Chemical Exploration of Fentanyl and Its Analogs: Conformational Landscapes and Energetics in Solution

**DOI:** 10.1021/acsomega.5c07656

**Published:** 2025-11-27

**Authors:** Kimberlyn A. McKnight, E. Liyah Reed, Caroline S. Glick, Leah A. Juechter, Cristina A. Guevara, Caitlin E. Scott, George C. Shields

**Affiliations:** † Department of Chemistry, 3628 Furman University, 3300 Poinsett Highway, Greenville, South Carolina 29613, United States; ‡ Department of Chemistry and Biochemistry, 14669 California State University, Los Angeles, 5151 State University Drive, Los Angeles, California 90032, United States

## Abstract

Opioids are clinical drugs prescribed to manage moderate and severe pain; however, they have negative side effects such as bradycardia, constipation, and respiratory depression. Their addictive properties have led to a drug epidemic and major health crisis in the U.S. Although experimental and computational studies have explored opioid binding and activation of the μ-opioid receptor (MOR), key questions remain about how these interactions relate to physiological properties. We present a quantum chemical data set of 3081 optimized geometries and relative Gibbs free energies, at physiological temperature, for fentanyl and 33 of its analogs in aqueous solution. We find that these compounds are conformationally flexible, and the *cis* conformation, where the phenyl rings are approximately parallel, is most prevalent among the low-energy isomers. For the low-energy structures of all 34 analogs, we compare the conformations and energetics in solution with experimental binding affinity and potency data, finding little correlation. This suggests that interactions with the receptor, rather than intrinsic conformational preferences alone, play a dominant role in binding and activity. We expect the data set to be useful for future studies of opioid-MOR interactions.

## Graphical abstract

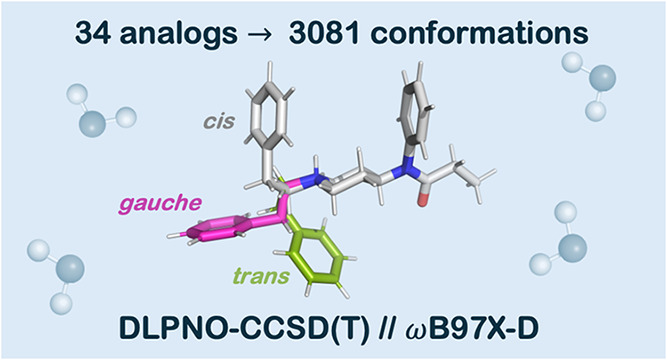

## Introduction

Opioids are commonly prescribed to manage moderate to severe pain following injury or medical procedures. These substances, such as methadone, morphine, and fentanyl, function as opioid agonists, meaning they bind to opioid receptors to elicit a physiological response. Common responses induce analgesia, euphoria, sedation, respiratory depression, bradycardia, nausea, and vomiting.^1^ However, the highly addictive potential of opioids has contributed to a persistent drug epidemic and major public health crisis in the United States. In 2021, fentanyl alone was responsible for 70,000 United States adults’ overdoses. Fentanyl is also the primary agent in the pediatric opioid crisis and has been implicated in 37.5% of pediatric deaths from 1999 to 2021.^2^

All opioid agonists act primarily on the central nervous system by binding to the μ-opioid receptor (MOR). MOR is a G-protein coupled receptor (GPCR), which is characterized by seven α-helices that span the lipid bilayer. Opioid agonist binding induces a conformational change involving the α-helices and subsequent activation of intracellular signaling pathways. Of particular interest is the distinction between ligands that preferentially activate the G_i_-protein pathway, which is associated with analgesia as well as fewer side effects, and those that also activate the β-arrestin pathway, which is more strongly linked to unwanted side effects like respiratory suppression and constipation.^3^ Experimental studies have shown that ligands that recruit β-arrestin (like fentanyl) form key interactions to MOR helices 3, 6, and 7, while G_i_-biased agonists preferentially interact with helix 3 and make fewer contacts with helices 6 and 7. These interactions influence receptor conformation, promoting different movements of the helices that bias signaling toward either G_i_ or β-arrestin pathways.^4^ The association between signaling bias and specific physiological effects has driven efforts to design MOR agonists that have selective bias for the G_i_ pathway to preserve analgesic effects while minimizing negative side effects.^5–7^ However, this strategy has recently been challenged by various studies.^8–13^ For example, although furanylfentanyl is strongly G_i_-biased, it is associated with severe intoxication and fatal overdoses.^8,14^ A deeper understanding of how agonists interact with and activate MOR is essential for the development of safer opioids.

To better understand the activation of MOR, a combination of techniques, including X-ray crystallography, cryo-electron microscopy, and computational modeling, has been employed. For instance, when the high-affinity agonist BU72 is bound to the MOR, it forms water-mediated hydrogen bonds with Y148^3.33^ and K233^5.39^ (superscripts are in Ballesteros-Weinstein numbering format^15^) in the orthosteric binding pocket, in addition to H297^6.52^, which has been observed in the inactive opioid receptors.^16,17^ The endogenous peptide DAMGO also forms a water-mediated hydrogen bond to H297^6.52^.^18^ Computational studies have further revealed key interactions involved in MOR activation. In the presence of BU72, helix 3 shifts toward helix 2 so that the agonist can form contacts with D147^3.32^ and M151^3.36^. Molecular dynamics simulations (MD) show that the movement of D147^3.32^ is coupled with N150^3.35^, which switches from interacting with the sodium ion in the inactive state to I146^3.31^ in the active state. Similarly, W293^6.48^ has different rotamers during activation. All of these residues interact with a conserved triad of amino acids (I^3.40^, P^5.50^, and F^6.44^) that have been proposed to be responsible for propagating an activation signaling cascade from the ligand binding site to the intracellular end of the GPCR in both MOR and the β2 adrenergic receptor.^17^ Investigations of the differences between morphine-MOR and fentanyl-MOR model complexes with inactive (PDB ID: 4DKL) and active (PDB ID: 5C1M) receptor structures observed the conformational orientations of fentanyl were stabilized through specific water-mediated interactions with amino acids.^19^ However, the active MOR structures used in these studies are derived from *Mus musculus*. The conformation of the mouse MOR with bound BU72 and nanobody is different from human MOR (hMOR) with bound fentanyl and G_i_.^20^ These studies convey foundational information, but left unknown the exact mechanisms and conditions fentanyl would induce on the MOR in a human. It is also important to note that these MD studies generally used transferable small-molecule force fields, where ligand parameters are assigned by analogy to existing atom types. This approach differs from QM-based parameter derivation, where new parameters are fitted using QM calculations on the full ligand or smaller fragments. While analogy-based assignment is a common practice, this introduces uncertainties in predicted binding poses and dynamics.

To address the species-related limitation, Xie et al. constructed an hMOR model by modifying a mouse MOR and G_i_ template to match the human sequence. Their study revealed that the preferred binding poses of fentanyl and a few analogs aligned closely with the global minimum on the free energy landscape of the ligands in the absence of the receptor, determined by metadynamics simulations.^21^ In 2022, cryo-electron microscopy was used to experimentally determine the structure of hMOR bound to fentanyl and the G_i_. The fentanyl-hMOR binding mode is characterized by hydrophobic interactions between: phenylethyl and helices 2 and 3; propionyl and I298^6.51^ and V302^6.55^; and the *n*-aniline ring and M153^3.36^, W295^6.48^, I298^6.51^, G327^7.42^, and Y328^7.43^.^4^ Single point mutations of D149^3.32^A, Q126^2.60^K, and Y328^7.43^A in hMOR show significant decreases in G_i_ activation in the presence of fentanyl. According to the experimental structure, fentanyl forms a salt-bridge with D149^3.32^, which in turn forms hydrogen bonds with Q126^2.60^ and Y328^7.43^.^4^ In our previous study, which used hMOR, we found that the experimentally bound ligand is best matched by the sixth-lowest energy fentanyl conformation in solution, according to quantum mechanics. Furthermore, docking calculations indicate that it is possible for other ligand conformations, different from the experimental pose, to bind to the receptor. It was hypothesized that the flexibility of fentanyl may explain why fentanyl induces multiple intracellular protein binding and signaling pathways, resulting in different physiological effects.^20^

Given the rapid emergence of fentanyl analogs and the urgent need to understand their behavior, as well as classify new and existing forms, it is essential to evaluate their structural features and conformational preferences. Outside of receptor-bound structures, X-ray crystallography has been applied to fentanyl and its analogs.^22,23^ These studies provide conformations that are stabilized in the solid state, which can serve as benchmarks for quantum mechanics, though such conformations may differ from those relevant in solution or receptor binding. Quantum mechanical studies of fentanyl and its analogs have led to force field parametrization, the generation of initial ligand conformations for docking, and the interpretation of experimental spectroscopic data. For example, dihedral scans of fentanyl’s dihedral involving the piperidine ring and the aniline group using MP2/6–31G* revealed three energy minima (37°, 87°, and 120°), while the same scan for *N*-(3-fluoro-1-phenethylpiperidin-4-yl)-*N*-phenylpropionamide) showed minima at 36° and 107°.^24^ These QM-derived data were subsequently used to assign torsional parameters in molecular mechanics force fields. Both classical MD^25^ and QM-based MD^26^ simulations indicate that fentanyl remains flexible in water. QM methods have also been used to prepare ligands for docking studies. Initial conformations of 22 fentanyl analogs were generated via Monte Carlo and molecular mechanics, followed by gas phase optimizations with B3LYP/6–31G**.^27^ Furthermore, B3LYP/6–311 ^28^ and PBEh-3c^29^ have helped assign vibrational modes in experimental spectra. Apparent in all relevant literature is the flexibility of fentanyl, and we aim to analyze this flexibility in terms of physiological effects.

Hassanien et al.^30^ reported the pharmacological (binding affinity and potency) and spectral properties of 34 fentanyl analogs, but to our knowledge, no prior study has evaluated the conformational differences of these 34 fentanyl analogs in the context of their potential for binding to hMOR. Figure 1 shows fentanyl and the R groups that are modified to create the 33 derivatives. The modifications, as well as binding affinity and potency data, are presented in Table 1. Binding affinity is represented by *K_i_* —a lower *K_i_* indicates that the drug binds more tightly to the target. Potency is given by EC_50_, which is the effective concentration needed to produce 50% of the maximal response in a functional assay; therefore, a lower EC_50_ indicates a higher potency. Some structure–activity relationships have already been established for this set of analogs. For example, lower affinities and potencies are seen for analogs with very small or very large substitutions of R_1_. Furthermore, sterics and electron density of R_2_ substituents affect the binding affinity and potency.^30^

**Figure 1:**
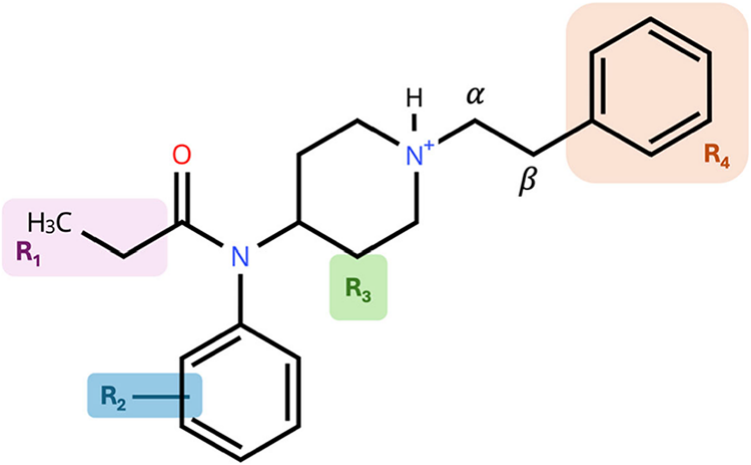
Fentanyl with R groups identified as sites of modification. Adapted from Hassanien et al., ref 30.

**Table 1:** Fentanyl and Its 33 Analogs, Adapted from Tables 1 and 2 of Hassanien et al., ref 30 ^a^

compound #	fentanyl derivative	R_1_	R_2_	binding affinity, *K_i_* (nM)	potency, EC_50_ (nM)
1	fentanyl	ethyl	H	1.6	32
2	cyclopropyl	cyclopropyl	H	2.4	55
3	*p*-methyl cyclopropyl	cyclopropyl	*p*-methyl	7.2	>1000
4	cyclobutyl	cyclobutyl	H	5	160
5	cyclopentyl	cyclopentyl	H	6.6	600
6	furanyl	2-furanyl	H	1.3	9.3
7	tetrahydrofuran	2-tetrahydrofuranyl	H	31	360
8	acetyl	methyl	H	64	>2000
9	*o*-methyl acetyl	methyl	*o*-methyl	43	>1000
10*	α-methyl acetyl	methyl	H	19	>500
11	acryl	ethylene	H	2.1	68
12	*o*-fluoro acryl	ethylene	*o*-fluoro	1.1	14
13	*p*-fluoro acryl	ethylene	*p*-fluoro	4.3	84
14	isobutyryl	isopropyl	H	6.6	137
15	*o*-fluoro isobutyryl	isopropyl	*o*-fluoro	1.3	42
16	*m*-fluoro isobutyryl	isopropyl	*m*-fluoro	4.5	>500
17	*p*-fluoro isobutyryl	isopropyl	*p*-fluoro	24	>1000
18	*p*-chloro isobutyryl	isopropyl	*p*-chloro	82	>2000
19	pivaloyl	*tert*-butyl	H	4.5	531
20	butyryl	propyl	H	3.5	80
21	*o*-fluoro butyryrl	propyl	*o*-fluoro	0.7	60
22	methoxyacetyl	methoxy methylene	H	17	>500
23	*o*-fluoro	ethyl	*o*-fluoro	0.4	15
24	*m*-fluoro	ethyl	*m*-fluoro	10	164
25	*p*-fluoro	ethyl	*p*-fluoro	4.2	79
26	*o*-methyl	ethyl	*o*-methyl	3.4	58
27	*m*-methyl	ethyl	*m*-methyl	5.5	450
28	*p*-methyl	ethyl	*p*-methyl	4.2	>1000
29	*p*-chloro	ethyl	*p*-chloro	45	>1000
30*	*cis*-3-methyl	ethyl	H	0.32	4.2
31*	*trans*-3-methyl	ethyl	H	1.1	25
32*	furanylethyl	ethyl	H	8	350
33*	β-hydroxy	ethyl	H	6.2	138
34*	β-methyl	ethyl	H	14	>500

a Each analog is classified by their different modifications from specific R groups, shown in Figure 1. Binding affinity and potency data are in nM. Asterisks (*) indicate the further modifications are made outside of R_1_ and R_2_. They are (10) α-methyl, (30) *cis*-methyl for R_3_, (31) *trans*-methyl for R_3_, (32) 2-Furan for R_4_, (33) β–OH, 2-Thiophene for R_4_, (34) β-methyl.

In this study, we present quantum-chemically optimized geometries, relative energies, and thermodynamic corrections for 3081 low-energy conformations of fentanyl and 33 analogs in aqueous solution at physiological temperature, 310 K. We report relationships between the analog conformations and known binding affinities and potencies, as well as the impacts of implicit solvent and basis sets augmented with diffuse functions in the quantum chemical energy calculations. These structures can be used to (1) simulate ligand–receptor interactions via docking, MD, or QM/MM methods, (2) guide the design of new, safer fentanyl analogs, (3) improve detection methods, and (4) train machine-learning models to help create or classify emerging analogs.

## Methods

The coordinates of fentanyl and its other derivatives, presented in Hassanien et al.,^30^ were retrieved from PubChem and protonated, in agreement with p*K*_a_ calculations of fentanyl in aqueous solution.^20^ Each fentanyl analog was subjected to a computational funnel protocol, where increasingly time-consuming and accurate calculations were performed at each step. This protocol has been successful in the analysis of atmospheric and pure water clusters, making it useful for describing organic molecules with noncovalent interactions.^31,32^ The funnel method is shown in Figure 2 and described below.

**Figure 2:**
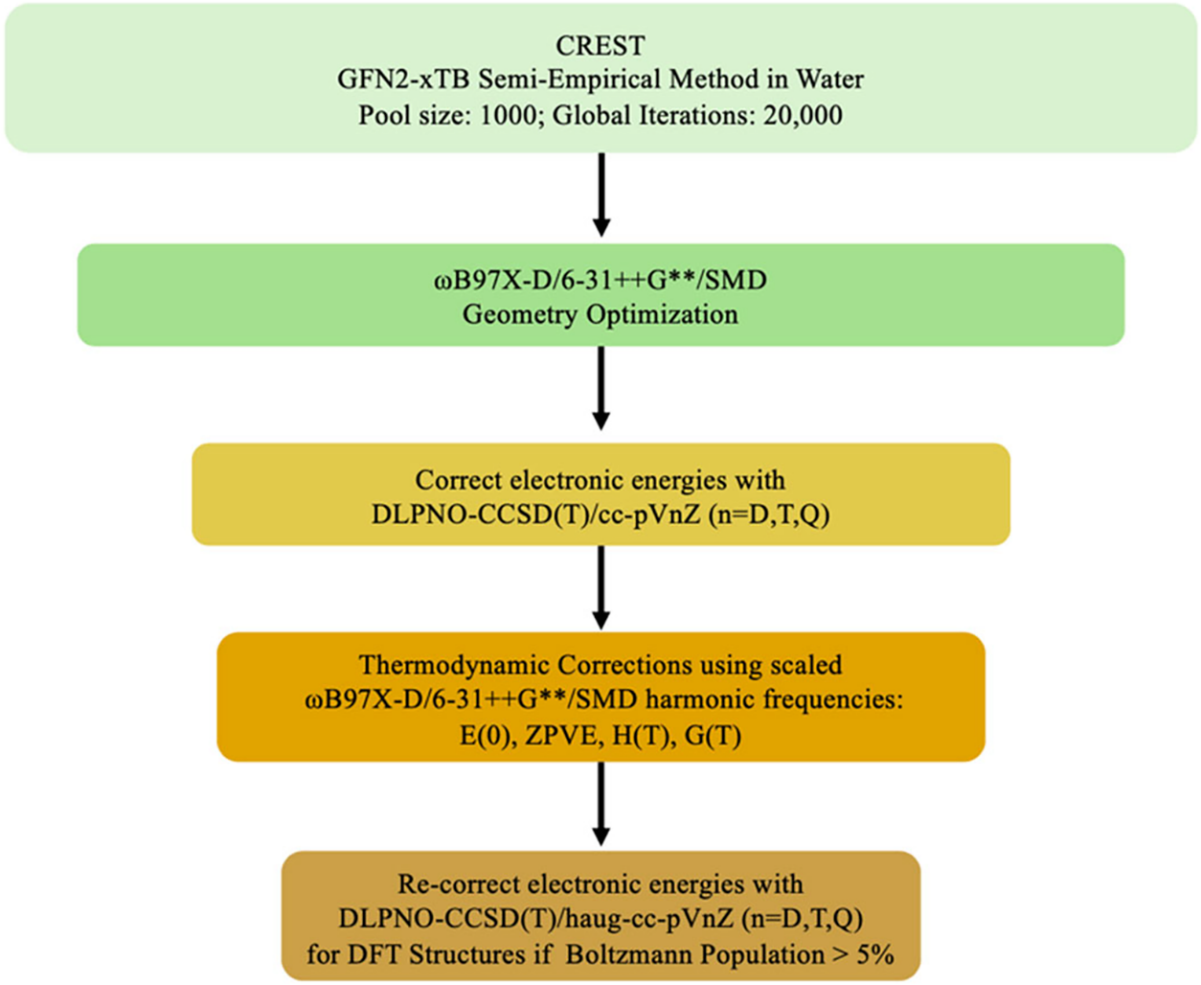
Computational funnel by which a pool of geometries was generated using a generic algorithm (GA), then refined through increasingly accurate density functional (DFT) geometric optimizations, followed by DLPNO–CCSD­(T) energy calculations extrapolated to the complete basis set limit.

### Initial Conformational Sampling and Geometry Optimization

An initial set of conformers are generated for each structure using Conformer-Rotamer-Ensemble Sampling Tool, CREST,^33^ with the GFN2-*x*TB semiempirical method^34,35^ and generalized Born and surface area (GBSA) continuum solvation model^36,37^ to represent water. Duplicate conformers, those with rotational constants within 1% and energies within 0.1 kcal/mol, were deleted, and the unique conformers were optimized^38,39^ with the ωB97X-D^40,41^ density functional and 6–31++G** ^42–46^ basis set using Gaussian 16.^47^ The 6–31++G** basis set contains diffuse and polarization functions on all atoms, which are helpful when describing intermolecular interactions and diffuse electron density. During the optimization, the SMD implicit solvent model^48^ (a solvent model that considers solute electron density) was used to simulate bulk water surrounding the opioid structures via a dielectric field. Implicit solvent models are advantageous due to their low computational expense, as opposed to directly adding explicit water molecules to the system.^49^ SMD was specifically chosen for its ability to capture hydrogen bonding effects.^48,50,51^ The resulting DFT-optimized structures were compared to the lowest energy structure for each analog using root-mean-square-deviations (RMSDs) calculated from ArbAlign.^52^

### Correcting Electronic Energies

Density functional theory (DFT) with dispersion corrections usually produces reliable geometries relative to highly accurate levels of theory. Unfortunately, electronic energies calculated by DFT are less accurate.^32^ Coupled-cluster with single, doubles, and perturbative triple excitations [CCSD­(T)], referred to as the gold-standard method of quantum chemistry due to its accuracy in the complete basis set (CBS) limit,^32,53^ is unusable in many applications due to its scaling: O­(*N* ^7^), where *N* is the number of basis functions.^54^ Domain-based local pair natural orbital CCSD­(T) theory [DLPNO–CCSD­(T)] provides an efficient route to near-CCSD­(T) accuracy by only considering electron pairs with significant contributions to the correlation energy.^55–66^ We computed DLPNO–CCSD­(T)/SMD electronic energies for conformers with ωB97X-D/6–31++G**/SMD electronic energies within 8 kcal/mol of the minimum energy structure for each analog. The Dunning correlation-consistent basis sets^67,68^ of double-, triple-, and quadruple-ζ were used: cc-pVDZ, cc-pVTZ, cc-pVQZ. We also extrapolated the DLPNO–CCSD­(T) energies to the CBS limit using energies from the three consecutive basis sets (DZ, TZ, and QZ) and the 4–5 inverse polynomial scheme, as in eq 1 ^69^

1 $$E_{\mathrm{CBS}\left(\mathrm{DTQ}\right)}=\frac{1}{1320}\left(243E_{\mathrm{DZ}}-2048E_{\mathrm{TZ}}+3125E_{\mathrm{QZ}}\right)$$

Going forward, CBS­(DTQ) represents the extrapolated energies with cc-pVNZ (N = D,T,Q) basis sets. All DLPNO–CCSD­(T) calculations were performed in Orca version 5.0.3 ^70^ and using the SMD solvent model for water.

### Temperature-Specific Calculations

Thermodynamic corrections, *H*°_corr_, *S*°_corr_, and *G*°_corr_, were then accounted for at the human physiological temperature of 310.15 K, using ωB97X-D/6–31++G** harmonic frequencies scaled by 0.971 ^71^ and the thermo.pl script from the National Institute of Science and Technology.^72^ These thermodynamic corrections were computed at 1 bar using the ideal gas approximation. SMD solvation free energies, however, are defined with respect to a 1 M standard state in solution. This introduces a discrepancy in standard states. However, the resulting constant offset (∼2 kcal/mol at 310 K) cancels out when comparing relative free energies of conformers of the same molecule.^73^

We scale the frequencies to account for anharmonicity, which is particularly pronounced in low-frequency modes. These modes contribute significantly to enthalpic and entropic corrections, so errors in their treatment can lead to large errors in Gibbs free energies. This is especially concerning to those who calculate Gibbs free energies of weakly bound clusters.^74,75^ The most common method for calculating anharmonic frequencies is second-order vibrational perturbation theory (VPT2).^76,77^ Like any perturbation theory, this method can suffer from numerical errors due to resonances or near-degenerate states. These errors can be reduced by tight geometry convergence and small step sizes for numerical higher order derivatives. An alternative and widely used method, which we employ here, is scaling harmonic frequencies by a factor specific to the level of theory. This technique has been shown to agree well with experiment. For example, scaled harmonic frequencies reproduce experimental frequencies of the water dimer with a root-mean-square deviation of 24 cm^–1^.^78^ Moreover, comparisons of Δ*G* values computed with VPT2 and scaled harmonic frequencies for H_2_SO_4_(H_2_O) and H_2_SO_4_(H_2_O)_2_ differ by only 0.1 kcal/mol.^79^ For the four lowest energy isomers of each (H_2_SO_4_)_2_ and (H_2_SO_4_)_2_(H_2_O), the average error in Δ*G* is 0.25 kcal/mol, and the maximum difference is 0.4 kcal/mol.^80^ Therefore, we find scaling harmonic frequencies to be an efficient method for incorporating anharmonicity into calculations of Gibbs free energies, even for systems with many low-frequency modes.

Gibbs free energies are reported to hundredths of a kcal/mol throughout the paper. While previous studies show that this protocol is effective in capturing relative energetics of organic molecules and clusters,^31,32^ we do not believe our energies are accurate to 0.01 kcal/mol due to various commonly used approximations such as scaled harmonic frequencies, the use of implicit solvent, and approximations in the calculation of correlation energies.

For each analog, Boltzmann distribution percentages (BP) are computed according to eq 2

2 $$\mathrm{BP}=\frac{e^{-\Delta {G{}^{\circ}}_{i}/\mathrm{RT}}}{\sum_{i}e^{-\Delta {G{}^{\circ}}_{i}/\mathrm{RT}}}$$

where Δ*G*°_*i*_ is the Gibbs free energy of conformer *i* relative to the global minimum, *R* is the gas constant, and *T* is temperature (310 K). All BPs in the paper are computed using Δ*G*_*i*_ computed with CCSD­(T)/CBS­(DTQ)/SMD//ωB97X-D/6–31++G**/SMD model chemistry.

We have also calculated the stabilization correction to the Gibbs free energy for each analog according to eq 3 ^81^

3 $${\Delta G^{{}^{\circ}}}_{\left\{A\right\}}=-\mathrm{RT}ln\left(\sum_{i\in \left\{A\right\}}e^{-\Delta {G{}^{\circ}}_{i}/\mathrm{RT}}\right)$$

where Δ*G*°_*i*_ is the Gibbs free energy of conformer *i* relative to the global minimum, calculated with CCSD­(T)/CBS­(DTQ)/SMD//ωB97X-D/6–31++G**/SMD. This is a correction to the global minimum Gibbs free energy of each analog that accounts for the entropic benefit of multiple conformers, the set denoted by {*A*}.

### Addition of Diffuse Basis Functions

For structures with Boltzmann percentages above 5%, we have also computed relative Gibbs free energies with electronic energies that have been corrected further. For these structures, we compute the electronic energies with DLPNO–CCSD­(T)/haug-cc-pVNZ. These basis sets are similar to the ones previously used (cc-pVNZ), except that these are augmented with diffuse functions^67,68^ on the heavy (non-hydrogen) atoms. The inclusion of diffuse functions improves modeling of noncovalent interactions.^82,83^ We use eq 1 to extrapolate the DLPNO–CCSD­(T)/haug-cc-pVNZ energies to the CBS limit and refer to this extrapolated energy as CBS­(haDTQ). We did not compute Boltzmann percentages using CCSD­(T)/CBS­(haDTQ)/SMD//ωB97X-D/6–31++G**/SMD energies because these values were only obtained for a subset of isomers of each analog. Including them would render the denominator in eq 2 incomplete and unrepresentative, leading to unreliable Boltzmann percentages.

### Molecular Dynamics

To better understand the impact of implicit solvation in our protocol, we have analyzed conformations of analog 1 (fentanyl) and analog 34 (β-methyl fentanyl) from molecular dynamics simulations with explicit solvation. For fentanyl, two MD simulations were initiated from distinct starting structures: the crystallized fentanyl in (1) UGIYEP^84^ and (2) PEPCIT10 ^23^ from the Cambridge Structural Database (CSD).^85^ For analog 34, we began simulations with the lowest ranking structure from our data set (*trans* conformation). Partial charges were determined with the Jaguar program, part of the Schrödinger Suite 2024–1.^86–88^ The ωB97X-D theory was used with the 6–31++G** basis set and the Poisson–Boltzmann finite element method^89^ to model water solvation and determine the Mulliken populations. Using Desmond software,^90,91^ the molecule was then placed in an explicit water box using the TIP4PEw^92^ model with a 15 Å buffer on each side, a salt concentration of 0.15 M NaCl, and an extra chloride ion to neutralize the system. The OPLS4 force field^93^ was used to construct the system except for the charges on the ligand atoms. Before MD was conducted, the system was relaxed using a default protocol. MD was run for 100 ns with a 2 fs time step with the NPT ensemble at a constant pressure of 1.01325 bar and temperature of 310 K. The Nosé-Hoover thermostat^94,95^ was used with a relaxation time of 1.0 ps, and the Martyna-Tobias-Klein barostat^96^ was used with a 2.0 ps relaxation time and an isotropic coupling style. The cutoff radius was 10.0 Å for calculating Coulombic interactions. MD simulations were run with Desmond software on an NVIDIA GeForce RTX 4070 GPU. Frames were saved every 10 ps for a total of 10,002 frames. Clustering was performed with Desmond’s trajectory frame clustering program using every frame. The root-mean square deviation (RMSD) was calculated for the ligand atoms, and a resulting RMSD matrix was used by the affinity propagation clustering method.^97^ For each cluster, a representative trajectory frame was selected as the exemplar (representative structure), defined as the structure most similar to all other members of that cluster according to RMSDs.

## Results and Discussion

### Conformational Isomers

Table 2 presents, for each analog, the number of unique structures retained throughout the computational funnel method. In total, 10,545 unique structures were found in the conformational sampling step, which led to 3992 unique ωB97X-D/6–31++G** geometries. Of those DFT geometries, 3081 had relative ωB97X-D/6–31++G** electronic energies within 8 kcal/mol of each respective analog’s global minimum. For these 3081 conformers, Δ*G*°s were computed, and electronic energies were corrected with DLPNO–CCSD­(T)/cc-pVNZ (N = D,T,Q). For the 149 structures with Boltzmann populations over 5%, the electronic energies were also corrected with DLPNO–CCSD­(T)/haug-cc-pVNZ (N = D,T,Q).

**Table 2:** Number of Unique Structures for Analog 1 (Fentanyl) and 33 Derivatives (Analogs 2–34) Retained throughout the Computational Funnel Method^a^

analog	CREST	ωB97X-D/6–31++G**	DLNPO–CCSD(T)/ cc-pVNZ	DLPNO–CCSD(T)/ haug-cc-pVNZ	Δ*G*°_{A}_, kcal/mol
1	229	82	60	3	–0.34
2	259	89	69	7	–0.97
3	177	50	37	3	–0.57
4	145	61	49	4	–0.58
5	253	100	77	5	–0.81
6	157	70	55	6	–0.71
7	485	206	146	5	–0.98
8	126	40	32	3	–0.17
9	213	52	49	5	–0.52
10	162	65	51	3	–0.36
11	172	71	51	4	–0.33
12	292	136	101	4	–0.52
13	228	83	60	3	–0.66
14	226	83	59	4	–0.52
15	288	85	61	5	–0.83
16	320	127	127	6	–0.81
17	246	79	53	4	–0.62
18	261	71	51	4	–0.71
19	167	52	52	6	–0.76
20	428	170	133	3	–0.32
21	655	284	210	4	–1.27
22	393	168	118	5	–0.39
23	315	129	91	5	–0.80
24	397	173	117	5	–0.90
25	235	87	62	2	–0.31
26	405	143	124	3	–0.65
27	450	133	102	3	–0.66
28	382	95	69	7	–1.03
29	295	93	71	6	–0.95
30	409	114	99	5	–0.65
31	427	130	83	3	–0.67
32	309	137	102	5	–0.81
33	503	296	246	5	–0.94
34	536	238	214	4	–0.42
total	10,545	3992	3081	149	

a “CREST”: the number of unique structures found in our conformational sampling step via CREST. “ωB97X-D/6-31++G**”: the number of unique structures after the CREST structures were optimized with ωB97X-D/6-31++G**. “DLPNO–CCSD­(T)/cc-pVNZ”: the number of DLPNO–CCSD­(T)/cc-pVNZ (N = D,T,Q) calculations. “DLPNO–CCSD­(T)/haug-cc-pVNZ”: the number of DLPNO–CCSD­(T)/ haug-cc-pVNZ (N = D,T,Q) calculations. The final column provides the stabilization correction to the Gibbs free energy, ΔG°_{A}_, in kcal/mol, based on the Boltzmann distribution of conformers.

For each analog, figures of conformers with Boltzmann percentages above 0.5% and their relative Gibbs free energies are available in the Supporting Information. Some structures, like analogs 21, 24 and 31, have over 25 conformers with Boltzmann percentages above 0.5%. Others, like analogs 3, 5, 8 and 10, have fewer than 10. All 547 conformers with BP > 0.5% have a Gibbs free energy within 2.98 kcal/mol of their global minimum structure. The Supporting Information figures show that the relative energies are often within tenths of a kcal/mol of each other.

We have calculated the stabilization correction to the Gibbs free energy, Δ*G*°_{A}_, for each analog according to eq 3. This correction to the global minimum free energy of each analog accounts for the entropic contribution of multiple conformers, represented by the set {*A*}.^81^ A more negative stabilization correction implies that there are more conformers that are thermally populated. We found no correlation between this Δ*G*_{*A*}_ and potency or binding affinity, hinting that flexibility in solution may not be the sole reason for stronger or weaker binding affinities or potencies.

Specific conformations can be categorized into three arrangements based on their phenyl ring position:^21^ *cis*, *trans*, and *gauche*. *Cis* orientation refers to the phenyl rings arranging approximately parallel to each other. *Trans* orientation refers to the phenyl rings arranging ∼180° from each other, and *gauche* refers to the phenyl rings arranging ∼90° from each other. Examples of these configurations are shown in Figure 3.

**Figure 3:**
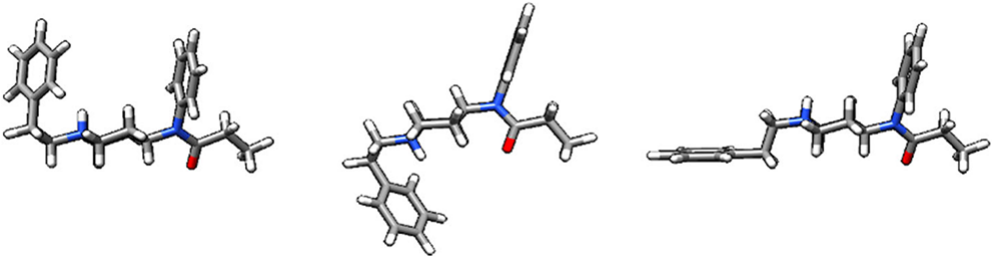
*Cis*, *trans*, and *gauche* conformations of fentanyl. In the *cis* arrangement (left), the phenyl rings are approximately parallel to each other. In the *trans* arrangement (center), the phenyl rings are ∼180° from each other, and in the *gauche* arrangement (right), the phenyl rings ∼90° apart. The nitrogen closest to the phenyl ring on the left-hand side of each conformer is protonated in aqueous solution.

Figure 4 shows the number of *cis*, *trans*, and *gauche* structures with Boltzmann percentages greater than 0.5% for each analog. In total, there are 312 *cis*, 65 *trans*, and 170 *gauche* structures. *Cis* is the dominate conformer type for all but a few analogs including analog 9 (*o*-methyl acetyl fentanyl), analog 31 (*trans*-3-methyl fentanyl), analog 32 (furanylethyl fentanyl), and analog 34 (β-methyl fentanyl). The conformer distributions for these four analogs are (9) 5 *cis*, 2 *trans*, 6 *gauche*; (31) 7 *cis*, 7 *trans*, 12 *gauche*; (32) 7 *cis*, 2 *trans*, 8 *gauche*; and (34) 6 *cis*, 10 *trans*, 9 *gauche*. We attribute this preference to the *cis* conformation for two reasons, intramolecular noncovalent interactions and the hydrophobic effect. The protonated N and the electron-rich phenyl ring just three bonds away from each other are positioned favorably for electrostatic interactions. Also, the mostly parallel phenyl rings are within range of having non-negligible dispersion and electrostatic interactions. The phenyl rings may also adopt the *cis* orientation to minimize contact with water.

**Figure 4:**
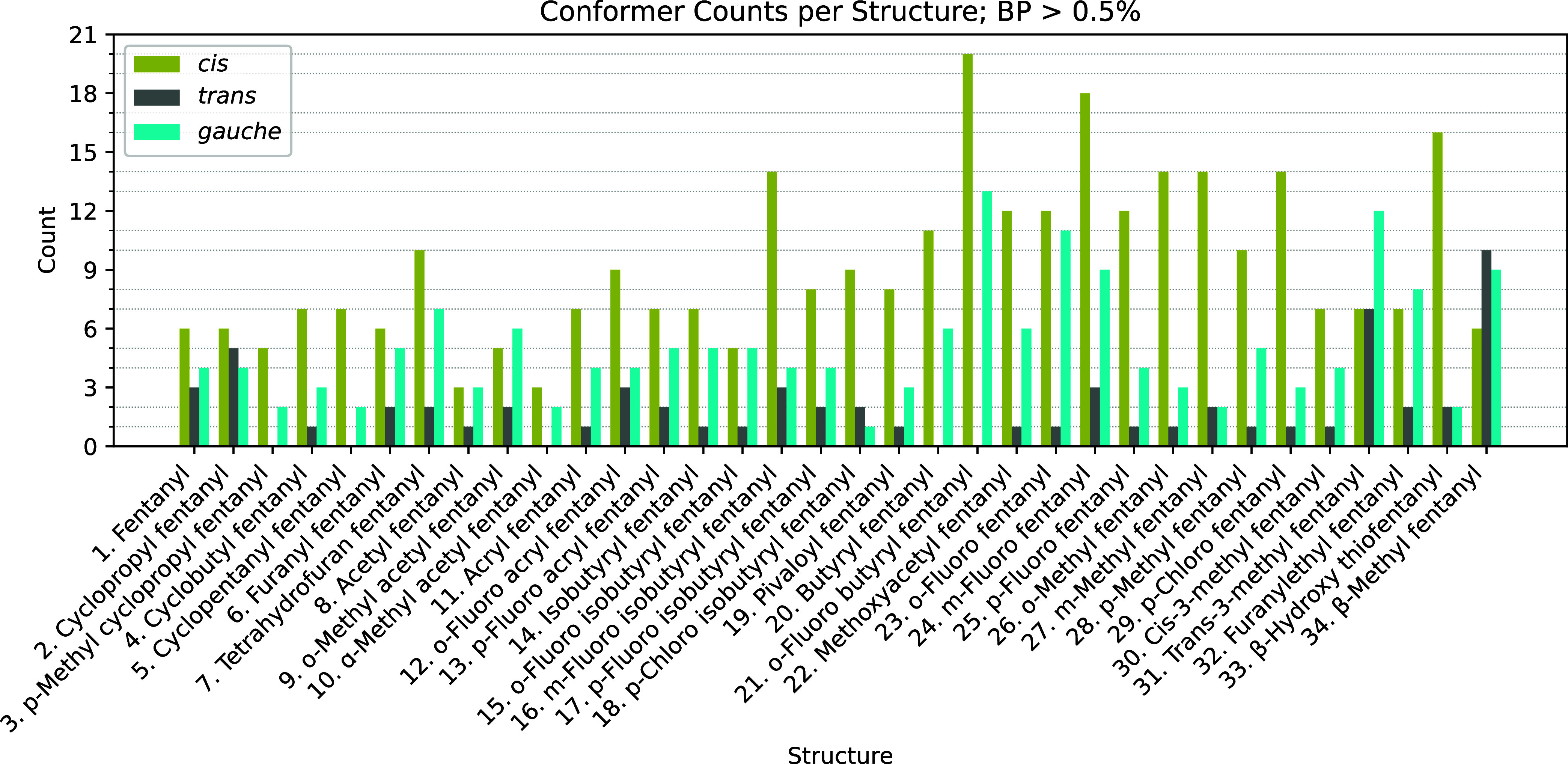
Number of conformers in the *cis* (green), *trans* (gray), and *gauche* (blue) conformation with Boltzmann populations above 0.5%, organized by each analog. Boltzmann populations are computed using Δ*G*°s of DLPNO–CCSD­(T)/CBS­(DTQ)/SMD//ωB97X-D/6–31++G**/SMD model chemistry.

The structures that are not dominated by the *cis* conformation, that is, the structures whose most populous conformation is either *gauche* or *trans*, show no relationship in terms of binding affinity or potency. Furthermore, they each have changes to different R groups of the molecule. Figure 5 shows the four analogs’ non-*cis* lowest-energy structures relative to the low energy fentanyl conformation. Analogs 9 (*o*-methyl acetyl fentanyl) and 31­(*trans*-3-methyl fentanyl) each have a *gauche* conformation as the second lowest-energy structure, with relative Δ*G*°s of 0.61 and 0.22 kcal/mol, respectively. (Six of 13 conformations of analog 9, and 12 of 26 conformations of analog 31, are *gauche*.) In both cases, a methyl group is introduced near the junction of the piperidine ring and the aniline group, and this addition of electrons allows for many low-energy *gauche* conformations. Likewise, eight of 17 structures of analog 32 (furanylethyl fentanyl) are in the *gauche* conformation, and the lowest energy *gauche* structure is 1.04 kcal/mol above the global minimum. In analog 32, the R_4_ phenyl group is replaced with 2-furan, which is more polar than phenyl and is likely stable in the polar solvent. Finally, analog 34 (β-methyl fentanyl) shows 10 of 25 *trans* structures, one of which is the global minimum. In this analog, a methyl group is placed at the β carbon. The lowest-energy structure is able to retain the NH^+^-phenyl interaction while introducing the new methyl group *cis* to the aniline ring.

**Figure 5:**
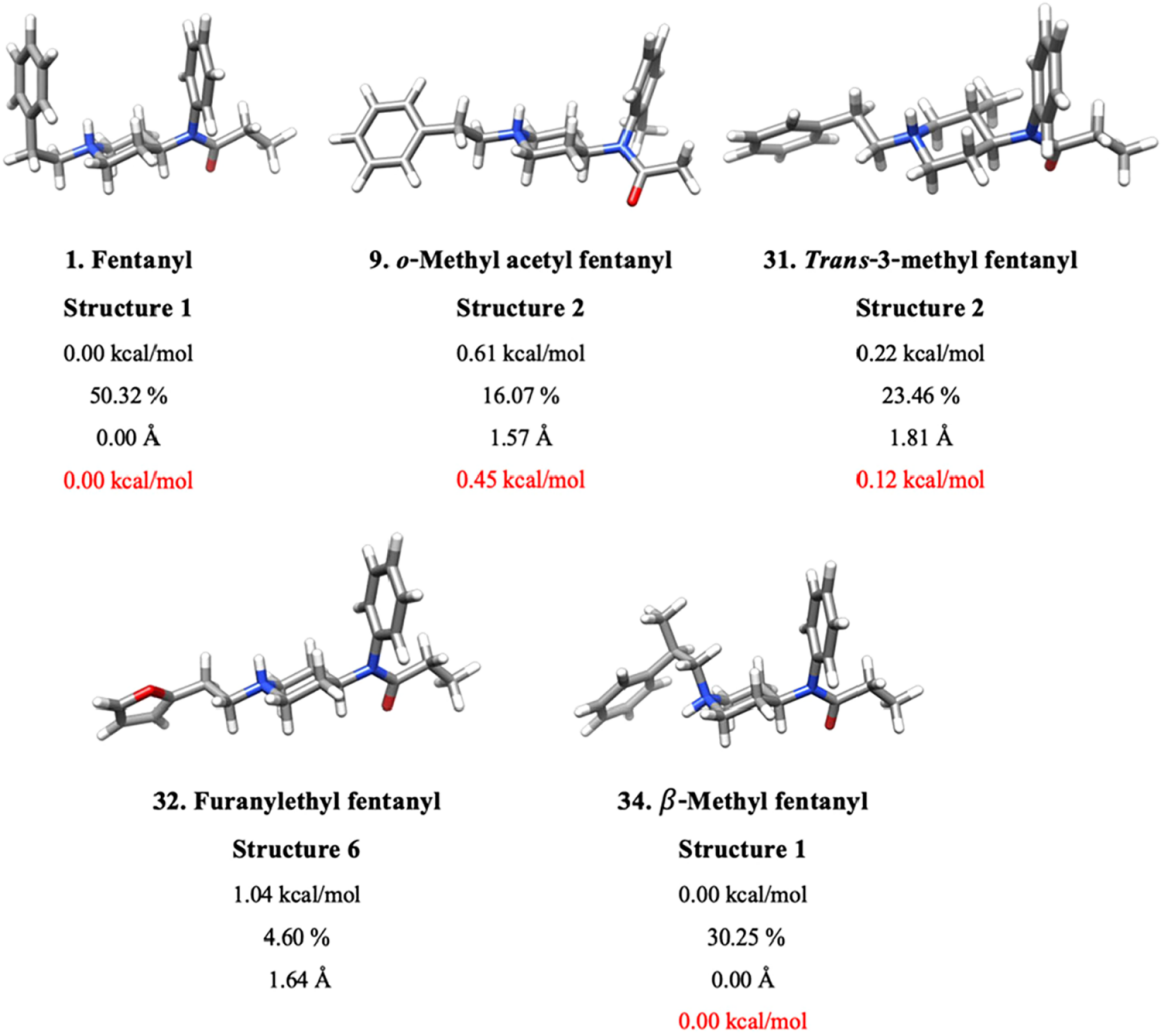
Low energy structures of fentanyl and four of its analogs. The four structures correspond to analogs where *cis* conformations do not dominate for isomers with Boltzmann percentages above 0.5%. Labels beneath each structure give the analog name, the isomer’s rank relative to the analog’s global minimum, the relative Gibbs free energy, Δ*G*°, (kcal/mol) computed with DLPNO–CCSD­(T)/CBS­(DTQ)/SMD//ωB97X-D/6–31++G**/SMD, the Boltzmann percentage, the RMSD (Å) relative to the global minimum structure, and the relative Gibbs free energy, Δ*G*°, (kcal/mol) computed with DLPNO–CCSD­(T)/CBS­(haDTQ)/SMD//ωB97X-D/6–31++G**/SMD if the Boltzmann percentage is above 5%.

The solid-state crystal structure of fentanyl in acetonitrile, and fentanyl citrate salt in toluene, are both available in the CSD^85^ under the codes UGIYEP^84^ and PEPCIT10,^23^ respectively. The experimental conformations do not directly match any of our ωB97X-D/6–31++G**/SMD structures. This is to be expected, as the geometries in the crystal are influenced by crystal packing, counterions, and the solvent polarity. However, upon optimizing the experimental geometries with ωB97X-D/6–31++G**/SMD and a maximum step size of 0.05 Bohr, both return exactly the same structure as the eighth ranked fentanyl structure found through our protocol. UGIYEP’s piperidine nitrogen was protonated, and missing hydrogens were added to PEPCIT10′s terminal methyl group via PyMOL.^98^ The experimental and optimized structures are shown in Figure 6.

**Figure 6:**
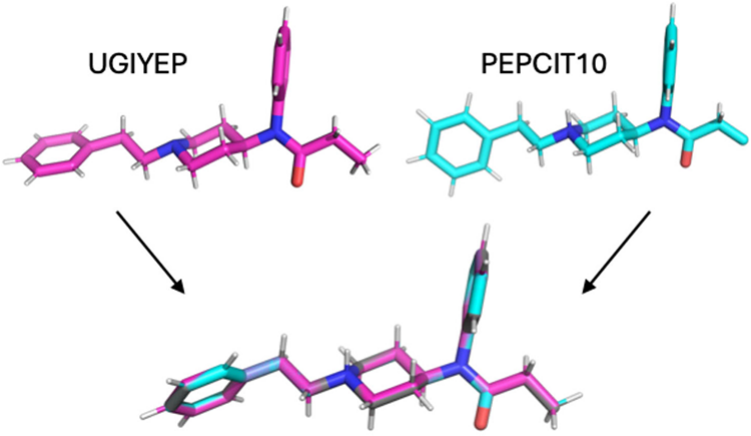
(Top) Fentanyl conformations in the crystal structures UGIYEP^84^ (fentanyl in acetonitrile) and PEPCIT10 ^23^ (fentanyl citrate salt in toluene) of the CSD. (Bottom) ωB97X-D/6–31++G**/SMD optimized UGIYEP (pink) and PEPCIT10 (blue) aligned with fentanyl structure 8 (gray). The nitrogen of the piperidine in UGIYEP was protonated, and missing hydrogens on the terminal methyl were added to PEPCIT10 before optimizations.

### Impact of Solvent Model: Implicit versus Explicit

Each step of our protocol incorporates solvent effects through implicit solvation. Specifically, the configurational sampling in CREST includes the GBSA model, and the quantum calculations use SMD. To evaluate the influence of modeling solvent explicitly rather than implicitly, we performed MD simulations of fentanyl and analog 34 (β-methyl fentanyl) in an explicit water box.

We began simulations for fentanyl starting with two different structures: (1) the crystal structure of fentanyl in acetonitrile, UGIYEP,^84^ which we protonated, (2) the crystal structure of the fentanyl citrate salt in toluene, PEPCIT10.^23^ The crystal structures were obtained from the CSD.^85^ We chose both crystal structures because UGIYEP is in a polar solvent, but not protonated in the crystal, and PEPCIT10 is in a nonpolar solvent, but protonated. We also ran MD of analog 34 (β-methyl fentanyl) using the lowest ranking structure from our data set (*trans* conformation as shown in Figure 5).

Clustering of 10,002 MD frames from each simulation revealed that fentanyl predominately adopts the *cis* conformation, while analog 34 (β-methyl fentanyl) favors the *trans* conformation. These results are consistent with the conformer populations with BP > 0.5% in Figure 4. Representative structures of the most populous *cis*, *gauche*, and *trans* clusters from each simulation of fentanyl (6 structures total) were selected for further analysis and are provided in the Supporting Information. Representative structures from the UGIYEP and PEPCIT10 MD simulations are different, highlighting differences among MD simulations. The RMSDs of representative structures from UGIYEP and PEPCIT10 MD simulations are 1.483 Å (for the two *cis* conformers), 1.871 Å (*trans*), and 0.520 Å (*gauche*).

The representative fentanyl structures were optimized with ωB97X-D/6–31++G**/SMD and a maximum step size of 0.05 Bohr. The optimized *cis* geometries from both the PEPCIT10 and UGIYEP simulations exactly matched our lowest energy conformer (BP = 50.32%) and the *gauche* structures both had RMSDs of 0.375 Å compared to the 13th ranked conformer (BP = 0.54%). The *trans* structure from PEPCIT10 exactly matches a structure with BP = 0.35% (noted gfn2–106 in the Supporting Information), and the *trans* structure from UGIYEP exactly matches structure gfn2–112 (BP = 0.49%). Importantly, none of the representative structures from the explicit solvent MD runs were lower in energy than structures already identified by our sampling protocol, demonstrating that our implicit-solvent approach is sufficient to locate relevant low-energy, solution-phase conformations.

Explicit waters may also influence optimized geometries. To evaluate this effect, we have also reoptimized the representative structures from the UGIYEP and PEPCIT10 MD simulations with ωB97X-D/6–31++G**/SMD while keeping one explicit water (hydrogen bonded to the protonated nitrogen) or two waters (also retaining the water hydrogen bonded to the carbonyl group) from the MD snapshots. Shown in Table 3, the structural response to explicit waters varies depending on the MD starting structure. RMSDs between structures optimized with 2 explicit waters versus 0 or 1 explicit water are often ∼0.3 Å or less, though in some cases the difference reaches 1 Å. These deviations arise from explicit water interactions, which can rotate the terminal phenyl group or aniline group to maximize contact between the π-cloud of the ring and the water’s hydrogen.^99^ This effect for the PEPCIT10 representative structures is shown in Figure 7, and the structures from the UGIYEP simulation are provided in the Supporting Information. PEPCIT10′s *trans* structure has a relatively large change (1.085 Å RMSD) when optimized with 2 explicit waters rather than none, and the different geometries are shown in the top right quadrant of Figure 7. Replacing the second water with an alternative hydrogen-bonded to the carbonyl (bottom left, *trans* B, in Figure 7) reduced the deviation to 0.153 Å. These results illustrate a practical difficulty of explicit solvation: optimized structures, and thus energies, are sensitive to the number and placement of waters, making it unclear how to select representative water configurations.

**Figure 7:**
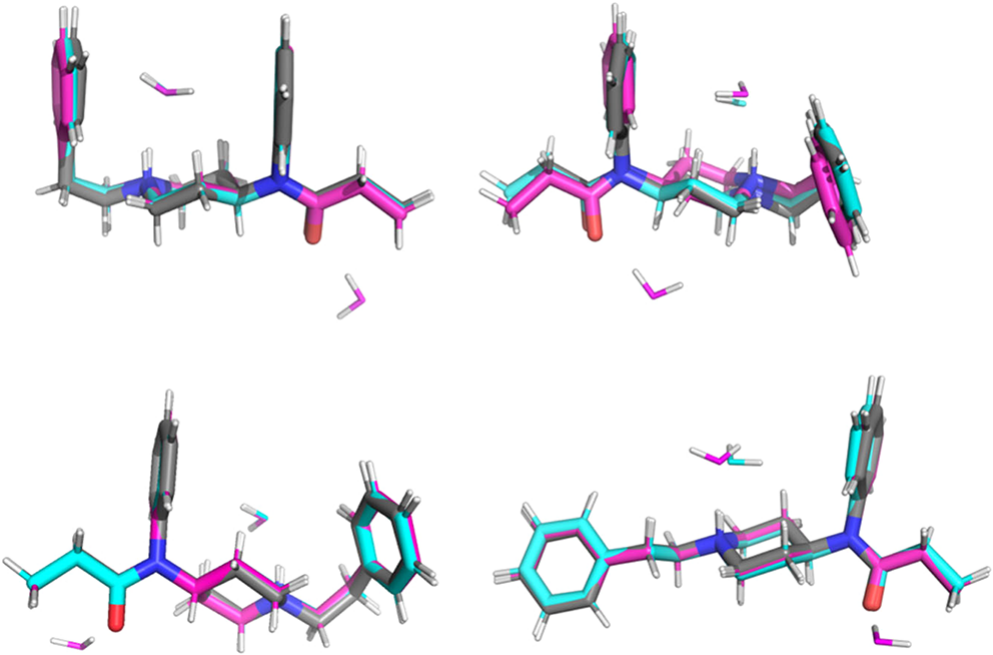
Overlay of representative structures from the PEPCIT10 MD simulation optimized with ωB97X-3/6–31++G**/SMD and 0 (gray), 1 (blue), and 2 (pink) explicit waters. Top left: *cis*; top right: *trans* A; bottom left: *trans* B; bottom right: *gauche*. *Trans* A and *trans* B differ by the selected water retained from the MD simulation hydrogen bonded to the carbonyl carbon.

**Table 3:** RMSDs (Å) between Representative Fentanyl Geometries Optimized with ωB97X-D/6-31++G**/SMD and 0, 1, or 2 Explicit Waters^a^

	UGIYEP			PEPCIT10		
	0:1	0:2	1:2	0:1	0:2	1:2
*cis*	1.175	0.182	1.074	0.196	0.222	0.041
*trans* A	0.365	0.306	0.123	0.150	1.085	1.011
*trans* B	-	-	-	-	0.153	0.047
*gauche*	0.902	0.217	0.840	0.167	0.067	0.127

a For example, 0:1 represents RMSDs between fentanyl optimized with 0 explicit waters and 1 explicit water. The headings “UGIYEP” and “PEPCIT10” refer to the crystal structure that served as the starting structure for the MD simulation. For PEPCIT10, *trans* A and *trans* B refer to different configurations of the second water, both of which are still hydrogen bonded to the carbonyl group.

Finally, we assess the effect of explicit waters on the relative energies. Table 4 reports ωB97X-D/6–31++G** and DLPNO–CCSD­(T)/CBS­(DTQ) relative energies for the representative structures optimized with 0, 1, or 2 explicit waters and SMD. Explicit waters increase the energy gaps between conformers. The relative energies can change by even 1 kcal/mol or more when two waters are included rather than one. These results highlight that convergence with respect to the number of explicit waters is a critical consideration in explicitly solvated studies.

**Table 4:** Relative Energies of the MD Representative Structures Optimized with ωB97X-D/6-31++G** SMD and 0, 1, or 2 Explicit Waters^a^

structure	ωB97X-D/SMD	DLPNO/SMD	ωB97X-D/1 water	DLPNO/1 water	ωB97X-D/2 waters	DLPNO/2 waters
UGIYEP						
*cis*	0.00	0.00	0.00	0.00	0.00	0.00
*trans*	1.68	1.79	2.82	3.21	4.22	3.83
*gauche*	1.86	1.59	3.23	3.29	4.29	3.96
PEPCIT10						
*cis*	0.00	0.00	0.00	0.00	0.00	0.00
*trans* A	1.64	2.00	3.13	3.69	1.62	2.92
*trans* B	1.64	2.00	3.13	3.69	2.71	3.69
*gauche*	1.86	1.59	3.25	3.21	3.46	3.35

a Relative energies are computed with either ωB97X-D/6-31++G**/SMD or DLPNO–CCSD­(T)/CBS­(DTQ)/SMD. Representative structures are taken from MD simulations of UGIYEP or PEPCIT10 in a box of explicit waters.

Overall, we have shown than our implicit-solvent protocol reliably identifies key solution-phase minima, yields optimized geometries that differ normally by only small (≤0.3 Å) RMSDs from explicit-solvent structures, and produces relative energies that, while somewhat sensitive to the number of explicit waters, remain chemically reasonable. Given these results, and the variability of explicit solvent models, our approach represents a practical and robust strategy for a systematic study of this scale.

### Impact of Basis Sets on Relative Energies

As discussed, the noncovalent intramolecular interactions in fentanyl and its analogs play a key role in stabilizing specific conformations in solution. Basis sets with diffuse functions often better describe noncovalent interactions.^82,83^ Therefore, we have recomputed electronic energies of our data set with DLPNO–CCSD­(T)/haug-cc-pVNZ (N = D,T,Q)/SMD for structures with Boltzmann populations >5%.

Table 5 reports the mean and maximum absolute deviations in relative energies when using haug-cc-pVNZ basis sets instead of the cc-pVNZ basis sets. At the double-ζ level, the impact can be as large as 1.189 kcal/mol as in analog 20 (butyryl fentanyl). Both the mean and maximum deviations decrease as the ζ-level increases, which is expected since the influence of augmented diffuse functions typically diminish as the basis set size increases. By QZ, the mean absolute error falls to 0.069 kcal/mol, and the maximum deviation is 0.425 kcal/mol. The CBS­(DTQ) energies yield mean and maximum deviations larger than those of TZ, a consequence of the larger differences at the double-ζ level.

**Table 5:** Mean and Maximum Absolute Deviations (kcal/mol) for Relative Gibbs Free Energies, Δ*G*°, Computed with DLPNO–CCSD­(T)/cc-pVNZ/SMD//ωB97X-D/6-31++G**/SMD Relative to DLPNO–CCSD­(T)/haug-cc-pVNZ/SMD//ωB97X-D/6-31++G**/SMD, where N = D, T, Q^a^

	DZ	TZ	QZ	CBS(DTQ)
mean	0.322	0.108	0.069	0.137
max	1.189	0.476	0.425	0.767

a CBS­(DTQ) refers to energies extrapolated to the complete basis set limit. Conformers considered have Boltzmann populations above 5%.

Although the energy differences between haug-cc-pVNZ and cc-pVNZ can be small, the relative energies of these flexible conformers are also small, making such differences possibly meaningful. Augmentation alters the rank ordering of conformers in 23 analogs (DZ), 17 analogs (TZ), 14 analogs (QZ), and 19 analogs (CBS­(DTQ)), depending on the ζ-level. In addition, switching to augmented basis sets from nonaugmented changes the identity of the global minimum structure for seven analogs (DZ), four analogs (TZ and QZ), and five analogs (CBS­(DTQ)). In four out of five cases where the global minimum structure changes when moving to CBS­(haDTQ) from CBS­(DTQ), the conformation of the new minimum remains *cis*. Those four analogs are 14 (isobutyryl fentanyl), 16 (*m*-fluoro isobutyryl fentanyl), 18 (*p*-chloro isobutyryl fentanyl), and 27 (*m*-methyl fentanyl). In the one case of analog 13 (*p*-fluoro acryl fentanyl), the global minimum changes from *cis* to *trans*. These results underscore the importance of thorough conformational sampling to identify multiple low-lying minima, as well as the value of using accurate quantum chemical methods for reliable energy predictions.

We have discussed above that increasing the ζ-level of the basis set has proven to be beneficial. For example, relative energies computed with the QZ basis set has the lowest deviation relative to its augmented counterparts. For analog 31, we computed energies with haug-cc-pV5Z and present relative energies of seven conformers in Table 6 as the ζ-level increases. Increasing the basis set from haug-cc-pVTZ to haug-cc-pVQZ changes the relative energies by up to 0.285 kcal/mol. Increasing the basis set to haug-cc-pV5Z improves the haug-cc-pVQZ relative energies by a maximum of 0.068 kcal/mol. While there are some conformational relative energies that differ by this amount or less relative to other conformational energies, those cases are very few. The haug-cc-pVQZ energies are essentially converged, and these very small differences in relative energies when moving from haQZ to ha5Z do not require the use of haug-cc-pV5Z for the calculation of relative energies of fentanyl analog conformations, especially considering its computational cost.

**Table 6:** Relative Binding Free Energies, Δ*G*°, (kcal/mol) Computed with DLPNO–CCSD­(T)/haug-cc-pVNZ/SMD//ωB97X-D/6-31++G**/SMD, Where N = D, T, Q, 5, at 310.15 K for Conformations of Analog 31^a^

	Δ*G* (310.15 K)					ΔΔ*G* (310.15 K)	
structure	haDZ	haTZ	haQZ	ha5Z	CBS(haDTQ)	haQZ-haTZ	ha5Z-haQZ
265	0.000	0.000	0.000	0.000	0.000	0.000	0.000
151	1.920	2.207	2.330	2.321	2.445	0.123	–0.009
161	1.564	1.870	2.067	2.101	2.280	0.197	0.033
128	1.826	2.078	2.363	2.431	2.707	0.285	0.068
207	2.255	2.320	2.408	2.449	2.518	0.089	0.041
261	1.809	1.658	1.617	1.611	1.588	–0.041	–0.006
250	2.360	2.397	2.429	2.448	2.464	0.031	0.020

a haNZ is shorthand for haug-cc-pVNZ. Differences in relative binding free energies, ΔΔ*G* (kcal/mol), at 310.15 K are presented in kcal/mol, and quantify how much the relative energies change when increasing the basis set from haug-cc-pVTZ to haug-cc-pVQZ (haQZ-haTZ) and from haug-cc-pVQZ to haug-cc-pV5Z (ha5Z-haQZ).

### Performance of Semiempirical and DFT Methods

Our computational funnel protocol (Figure 2) begins with conformational sampling with GFN2-xTB, then refines structures with ωB97X-D/6–31++G** and energies with DLPNO–CCSD­(T). With this data, we can assess the accuracy of GFN2-xTB for describing the geometry and relative energies of fentanyl and its analogs.

RMSDs of the semiempirical and DFT geometries were computed for structures of analogs 1–5 with BP > 5% (66 structures). Average RMSDs range from 0.481 Å (analog 1) and 0.602 Å (analog 2), with an overall average of 0.545 Å. The largest deviation, 1.100 Å, occurred for structure 15 of analog 2.

Compared to DLPNO–CCSD­(T)/CBS­(haDTQ) relative energies, GFN2-xTB relative energies have a mean absolute error of 1.16 kcal/mol for all structures with BP > 5% (all analogs) and maximum error of 4.50 kcal/mol. On the other hand, the relative energies calculated via DFT had an average error of 0.21 kcal/mol relative to DLPNO–CCSD­(T)/CBS­(haDTQ). The maximum error was 1.02 kcal/mol, encouraging the use of methods beyond DFT for calculation of accurate relative electronic energies.

We have previously shown that our protocol independently locates local minima structures of fentanyl’s experimental crystal structures UGIYEP^84^ and PEPCIT10 ^23^ (Figure 6) and the representative *cis*, *trans*, and *gauche* structures from molecular dynamics in explicit solvent. Therefore, GFN2-xTB is computationally efficient for sampling the potential energy surface of fentanyl and its analogs, but the large errors in energies and geometries highlight the necessity of higher-level methods for refinement.

### Analysis of the Lowest Energy Structures

Figure 8 shows, for each analog, the relative energies of the lowest energy structures. Structures presented here have a Boltzmann population, computed with DLPNO–CCSD­(T)/CBS­(DTQ)/SMD//ωB97X-D/6–31++G**/SMD, above 5%. The actual relative energies used in the figure are those computed with DLPNO–CCSD­(T)/CBS­(haDTQ)/SMD// ωB97X-D/6–31++G**/SMD. (The Boltzmann percentages should not be calculated with the DLPNO–CCSD­(T)/CBS­(haDTQ)/SMD// ωB97X-D/6–31++G**/SMD energies because we only computed these for 149 isomers; see Table 2). Each marker represents the conformation of the analog: *cis*, *trans*, or *gauche*. Immediately noticeable is the vast number of *cis* conformations: 110. There are 29 *gauche* conformations, and 10 *trans*. *Trans* structures are most prevalent for analog 2 (cyclopropyl fentanyl), where R_1_ is a cyclopropyl group, and 34 (β-methyl fentanyl), which includes a methyl group on the β carbon.

**Figure 8:**
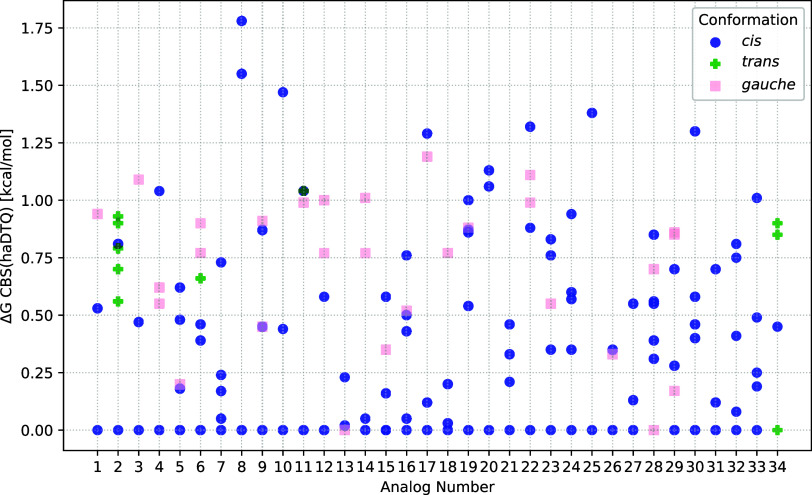
Relative Gibbs free energies, Δ*G*, (kcal/mol) of isomers with Boltzmann populations above 5%. Gibbs free energies are computed with DLPNO–CCSD­(T)/CBS­(haDTQ)/SMD//ωB97X-D/6–31++G**/SMD model chemistry. Isomers are marked by their conformation: *cis* (blue circle), *trans* (green cross), and *gauche* (pink square).

For almost all analogs, the minimum energy structure holds the *cis* conformation. For analogs 13 (*p*-fluoro acryl fentanyl) and 28 (*p*-methyl fentanyl), the minimum energy structure is *gauche*. These structures each have a para substitution for R_2_. For analog 34, the minimum energy structure is *trans*, which has been explained earlier.

There are no obvious relationships between the data presented in Figure 8 and the binding affinity or potency data. We do find it interesting that analog 8 (acetyl fentanyl), which has comparatively weak binding affinity (64 nM) and very weak potency (>2000 nM), has only three conformations with BP > 5%. Its low-energy structures are more than 1.5 kcal/mol above the global minimum, which is higher than all other relative energies in Figure 8. When considering its conformations with BP > 0.5%, the stabilization Gibbs free energy is the lowest of all analogs: −0.17 kcal/mol. Together, these reveal the relative rigidity of acetyl fentanyl, which may explain the poor binding and potency. On the other hand, analog 18 (*p*-chloro isobutyryl fentanyl) has similarly poor binding and potency, yet it does have a few low energy structures and a stabilization Gibbs free energy of −0.71 kcal/mol, which is consistent for this data set. Joining analog 8 (acetyl fentanyl), neither analog 20 (butyryl fentanyl) nor 25 (*o*-fluoro fentanyl) have any low-energy structures within 1 kcal/mol of the minimum structure. These each have slightly higher (worse) binding affinities and potencies than fentanyl, but there exist many analogs within this data set with even worse binding affinities and potencies.

Analog 1 (fentanyl) shows two low-energy *cis* conformations and one *gauche* conformation in Figure 8. The experimental structure of fentanyl bound to hMOR is in the *gauche* conformation.^4^ In solution, four out of the 13 lowest energy structures (those with BP > 0.5%) are *gauche*. The lowest energy *gauche* structure lies 0.94 kcal/mol above the global minimum (using CCSD­(T)/CBS­(haDTQ)/SMD//ωB97X-D/6–31++G**/SMD energies) and has a BP of 6.24%. The other three *gauche* conformations have relative free energies within 2.8 kcal/mol of the global minimum, according to CCSD­(T)/CBS­(DTQ)/SMD//ωB97X-D/6–31++G**/SMD calculations.

It is not clear the conformation that fentanyl takes as it enters the binding pocket. A low energy *cis* conformation could change to *gauche* in solution, then enter the pocket. Alternatively, the slightly higher in energy gauche conformation could enter the pocket. When one conformer binds to MOR, the law of mass action implies that the bound conformer will be repopulated as equilibrium is re-established. There are many low energy structures, so it is plausible that fentanyl’s flexibility makes this repopulation energetically favorable. Future work should evaluate the activation barrier needed for changes between the different low energy conformations. Furthermore, biased molecular dynamics would give insight into the conformational changes of fentanyl occurring between solution and the binding pocket.

Even though the configuration of fentanyl in experiment is *gauche*, for this data set, the abundance of *gauche* conformers does not directly correlate with the binding affinity or potency. Analog 33 has a relatively low total BP from *gauche* conformations: 1.32%. Its binding affinity is 6.2 nM, and its EC_50_ is 138 nM. These are not unusually high for the data set. Analog 27 is another similar case. Its BP of gauche conformations is 3.19%, and its binding affinity is 5.5 nM.

Figure 8 reinforces that fentanyl and its analogs have many low-lying minima in solution at physiological temperatures. Most analogs have isomers within 0.5 kcal/mol of the minimum-energy isomer, and all but three have at least one isomer (but normally many) within 1 kcal/mol. There are no overall trends between these conformations in solution and the pharmacological properties, motivating further investigation of each analog’s interactions with MOR.

## Conclusions

We have presented 3081 optimized solution-phase geometries for fentanyl and 33 of its analogs using the ωB97X-D/6–31++G** level of theory. In addition, we have computed their relative Gibbs free energies with a composite method that combines DLPNO–CCSD­(T)/cc-pVNZ (N = D, T, Q) and ωB97X-D/6–31++G**. For the 149 structures with Boltzmann percentages above 5%, we further refined the electronic energies with DLPNO–CCSD­(T) calculations using diffuse-augmented basis sets (haug-cc-pVNZ, N = D,T,Q).

Our data reveal that fentanyl and its analogs are highly flexible molecules; some analogs have more than 25 isomers with Boltzmann populations above 0.5%. Many of these conformers adopt the *cis* conformation, which we attribute to favorable intramolecular contacts and compactness in solution. Through MD simulations and comparisons to experimental crystal structures, we confirm that our protocol locates relevant solution-phase minima and note the importance of refining energies and geometries with methods beyond those at the semiempirical level.

The inclusion of diffuse functions on heavy atoms was shown to impact the relative energies across all ζ-levels. At the triple-ζ level, the mean absolute error was 0.102 kcal/mol and the max absolute error was 0.476 kcal/mol. At the QZ level, the mean absolute error was 0.068 kcal/mol and the max absolute error was 0.425 kcal/mol. These energy differences are sufficient to alter the rank ordering of isomers in 17 and 14 analogs at TZ and QZ levels, respectively. Hence, neglecting diffuse functions in this case can introduce non-negligible and nonsystematic errors.

While using augmented basis sets, we find that the global minimum conformer for 31 out of 34 analogs adopts the *cis* conformation. Many analogs also exhibit low-energy *gauche* or, less frequently, *trans* isomers. We observe no consistent correlation between flexibility or conformer-type and experimental binding affinity or potency. This suggests that receptor interactions, not just intrinsic conformational preferences, govern binding and activity.

Overall, the structures presented here provide a high-quality data set for further computational and experimental investigation. These conformers serve as promising starting points for future studies of the interactions between fentanyl analogs and the hMOR, hopefully leading to the design of safer opioids. In addition, the geometries can be used to compute spectral features, like those of nuclear magnetic resonance (NMR) or infrared (IR) spectra,^100^ supporting the development of more effective detection methods.^30^

## Supplementary Material









## Notes

XYZ files for all 3081 unique ωB97X-D/6–31++G** structures organized by analog and named according to column A in the spreadsheets (ZIP)
Fentanyl Structures X.xlsx are provided that contain all electronic and Gibbs free energies used in our work, along with Boltzmann percentages and stabilization free energies; analysis of GFN2-xTB relative energies and geometries (GFN2-xTB Analysis.xlsx) (ZIP)
Low-energy structures for each analog. Beneath each structure, labels provide the isomer’s rank relative to the analog’s global minimum, the relative Gibbs free energy computed with DLPNO–CCSD­(T)/CBS­(DTQ)/SMD//ωB97X-D/6–31++G**/SMD, the Boltzmann percentage, the RMSD relative to the global minimum structure, and the relative Gibbs free energy computed with DLPNO–CCSD­(T)/CBS­(haDTQ)/SMD//ωB97X-D/6–31++G**/SMD if the Boltzmann percentage is above 5% (PDF)
Initial and representative structures from the molecular dynamics simulations (ZIP)
