# Supplementary material for: Quantum Chemical Exploration of Fentanyl and Its Analogs: Conformational Landscapes and Energetics in Solution
Source: ACS Omega. 2025 Nov 27;10(48):58900–14. doi: 10.1021/acsomega.5c07656 (PMC12771126; doi:10.1021/acsomega.5c07656)
Supplement: Supplementary file 3 [file ao5c07656_si_003.pdf]

Supporting Information for

Quantum Chemical Exploration of Fentanyl and Its Analogs: Conformational Landscapes and Energetics in Solution

Kimberlyn A. McKnight,[#a] E. Liyah Reed,[#a] Caroline S. Glick,[*a] Leah A. Juechter,[a] Cristina A. Guevara,[b] Caitlin E. Scott,[b] and George C. Shields[*a]

[a] Department of Chemistry, Furman University, 3300 Poinsett Highway, Greenville, South Carolina 29613, United States

[b] Department of Chemistry and Biochemistry, California State University, Los Angeles, 5151 State University Drive, Los Angeles, California 90032, United States

* email george.shields@furman.edu, caroline.glick@furman.edu

# These authors contributed equally to this research.

Table of Contents

Page	Compound #	Fentanyl Derivative
S-4	1	Fentanyl
S-5	2	Cyclopropyl
S-6	3	p-Methyl cyclopropyl
S-7	4	Cyclobutyl
S-8	5	Cyclopentyl
S-9	6	Furanyl
S-10	7	Tetrahydrofuran
S-12	8	Acetyl
S-13	9	o-Methyl acetyl
S-14	10	α-Methyl acetyl
S-15	11	Acryl
S-16	12	o-Fluoro acryl
S-18	13	p-Fluoro acryl
S-19	14	Isobutyryl
S-20	15	o-Fluoro isobutyryl
S-21	16	m-Fluoro isobutyryl
S-23	17	p-Fluoro isobutyryl
S-24	18	p-Chloro isobutyryl
S-25	19	Pivaloyl
S-26	20	Butyryl
S-28	21	o-Fluoro butryrl
S-31	22	Methoxyacetyl
S-33	23	o-Fluoro
S-35	24	m-Fluoro
S-38	25	p-Fluoro
S-40	26	o-Methyl
S-42	27	m-Methyl
S-44	28	p-Methyl
S-46	29	p-Chloro
S-48	30	Cis-3-methyl
S-50	31	Trans-3-methyl
S-53	32	Furanylethyl
S-55	33	β-Hydroxy
S-57	34	β-Methyl
S-60		PEPCIT10 Structures
S-66		UGIYEP Structures

Table S1 contains a table of the number of conformers in the *cis*, *trans*, and *gauche* conformation with Boltzmann populations above 0.5%,

Figures S1-S34 contain figures of isomers with Boltzmann percentages above 0.5%, organized by each analog.

Labels beneath each structure give the isomer's rank relative to the analog's global minimum, the relative Gibbs free energy computed with DLPNO-CCSD(T)/CBS(DTQ)/SMD//ωB97X-D/6-31++G**/SMD, the Boltzmann percentage, the RMSD relative to the global minimum structure, and the relative Gibbs free energy computed with DLPNO-CCSD(T)/CBS(haDTQ)/SMD//ωB97X-D/6-31++G**/SMD if the Boltzmann percentage is above 5%.

Analog Number	Structure	Cis	Trans	Gauche
1	Fentanyl	6	3	4
2	Cyclopropyl	6	5	4
3	p-Methyl cyclopropyl	5	0	2
4	Cyclobutyl	7	1	3
5	Cyclopentyl	7	0	2
6	Furanyl	6	2	5
7	Tetrahydrofuran	10	2	7
8	Acetyl	3	1	3
9	o-Methyl acetyl	5	2	6
10	α-Methyl acetyl	3	0	2
11	Acryl	7	1	4
12	o-Fluoro acryl	9	3	4
13	p-Fluoro acryl	7	2	5
14	Isobutyryl	7	1	5
15	o-Fluoro isobutyryl	5	1	5
16	m-Fluoro isobutyryl	14	3	4
17	p-Fluoro isobutyryl	8	2	4
18	p-Chloro isobutyryl	9	2	1
19	Pivaloyl	8	1	3
20	Butyryl	11	0	6
21	o-Fluoro butryrl	20	0	13
22	Methoxyacetyl	12	1	6
23	o-Fluoro	12	1	11
24	m-Fluoro	18	3	9
25	p-Fluoro	12	1	4
26	o-Methyl	14	1	3
27	m-Methyl	14	2	2
28	p-Methyl	10	1	5
29	p-Chloro	14	1	3
30	Cis-3-methyl	7	1	4
31	Trans-3-methyl	7	7	12
32	Furanylethyl	7	2	8
33	β-Hydroxy	17	2	1
34	β-Methyl	6	10	9

Table S1. Number of conformers in the *cis*, *trans*, and *gauche* conformation with Boltzmann populations above 0.5%, organized by each analog. Boltzmann populations are computed using ΔGs of DLPNO-CCSD(T)/CBS(DTQ)/SMD//ωB97X-D/6-31++G**/SMD model chemistry.

This data was used to make Figure 4 in the text.

Figure S1. Fentanyl

Structure #

ΔG (310.15K)

Boltzmann %

RMSD

ΔG (310.15K/haug)

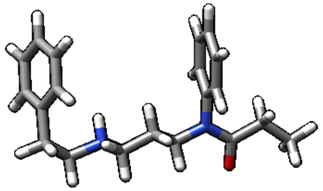

Structure 1

0.00 kcal/mol

50.32 %

0.00 Å

0.00 kcal/mol

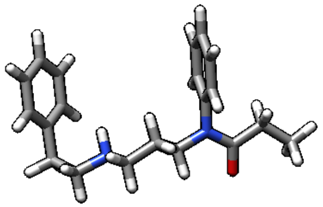

Structure 2

0.45 kcal/mol

24.30 %

0.80 Å

0.53 kcal/mol

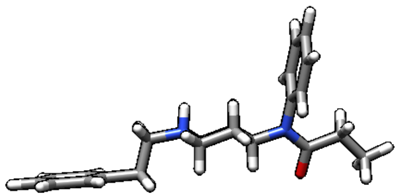

Structure 3

1.29 kcal/mol

6.24 %

2.04 Å

0.94 kcal/mol

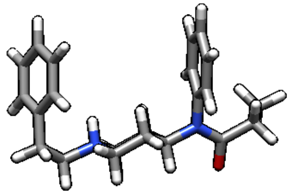

Structure 4

1.70 kcal/mol

3.18 %

0.39 Å

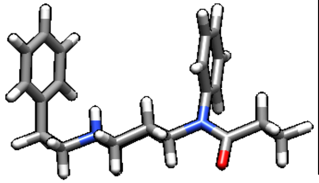

Structure 5

1.74 kcal/mol

3.02 %

0.39 Å

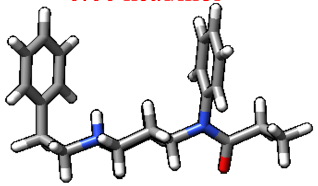

Structure 6

1.75 kcal/mol

2.94 %

0.81 Å

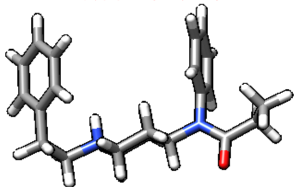

Structure 7

2.00 kcal/mol

1.96 %

0.95 Å

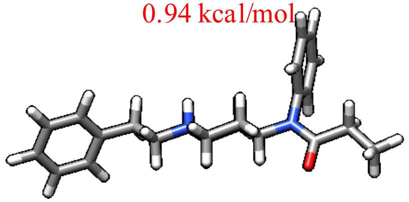

Structure 8

2.06 kcal/mol

1.77 %

1.65 Å

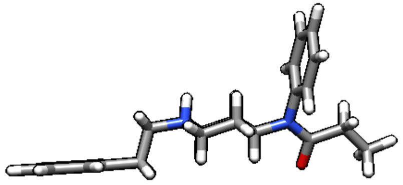

Structure 9

2.67 kcal/mol

0.66 %

2.04 Å

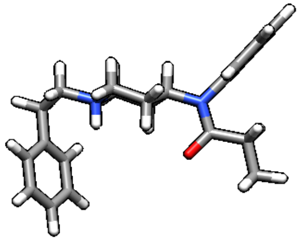

Structure 10

2.68 kcal/mol

0.66 %

1.43 Å

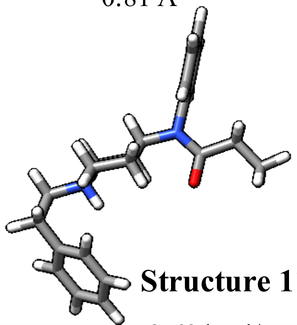

Structure 11

2.68 kcal/mol

0.66 %

1.43 Å

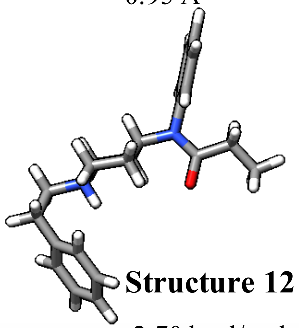

Structure 12

2.70 kcal/mol

0.63 %

1.53 Å

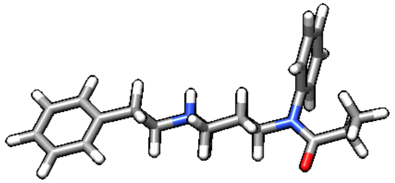

Structure 13

2.80 kcal/mol

0.54 %

1.88 Å

Figure S2. Cyclopropyl fentanyl

Structure #
ΔG (310.15K)
Boltzmann %
RMSD
ΔG (310.15K/haug)

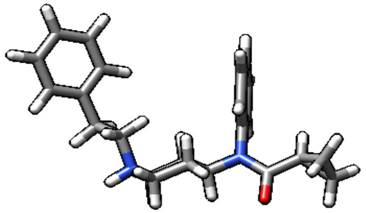

Structure 1
0.00 kcal/mol
20.78 %
0.00 Å
0.00 kcal/mol

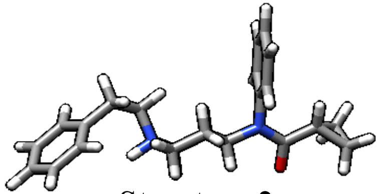

Structure 2
0.22 kcal/mol
14.53 %
1.47 Å
0.56 kcal/mol

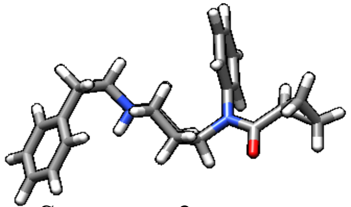

Structure 3
0.23 kcal/mol
14.36 %
1.91 Å
0.93 kcal/mol

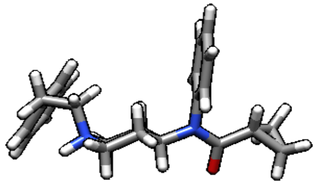

Structure 4
0.51 kcal/mol
9.05 %
1.60 Å
0.70 kcal/mol

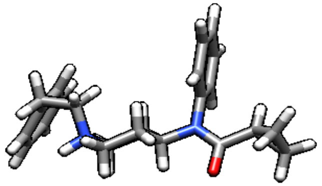

Structure 5
0.60 kcal/mol
7.82 %
1.15 Å
0.79 kcal/mol

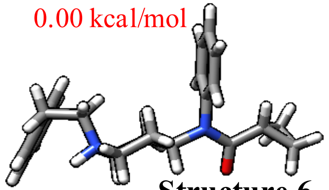

Structure 6
0.74 kcal/mol
6.23 %
1.77 Å
0.90 kcal/mol

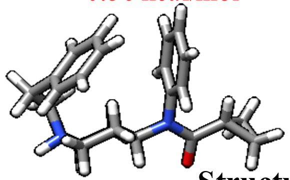

Structure 7
0.79 kcal/mol
5.80 %
1.28 Å
0.81 kcal/mol

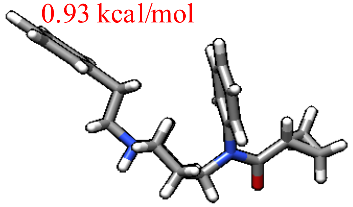

Structure 8
0.90 kcal/mol
4.84 %
1.11 Å

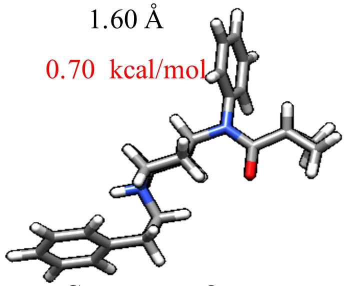

Structure 9
1.18 kcal/mol
3.07 %
2.07 Å

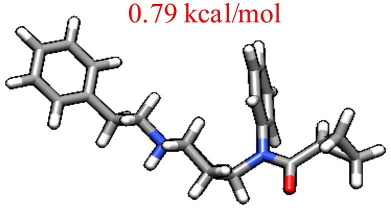

Structure 10
1.26 kcal/mol
2.71 %
0.79 Å

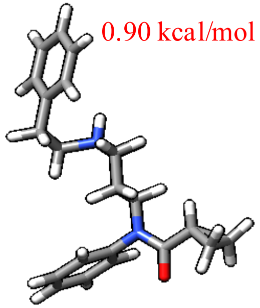

Structure 11
1.54 kcal/mol
1.72 %
2.14 Å

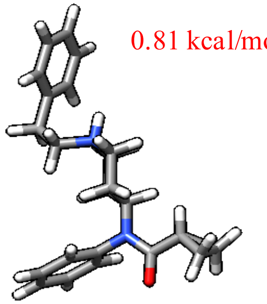

Structure 12
1.54 kcal/mol
1.71%
1.97 Å

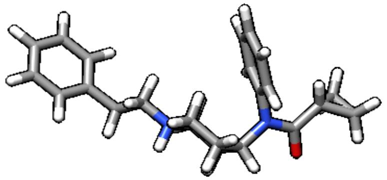

Structure 13
1.58 kcal/mol
1.61 %
1.20 Å

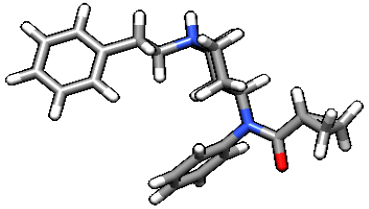

Structure 14
1.80 kcal/mol
1.12 %
1.24 Å

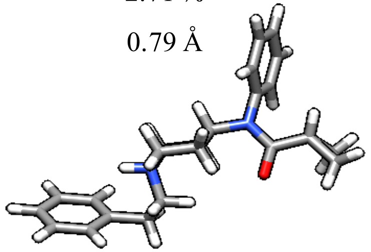

Structure 15
1.83 kcal/mol
1.07 %
2.17 Å

Figure S3. p-Methyl cyclopropyl fentanyl

Structure #
ΔG (310.15K)
Boltzmann %
RMSD
ΔG (310.15K/haug)

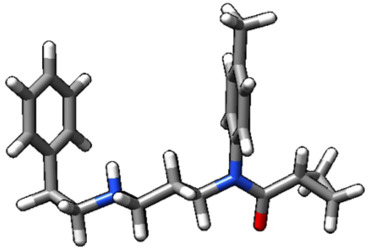

Structure 1
0.00 kcal/mol
54.63 %
0.00 Å
0.00 kcal/mol

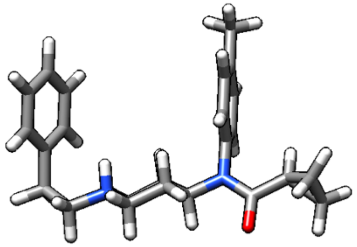

Structure 2
0.52 kcal/mol
23.37 %
0.80 Å
0.47 kcal/mol

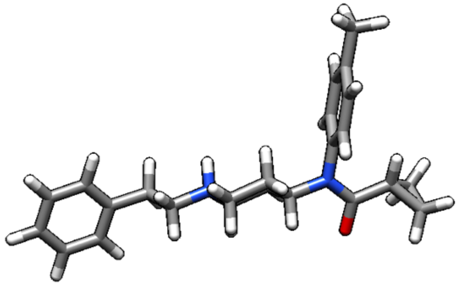

Structure 3
1.21 kcal/mol
7.70 %
1.69 Å
1.09 kcal/mol

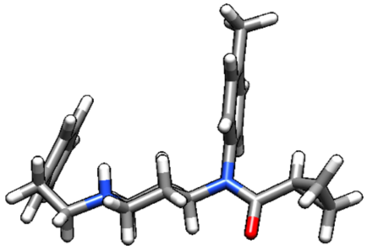

Structure 4
1.81 kcal/mol
2.92 %
1.07 Å

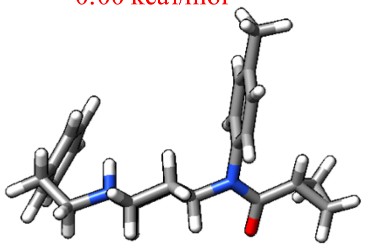

Structure 5
1.80 kcal/mol
2.93 %
1.16 Å

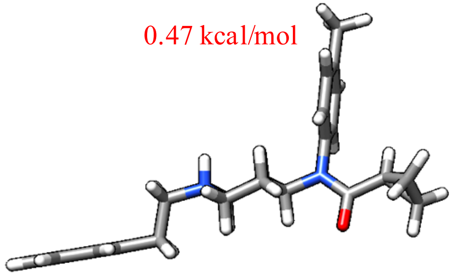

Structure 6
1.64 kcal/mol
3.83 %
1.97 Å

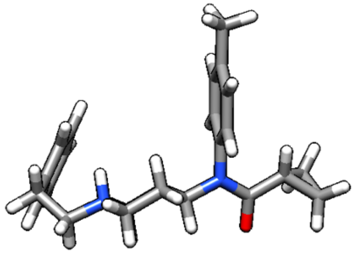

Structure 7
2.06 kcal/mol
1.93 %
1.04 Å

Figure S4. Cyclobutyl fentanyl

Structure #
ΔG (310.15K)
Boltzmann %
RMSD
ΔG (310.15K/haug)

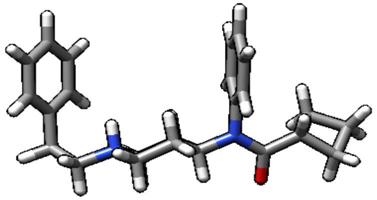

Structure 1
0.00 kcal/mol
39.24 %
0.00 Å
0.00 kcal/mol

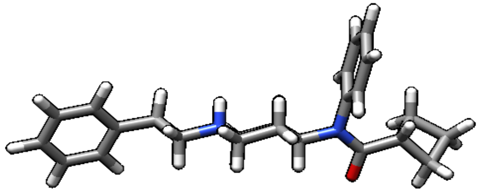

Structure 2
0.61 kcal/mol
14.61 %
1.63 Å
0.55 kcal/mol

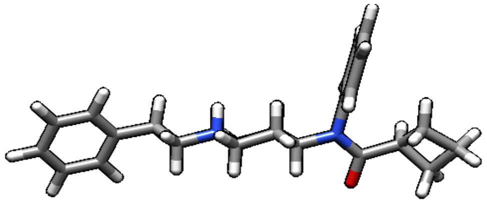

Structure 3
0.63 kcal/mol
14.04 %
1.71 Å
0.62 kcal/mol

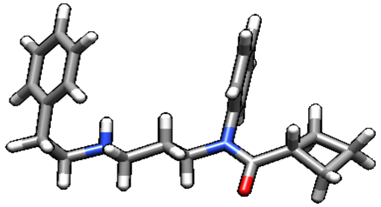

Structure 4
0.87 kcal/mol
9.52 %
0.53 Å
1.04 kcal/mol

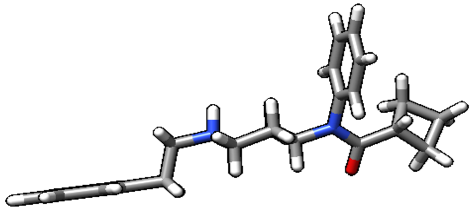

Structure 5
1.31 kcal/mol
4.67 %
2.05 Å

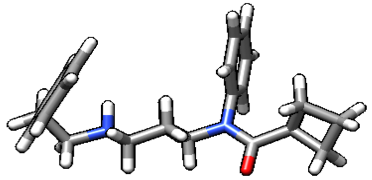

Structure 6
1.51 kcal/mol
3.40 %
1.36 Å

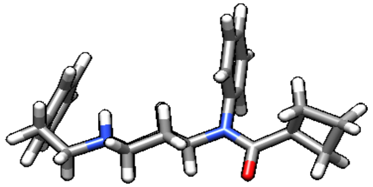

Structure 7
1.56 kcal/mol
3.12 %
1.21 Å

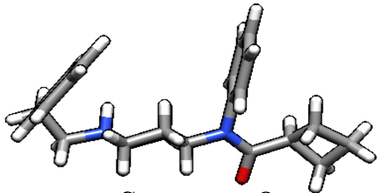

Structure 8
1.64 kcal/mol
2.74 %
1.04 Å

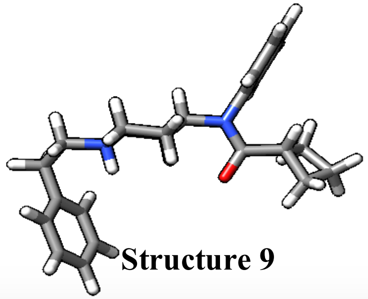

Structure 9
1.74 kcal/mol
2.33 %
1.42 Å

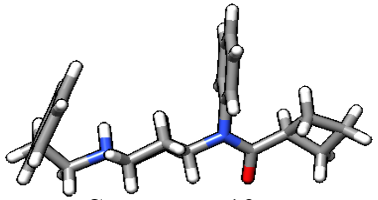

Structure 10
1.80 kcal/mol
2.12 %
1.02 Å

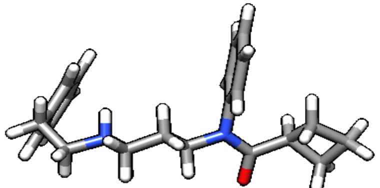

Structure 11
2.07 kcal/mol
1.37 %
1.16 Å

Figure S5. Cyclopentyl fentanyl

Structure #
ΔG (310.15K)
Boltzmann %
RMSD
ΔG (310.15K/haug)

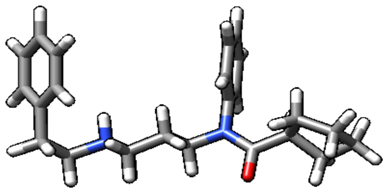

Structure 1
0.00 kcal/mol
26.77 %
0.00 Å
0.00 kcal/mol

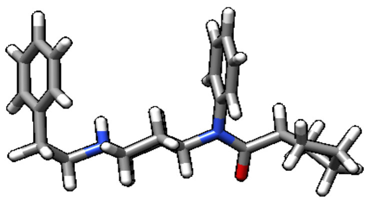

Structure 2
0.07 kcal/mol
24.04 %
1.11 Å
0.18 kcal/mol

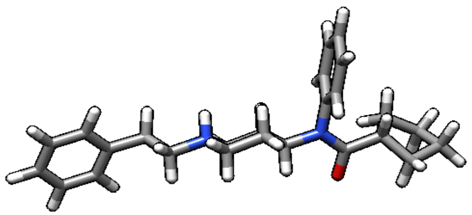

Structure 3
0.29 kcal/mol
16.60 %
1.54 Å
0.20 kcal/mol

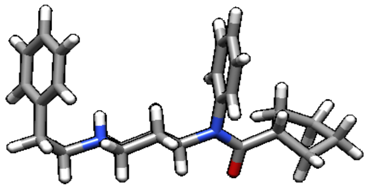

Structure 4
0.69 kcal/mol
8.68%
0.74 Å
0.48 kcal/mol

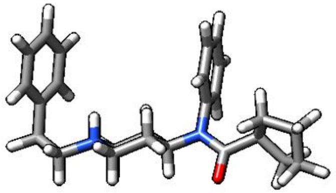

Structure 5
0.79 kcal/mol
7.48 %
0.59 Å
0.62 kcal/mol

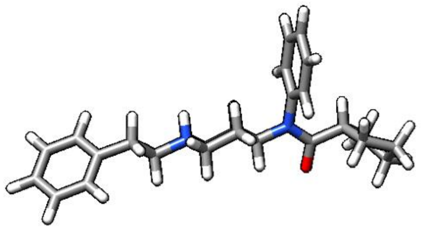

Structure 6
1.13 kcal/mol
4.27 %
0.78 Å

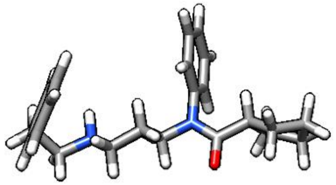

Structure 7
1.54 kcal/mol
2.20 %
1.89 Å

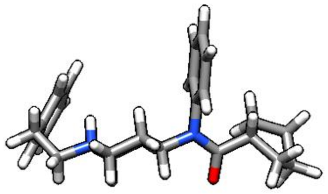

Structure 8
1.98 kcal/mol
1.07 %
1.45 Å

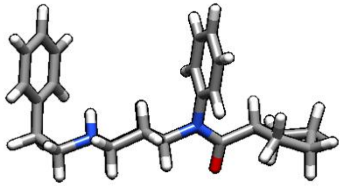

Structure 9
1.99 kcal/mol
1.07 %
1.42 Å

Figure S6. Furanyl fentanyl

Structure #
ΔG (310.15K)
Boltzmann %
RMSD
ΔG (310.15K/haug)

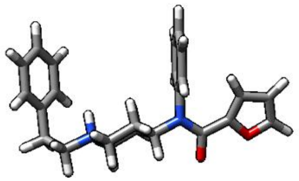

Structure 1
0.00 kcal/mol
31.72 %
0.00 Å
0.00 kcal/mol

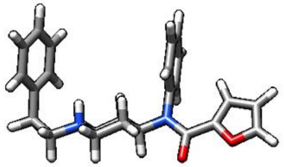

Structure 2
0.44 kcal/mol
15.63 %
0.79 Å
0.46 kcal/mol

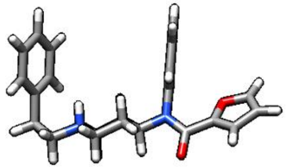

Structure 3
0.46 kcal/mol
15.02 %
0.98 Å
0.39 kcal/mol

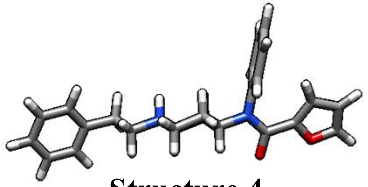

Structure 4
0.86 kcal/mol
7.87 %
1.66 Å
0.77 kcal/mol

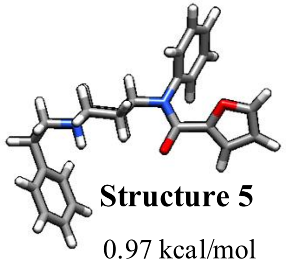

Structure 5
0.97 kcal/mol
6.54 %
1.80 Å
0.66 kcal/mol

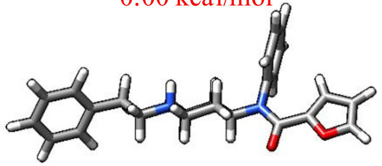

Structure 6
1.11 kcal/mol
5.24 %
1.86 Å
0.90 kcal/mol

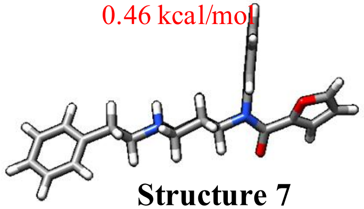

Structure 7
1.31 kcal/mol
3.79 %
1.93 Å

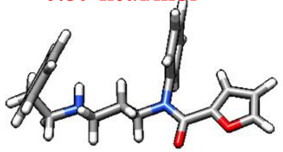

Structure 8
1.69 kcal/mol
2.04 %
1.55 Å

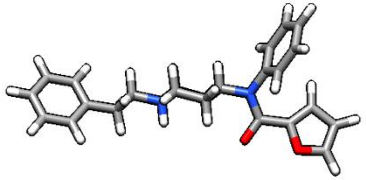

Structure 9
1.82 kcal/mol
1.66 %
2.19 Å

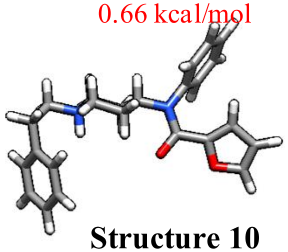

Structure 10
1.91 kcal/mol
1.44 %
1.90 Å

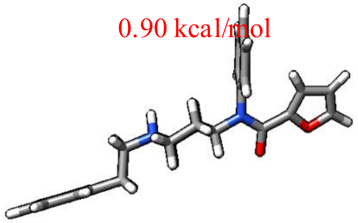

Structure 11
2.05 kcal/mol
1.15 %
2.02 Å

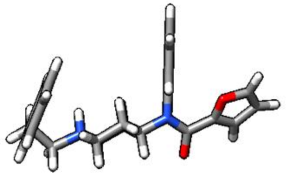

Structure 12
2.08 kcal/mol
1.09 %
1.26 Å

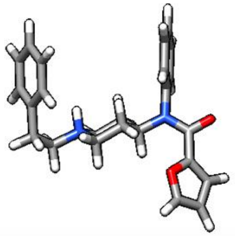

Structure 13
2.11 kcal/mol
1.03 %
1.49 Å

Figure S7. Tetrahydrofuran fentanyl

Structure #
ΔG (310.15K)
Boltzmann %
RMSD
ΔG (310.15K/haug)

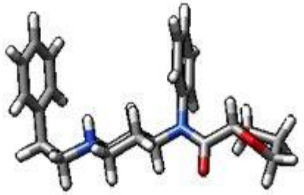

Structure 1
0.00 kcal/mol
20.45 %
0.00 Å
0.00 kcal/mol

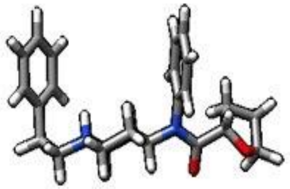

Structure 2
0.16 kcal/mol
15.79 %
1.26 Å
0.05 kcal/mol

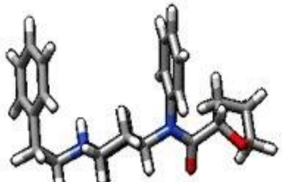

Structure 3
0.32 kcal/mol
12.13 %
0.89 Å
0.17 kcal/mol

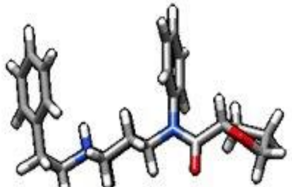

Structure 4
0.38 kcal/mol
11.10 %
0.77 Å
0.24 kcal/mol

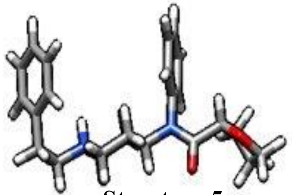

Structure 5
0.69 kcal/mol
6.73 %
0.87 Å
0.73 kcal/mol

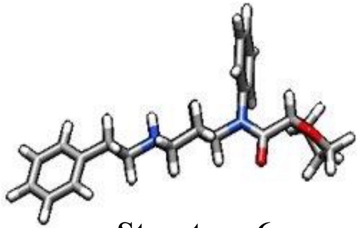

Structure 6
0.95 kcal/mol
4.39 %
1.84 Å

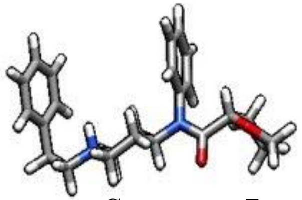

Structure 7
1.01 kcal/mol
3.99 %
0.38 Å

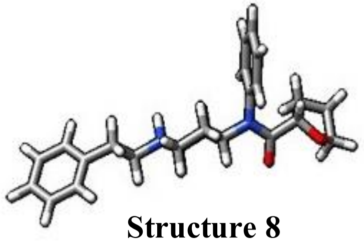

Structure 8
1.09 kcal/mol
3.52 %
1.73 Å

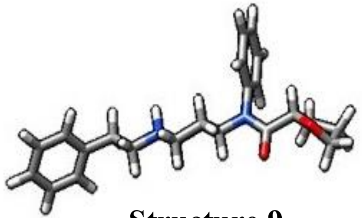

Structure 9
1.16 kcal/mol
3.14 %
1.56 Å

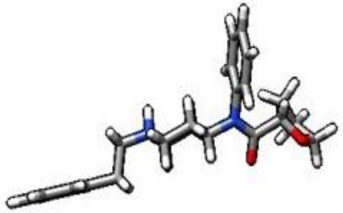

Structure 10
1.21 kcal/mol
2.86 %
2.05 Å

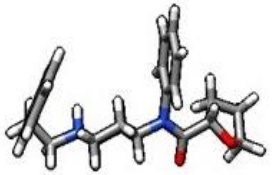

Structure 11
1.27 kcal/mol
2.61 %
0.68 Å

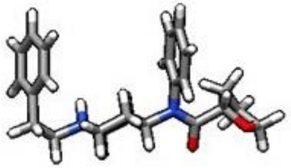

Structure 12
1.29 kcal/mol
2.51 %
0.97 Å

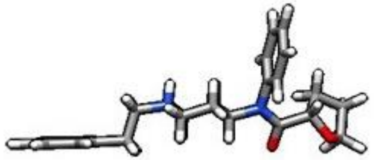

Structure 13
1.61 kcal/mol
1.51 %
1.90 Å

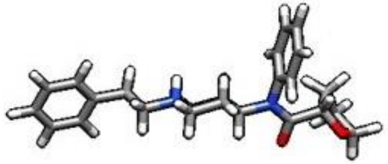

Structure 14
1.77 kcal/mol
1.16 %
1.96 Å

Figure S7. Tetrahydrofuran fentanyl

Structure #
ΔG (310.15K)
Boltzmann %
RMSD
ΔG (310.15K/haug)

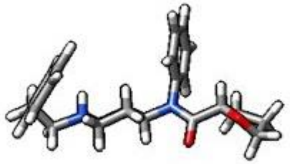

Structure 15
1.86 kcal/mol
1.01 %
1.45 Å

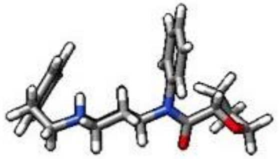

Structure 16
1.95 kcal/mol
0.86 %
1.77 Å

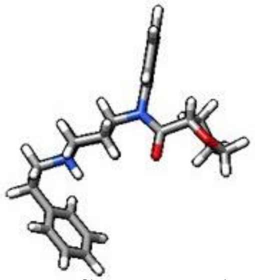

Structure 17
2.07 kcal/mol
0.71 %
1.55 Å

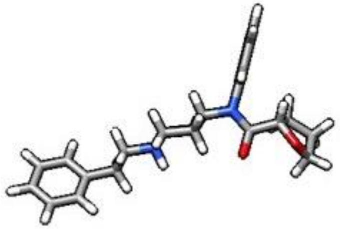

Structure 18
2.15 kcal/mol
0.63 %
2.21 Å

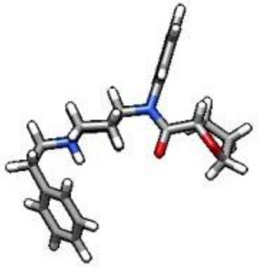

Structure 19
2.19 kcal/mol
0.59 %
1.68 Å

Figure S8. Acetyl fentanyl

Structure #
ΔG (310.15K)
Boltzmann %
RMSD
ΔG (310.15K/haug)

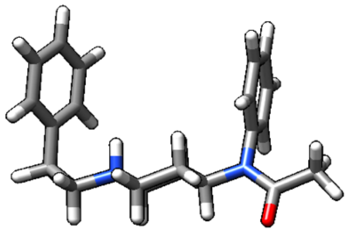

Structure 1
0.00 kcal/mol
76.44 %
0.00 Å
0.00 kcal/mol

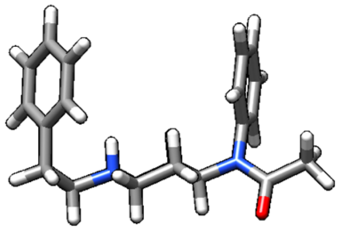

Structure 2
1.25 kcal/mol
10.09 %
0.84 Å
1.55 kcal/mol

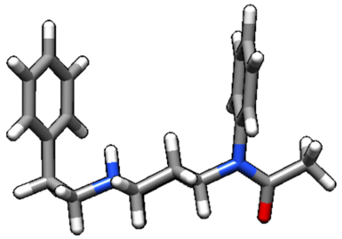

Structure 3
1.58 kcal/mol
5.86 %
0.79 Å
1.78 kcal/mol

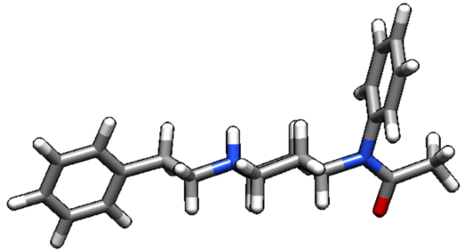

Structure 4
2.08 kcal/mol
2.64 %
1.91 Å

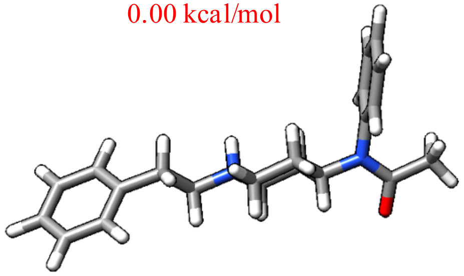

Structure 5
2.15 kcal/mol
2.33 %
1.69 Å

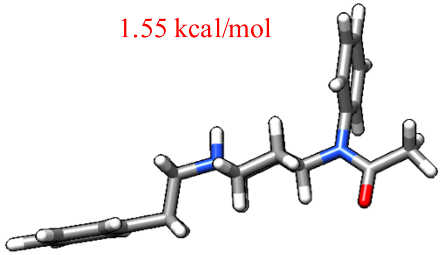

Structure 6
2.90 kcal/mol
0.69 %
2.09 Å

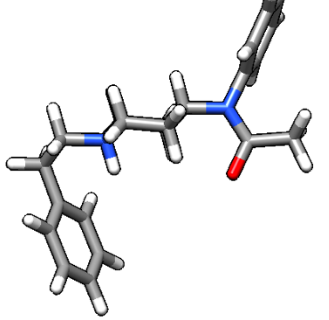

Structure 7
2.98 kcal/mol
0.61 %
1.58 Å

Figure S9. o-Methyl acetyl fentanyl

Structure #
ΔG (310.15K)
Boltzmann %
RMSD
ΔG (310.15K/haug)

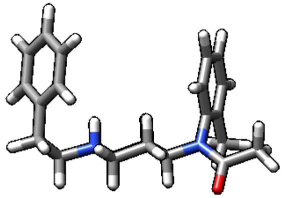

Structure 1
0.00 kcal/mol
43.07 %
0.00 Å
0.00 kcal/mol

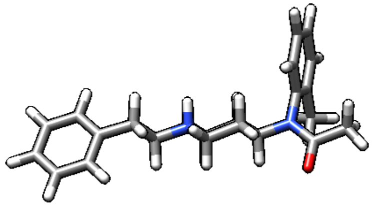

Structure 2
0.61 kcal/mol
16.07 %
1.57 Å
0.45 kcal/mol

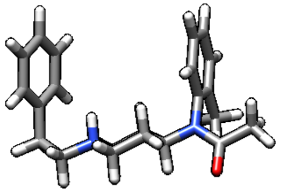

Structure 3
0.71 kcal/mol
13.65 %
0.85 Å
0.45 kcal/mol

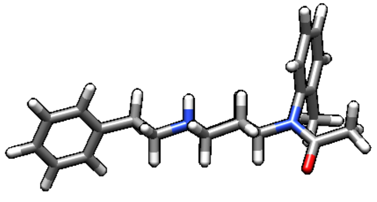

Structure 4
1.17 kcal/mol
6.43 %
1.84 Å
0.91 kcal/mol

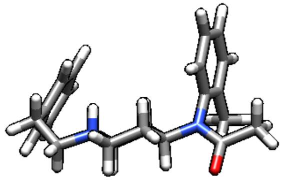

Structure 5
1.18 kcal/mol
6.40 %
1.22 Å
0.87 kcal/mol

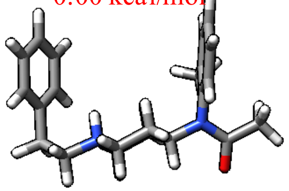

Structure 6
1.41 kcal/mol
4.39 %
0.91 Å

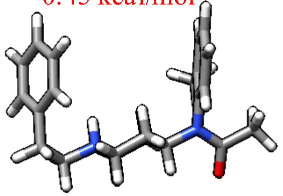

Structure 7
1.76 kcal/mol
2.48 %
1.08 Å

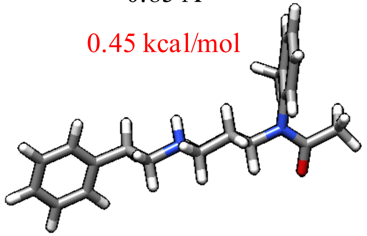

Structure 8
2.05 kcal/mol
1.56 %
1.79 Å

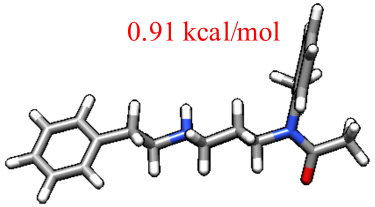

Structure 9
2.29 kcal/mol
1.05 %
1.90 Å

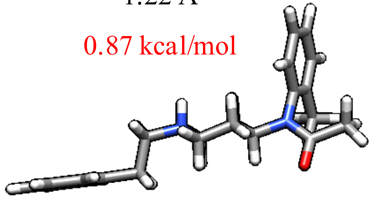

Structure 10
2.39 kcal/mol
0.89 %
1.99 Å

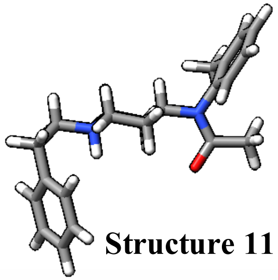

Structure 11
2.46 kcal/mol
0.80 %
1.46 Å

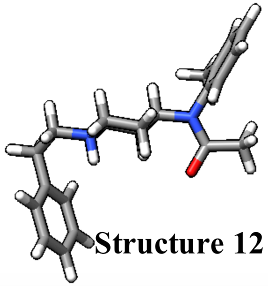

Structure 12
2.64 kcal/mol
0.60 %
1.64 Å

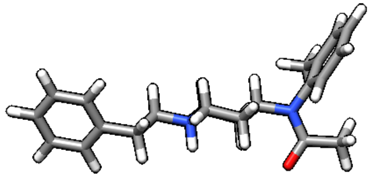

Structure 13
2.68 kcal/mol
0.56 %
1.96 Å

Figure S10. α-Methyl acetyl fentanyl

Structure #

ΔG (310.15K)

Boltzmann %

RMSD

ΔG (310.15K/haug)

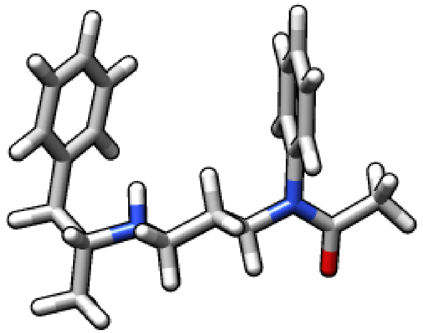

Structure 1

0.00 kcal/mol

55.80 %

0.00 Å

0.00 kcal/mol

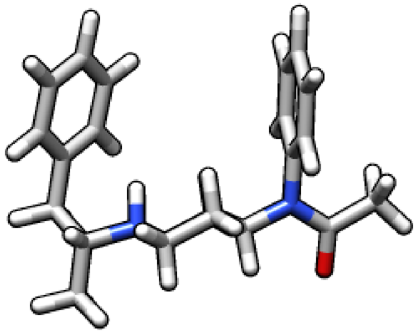

Structure 2

0.31 kcal/mol

33.91 %

1.46 Å

0.44 kcal/mol

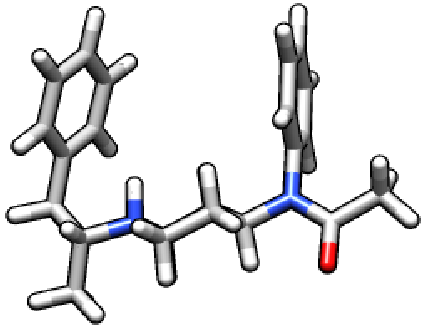

Structure 3

1.50 kcal/mol

4.93 %

1.63 Å

1.47 kcal/mol

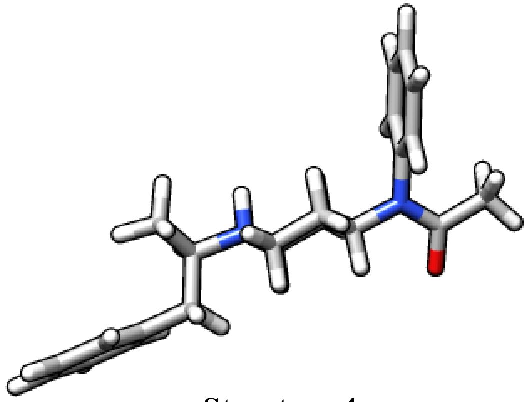

Structure 4

1.81 kcal/mol

2.95 %

1.89 Å

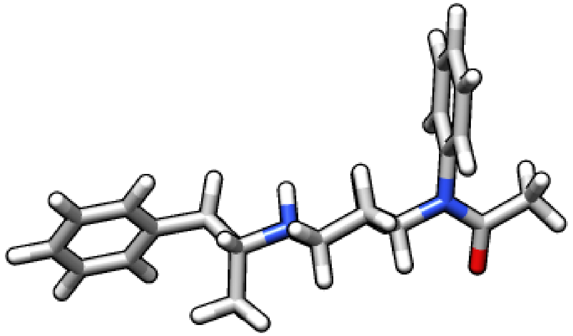

Structure 5

2.79 kcal/mol

0.61 %

1.78 Å

Figure S11. Acryl fentanyl

Structure #
ΔG (310.15K)
Boltzmann %
RMSD
ΔG (310.15K/haug)

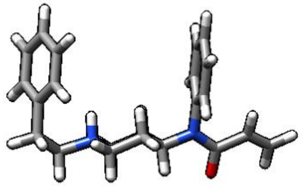

Structure 1
0.00 kcal/mol
59.01 %
0.00 Å
0.00 kcal/mol

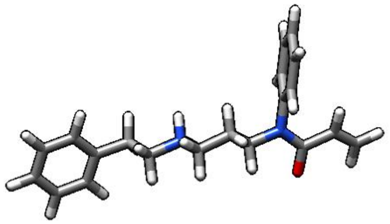

Structure 2
1.20 kcal/mol
8.38 %
1.91 Å
0.99 kcal/mol

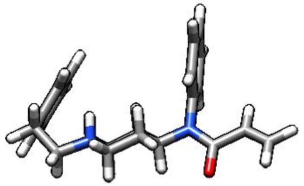

Structure 3
1.25 kcal/mol
7.75 %
0.78 Å
1.04 kcal/mol

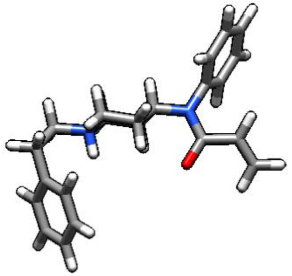

Structure 4
1.35 kcal/mol
6.57 %
1.45 Å
1.04 kcal/mol

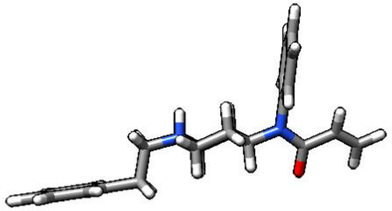

Structure 5
1.61 kcal/mol
4.31 %
2.08 Å

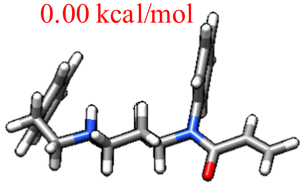

Structure 6
1.62 kcal/mol
4.29 %
0.84 Å

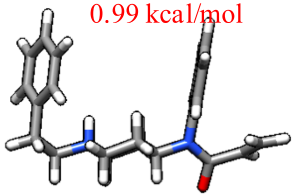

Structure 7
2.16 kcal/mol
1.79 %
0.50 Å

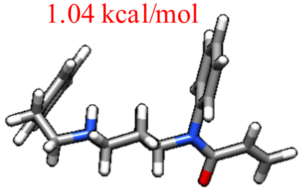

Structure 8
2.18 kcal/mol
1.72 %
1.26 Å

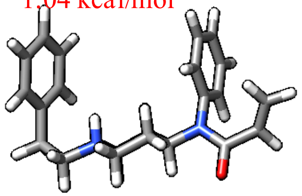

Structure 9
2.24 kcal/mol
1.55 %
0.55 Å

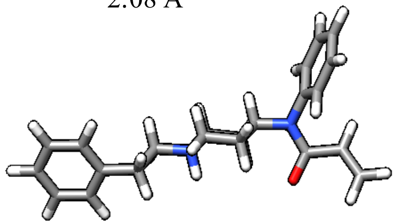

Structure 10
2.60 kcal/mol
0.87%
2.20 Å

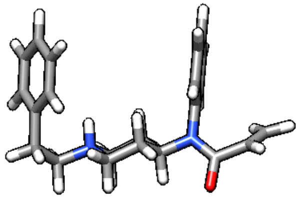

Structure 11
2.65 kcal/mol
0.81 %
0.83 Å

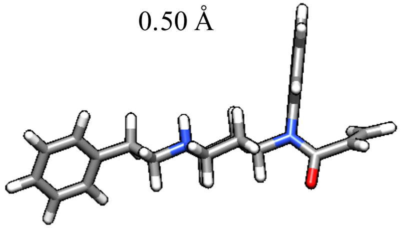

Structure 12
2.91 kcal/mol
0.53 %
1.71 Å

Figure S12. o-Fluoro acryl fentanyl

Structure #
ΔG (310.15K)
Boltzmann %
RMSD
ΔG (310.15K/haug)

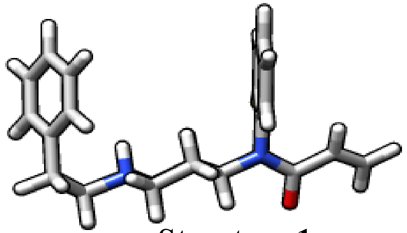

Structure 1
0.00 kcal/mol
42.77 %
0.00 Å
0.00 kcal/mol

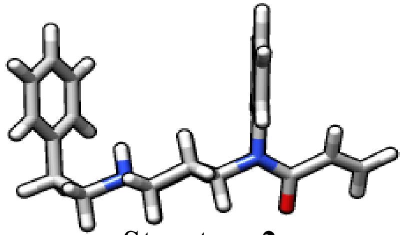

Structure 2
0.53 kcal/mol
18.21 %
0. 79Å
0.58 kcal/mol

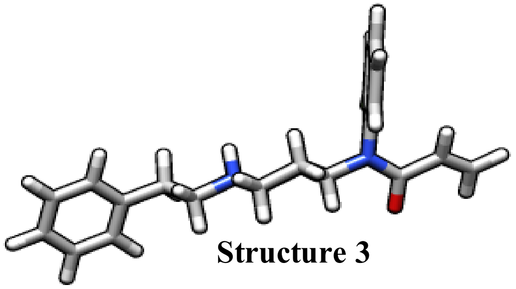

Structure 3
1.07 kcal/mol
7.59 %
1.90 Å
0.77 kcal/mol

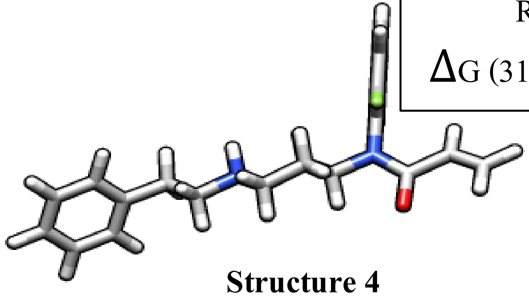

Structure 4
1.19 kcal/mol
6.21 %
1.67 Å
1.00 kcal/mol

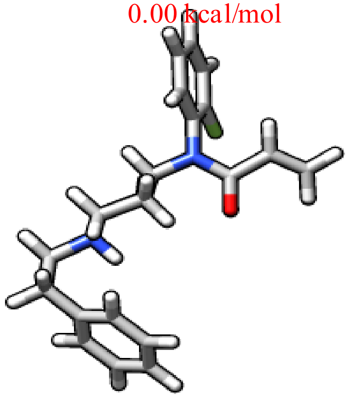

Structure 5
1.57 kcal/mol
3.37 %
1.46 Å

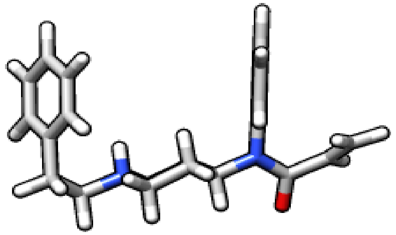

Structure 6
1.60 kcal/mol
3.18 %
0.43 Å

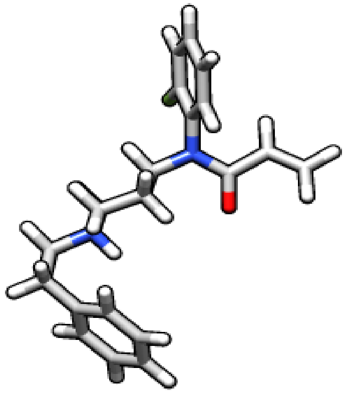

Structure 7
1.63 kcal/mol
3.03 %
1.55 Å

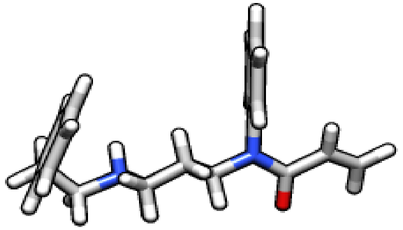

Structure 8
1.89 kcal/mol
2.01 %
1.08 Å

Figure S12. o-Fluoro acryl fentanyl

Structure #
ΔG (310.15K)
Boltzmann %
RMSD
ΔG (310.15K/haug)

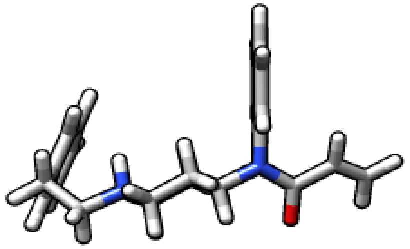

Structure 9
2.00 kcal/mol
1.67 %
1.39 Å

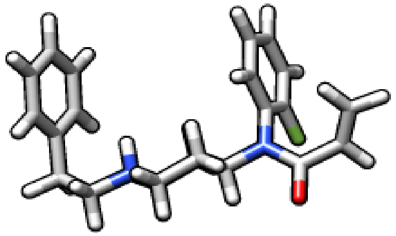

Structure 10
2.02 kcal/mol
1.61 %
1.04 Å

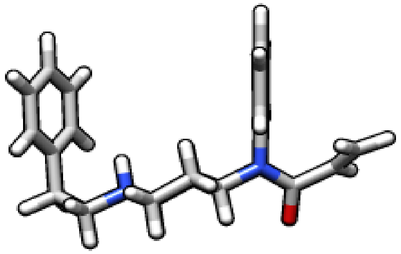

Structure 11
2.25 kcal/mol
1.12 %
0.81 Å

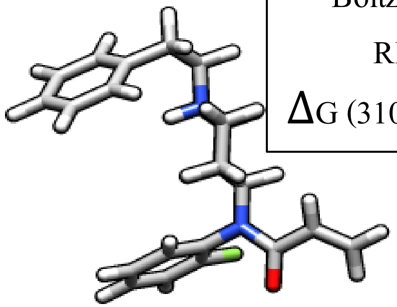

Structure 12
2.29 kcal/mol
1.04 %
1.40 Å

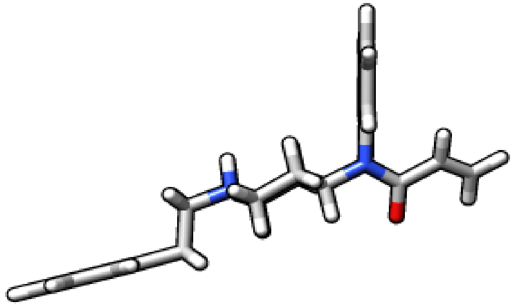

Structure 13
2.41 kcal/mol
0.86 %
2.08 Å

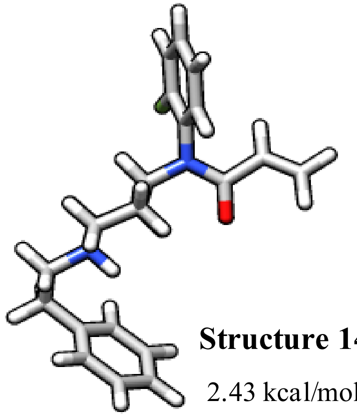

Structure 14
2.43 kcal/mol
0.84 %
1.64 Å

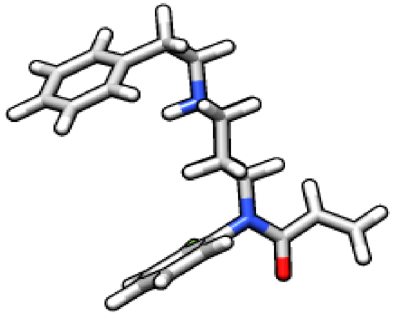

Structure 15
2.67 kcal/mol
0.56 %
0.93 Å

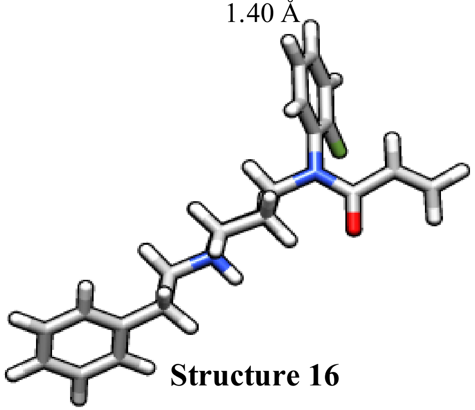

Structure 16
2.69 kcal/mol
0.55 %
2.29 Å

Figure S13. p-Fluoro acryl fentanyl

Structure #
ΔG (310.15K)
Boltzmann %
RMSD
ΔG (310.15K/haug)

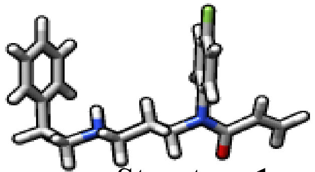

Structure 1
0.00 kcal/mol
34.50 %
0.00 Å
0.02 kcal/mol

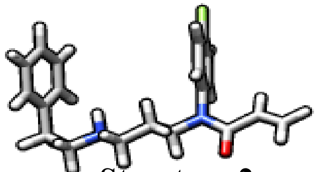

Structure 2
0.23 kcal/mol
23.67%
0.82 Å
0.23 kcal/mol

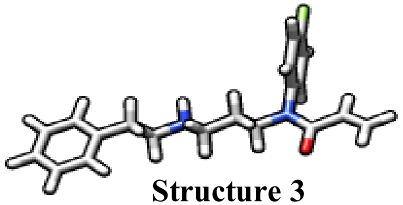

Structure 3
0.35 kcal/mol
19.44 %
1.66 Å
0.00 kcal/mol

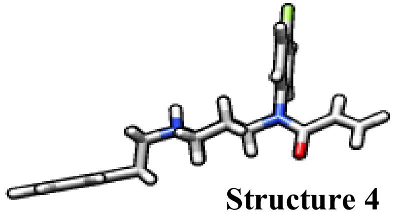

Structure 4
1.23 kcal/mol
4.71 %
2.06 Å

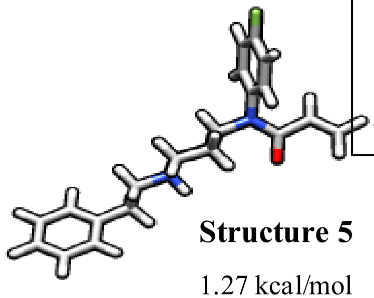

Structure 5
1.27 kcal/mol
4.38 %
2.19 Å

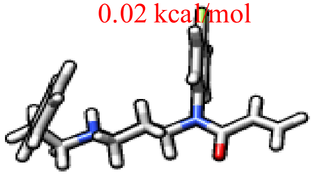

Structure 6
1.58 kcal/mol
2.65 %
1.07 Å

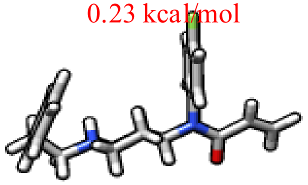

Structure 7
1.86 kcal/mol
1.70 %
1.08 Å

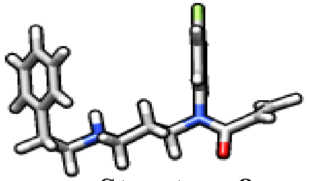

Structure 8
1.93 kcal/mol
1.50 %
0.44 Å

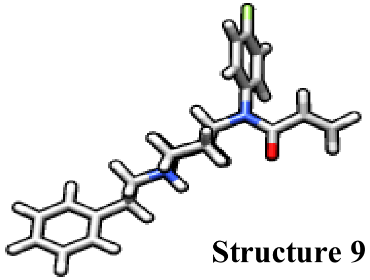

Structure 9
2.08 kcal/mol
1.19 %
2.03 Å

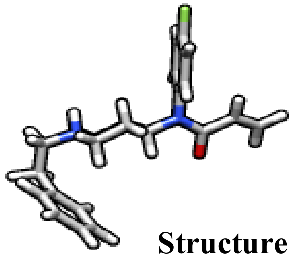

Structure 10
2.20 kcal/mol
0.97 %
1.92 Å

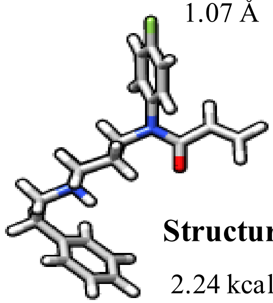

Structure 11
2.24 kcal/mol
0.92 %
1.98 Å

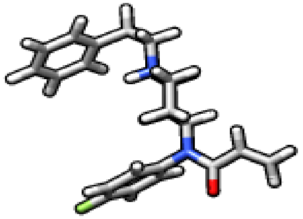

Structure 12
2.36 kcal/mol
0.75 %
1.23 Å

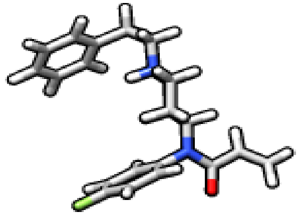

Structure 13
2.37 kcal/mol
0.74 %
0.91 Å

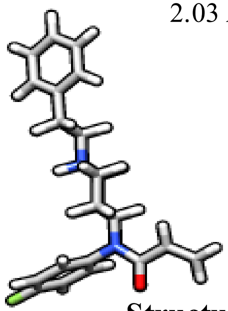

Structure 14
2.44 kcal/mol
0.66 %
1.96 Å

Figure S14. Isobutyryl fentanyl

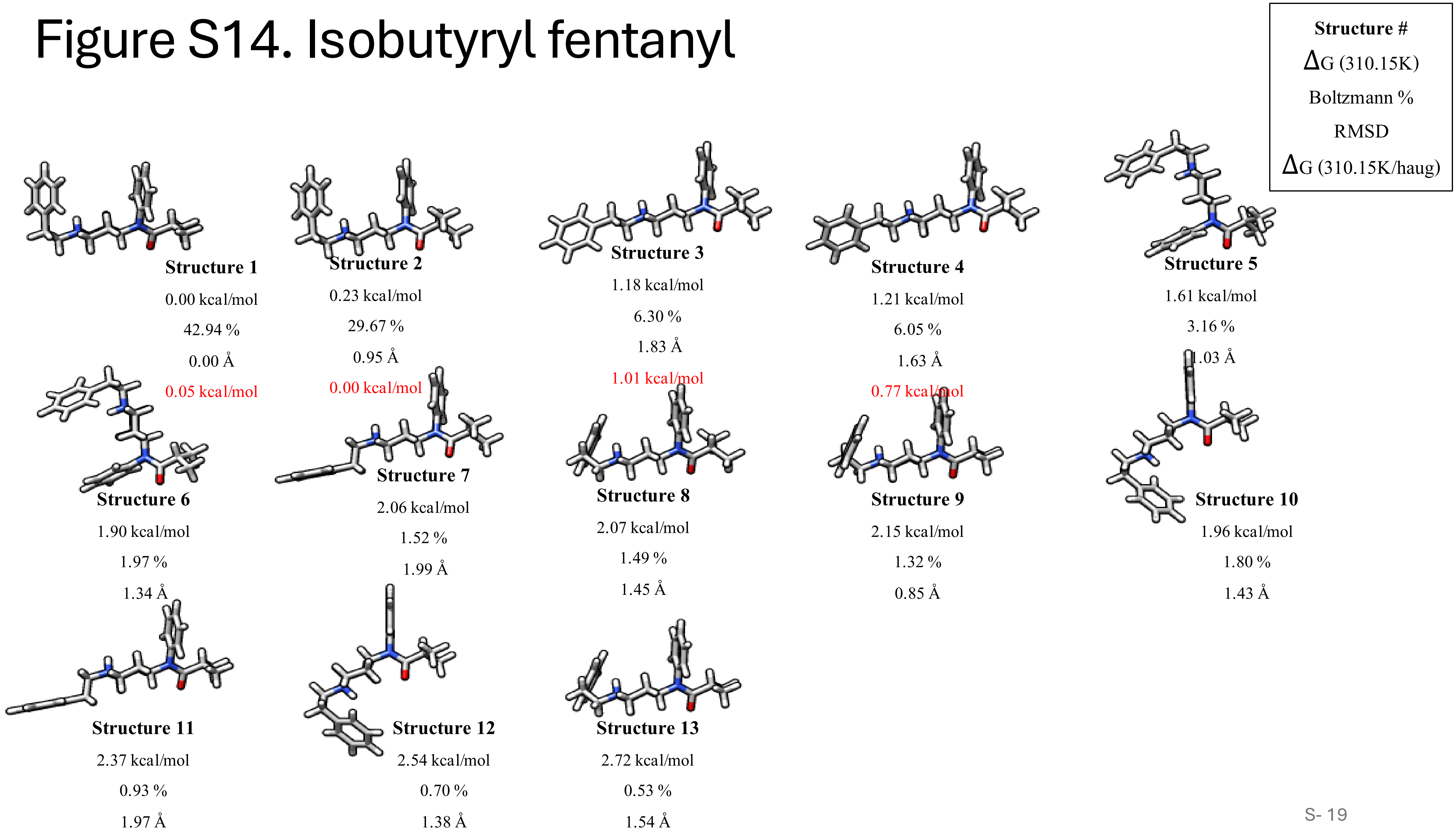

Figure S15. o-Fluoro isobutyryl fentanyl

Structure #
ΔG (310.15K)
Boltzmann %
RMSD
ΔG (310.15K/haug)

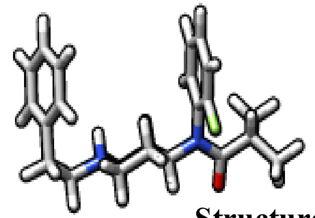

Structure 1
0.00 kcal/mol
26.19%
0.00 Å
0.00 kcal/mol

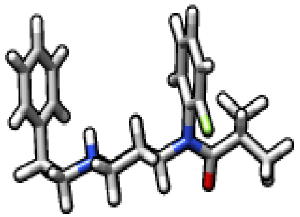

Structure 2
0.05 kcal/mol
24.14 %
0.83 Å
0.83 kcal/mol

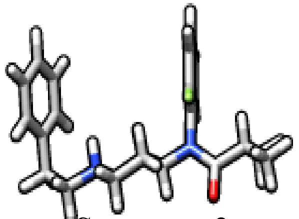

Structure 3
0.06 kcal/mol
23.80 %
0.92 Å
0.00 kcal/mol

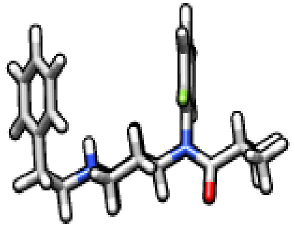

Structure 4
0.56 kcal/mol
10.53 %
0.93 Å
0.58 kcal/mol

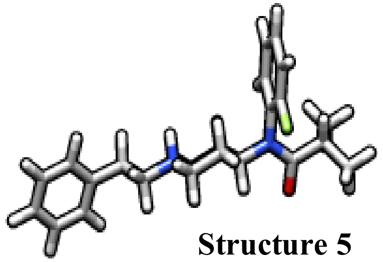

Structure 5
0.74 kcal/mol
7.94 %
1.81 Å
0.35 kcal/mol

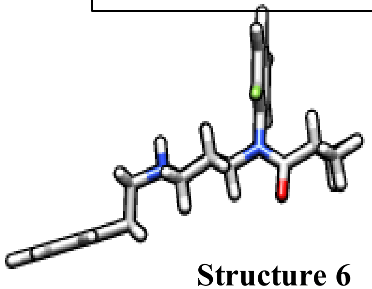

Structure 6
1.89 kcal/mol
1.23 %
2.02 Å

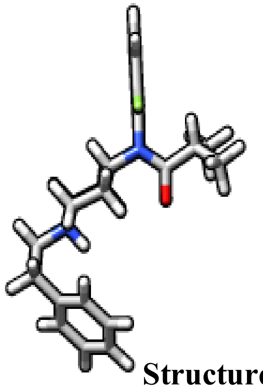

Structure 7
1.96 kcal/mol
1.08 %
1.45 Å

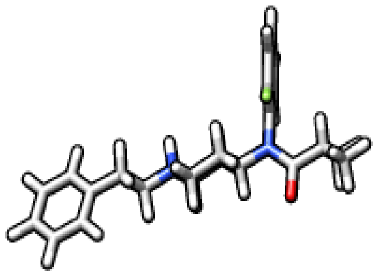

Structure 8
1.97 kcal/mol
1.07 %
1.95 Å

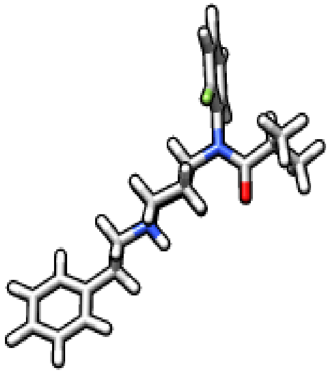

Structure 9
2.16 kcal/mol
0.79 %
2.27 Å

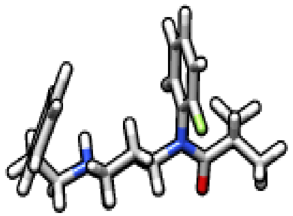

Structure 10
2.21 kcal/mol
0.73 %
0.98 Å

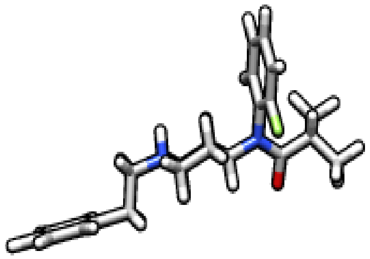

Structure 11
2.21 kcal/mol
0.73 %
2.13 Å

Figure S16. m-Fluoro isobutyryl fentanyl

Structure #

ΔG (310.15K)

Boltzmann %

RMSD

ΔG (310.15K/haug)

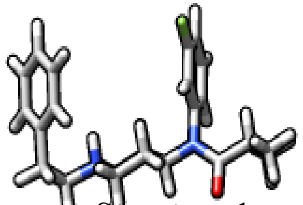

Structure 1

0.00 kcal/mol

27.05 %

0.00 Å

0.05 kcal/mol

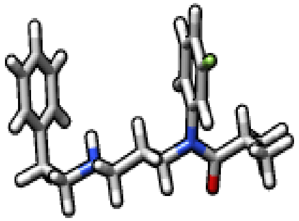

Structure 2

0.22 kcal/mol

18.79 %

0.86 Å

0.00 kcal/mol

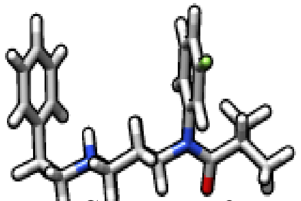

Structure 3

0.31 kcal/mol

16.47 %

0.99 Å

0.43 kcal/mol

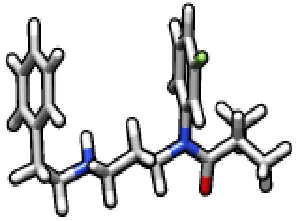

Structure 4

0.83 kcal/mol

7.03 %

1.12 Å

0.50 kcal/mol

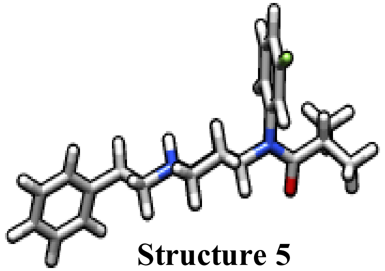

Structure 5

0.96 kcal/mol

5.69 %

1.95 Å

0.23 kcal/mol

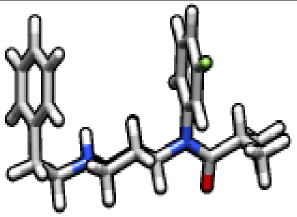

Structure 6

0.97 kcal/mol

5.63 %

1.06 Å

0.76 kcal/mol

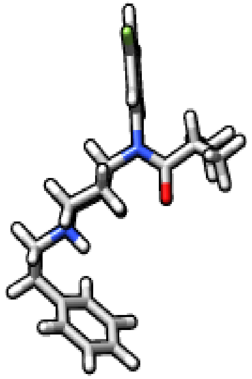

Structure 7

1.37 kcal/mol

2.96 %

1.80 Å

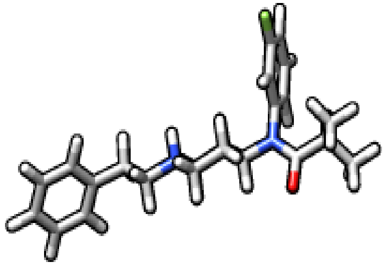

Structure 8

1.54 kcal/mol

2.22 %

1.85 Å

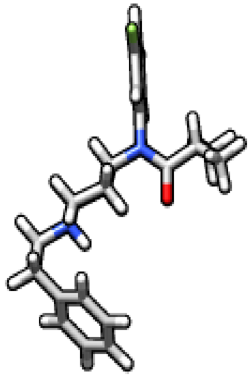

Structure 9

1.68 kcal/mol

1.78 %

1.84 Å

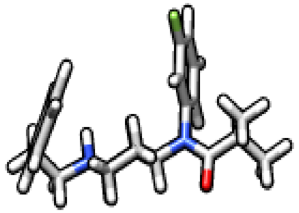

Structure 10

1.93 kcal/mol

1.19 %

1.17 Å

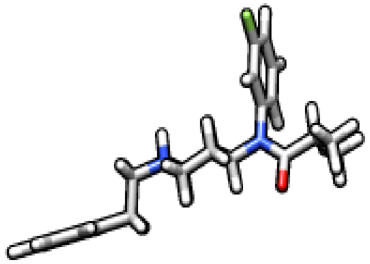

Structure 11

1.93 kcal/mol

1.19 %

1.97 Å

Figure S16. m-Fluoro isobutyryl fentanyl

Structure #
ΔG (310.15K)
Boltzmann %
RMSD
ΔG (310.15K/haug)

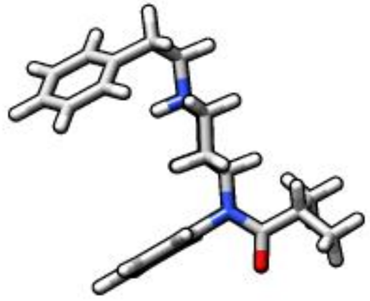

Structure 12
1.98 kcal/mol
1.08 %
1.38 Å

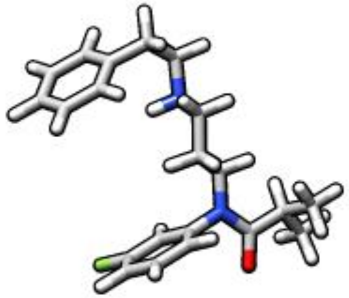

Structure 13
2.07 kcal/mol
0.95 %
1.27 Å

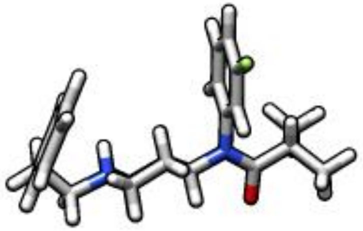

Structure 14
2.07 kcal/mol
0.94 %
1.02 Å

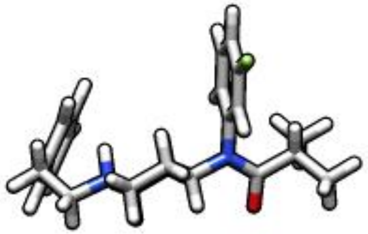

Structure 15
2.09 kcal/mol
0.92 %
1.89 Å

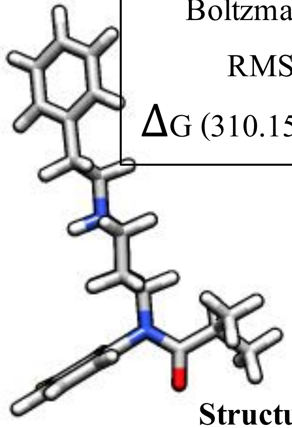

Structure 16
2.10 kcal/mol
0.90 %
2.14 Å

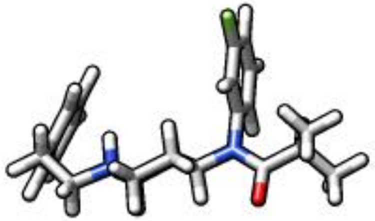

Structure 17
2.19 kcal/mol
0.77 %
1.19 Å

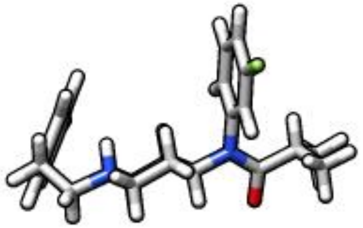

Structure 18
2.23 kcal/mol
0.73 %
1.85 Å

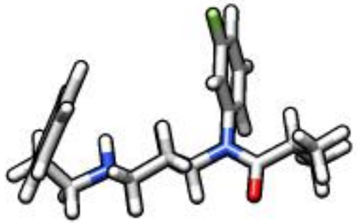

Structure 19
2.27 kcal/mol
0.68 %
1.05 Å

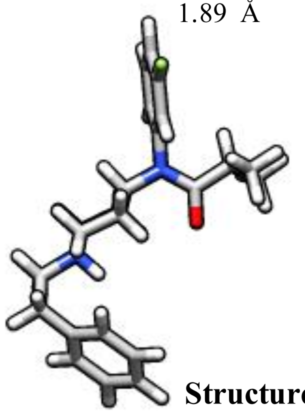

Structure 20
2.33 kcal/mol
0.62 %
1.72 Å

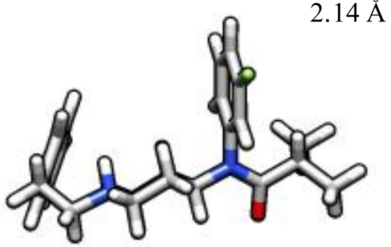

Structure 21
2.39 kcal/mol
0.56 %
1.91 Å

Figure S17. p-Fluoro isobutyryl fentanyl

Structure #
ΔG (310.15K)
Boltzmann %
RMSD
ΔG (310.15K/haug)

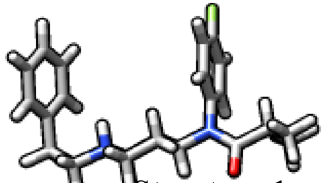

Structure 1
0.00 kcal/mol
36.52 %
0.00 Å
0.00 kcal/mol

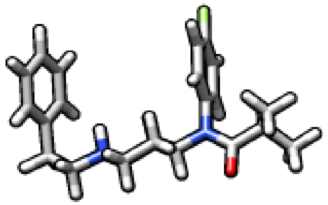

Structure 2
0.01 kcal/mol
36.14 %
0.47 Å
0.12 kcal/mol

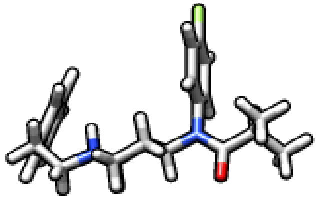

Structure 3
1.14 kcal/mol
5.76 %
0.72 Å
1.29 kcal/mol

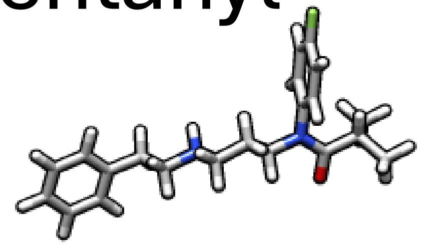

Structure 4
1.16 kcal/mol
5.56 %
1.85 Å
1.19 kcal/mol

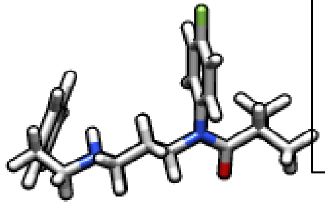

Structure 5
1.56 kcal/mol
2.89 %
1.57 Å

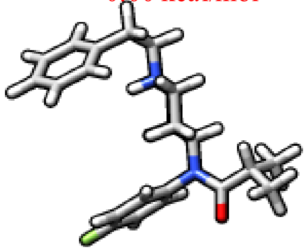

Structure 6
1.72 kcal/mol
2.25 %
1.35 Å

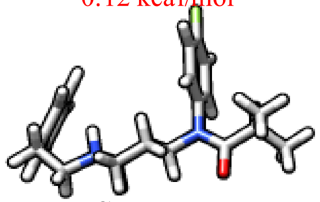

Structure 7
1.80 kcal/mol
1.99 %
0.65 Å

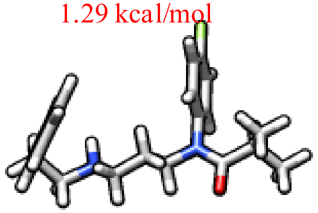

Structure 8
1.81 kcal/mol
1.95 %
1.44 Å

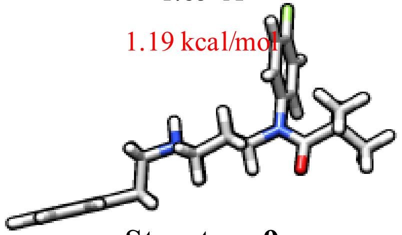

Structure 9
2.04 kcal/mol
1.33 %
2.00 Å

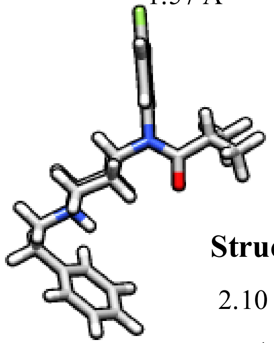

Structure 10
2.10 kcal/mol
1.21 %
1.87 Å

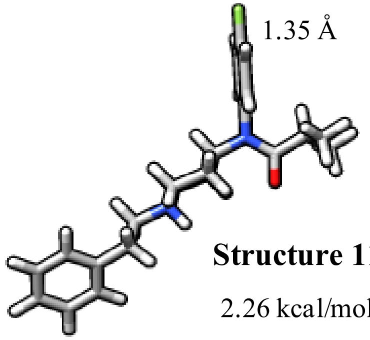

Structure 11
2.26 kcal/mol
0.94 %
2.09 Å

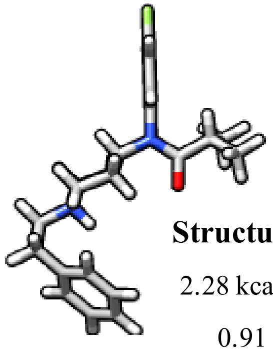

Structure 12
2.28 kcal/mol
0.91 %
1.94 Å

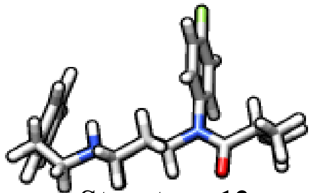

Structure 13
2.34 kcal/mol
0.83 %
0.82 Å

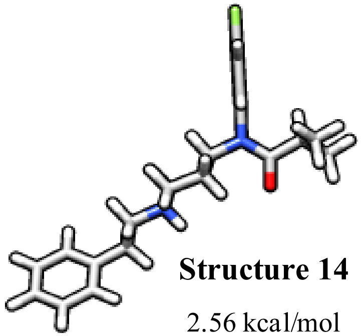

Structure 14
2.56 kcal/mol
0.58 %
2 13 Å

Figure S18. p-Chloro isobutyryl fentanyl

Structure #
ΔG (310.15K)
Boltzmann %
RMSD
ΔG (310.15K/haug)

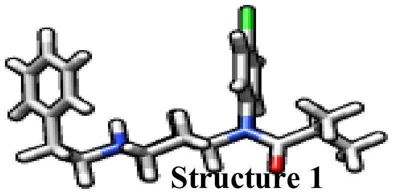

Structure 1
0.00 kcal/mol
31.88 %
0.00 Å
0.03 kcal/mol

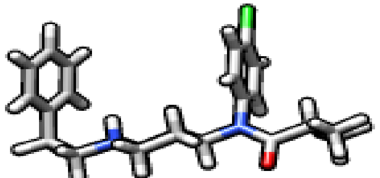

Structure 2
0.20 kcal/mol
22.89 %
0.92 Å
0.00 kcal/mol

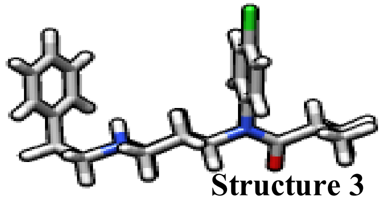

Structure 3
0.22 kcal/mol
22.15 %
0.46 Å
0.20 kcal/mol

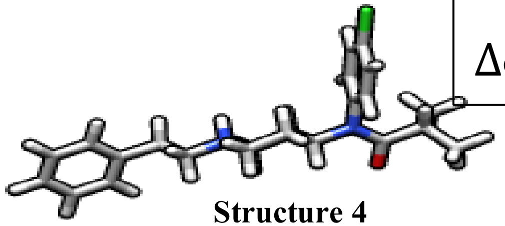

Structure 4
0.99 kcal/mol
6.35 %
1.60 Å
0.77 kcal/mol

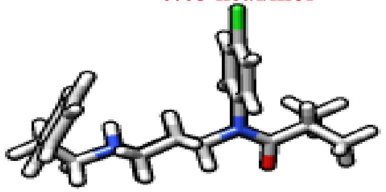

Structure 5
1.48 kcal/mol
2.87 %
1.09 Å

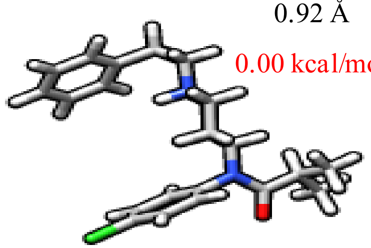

Structure 6
1.58 kcal/mol
2.45 %
1.13 Å

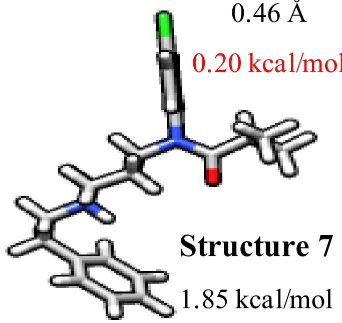

Structure 7
1.85 kcal/mol
1.58 %
2.03 Å

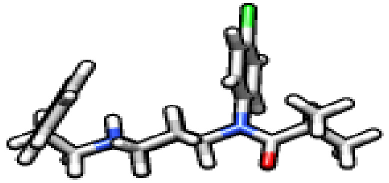

Structure 8
1.93 kcal/mol
1.39 %
1.02 Å

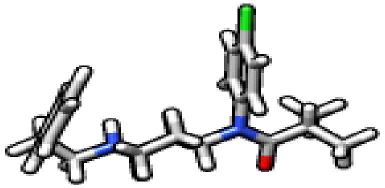

Structure 9
1.98 kcal/mol
1.29 %
1.10 Å

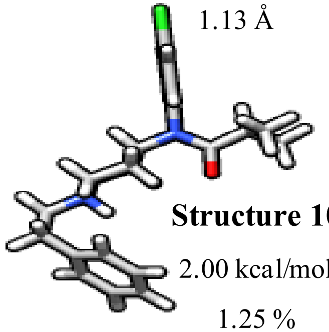

Structure 10
2.00 kcal/mol
1.25 %
2.06 Å

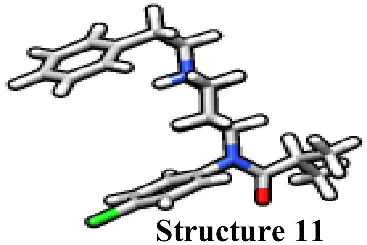

Structure 11
2.11 kcal/mol
1.04 %
1.36 Å

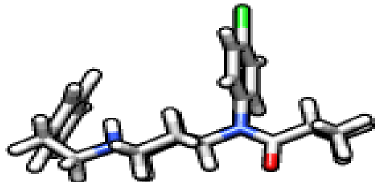

Structure 12
2.39 kcal/mol
0.66 %
0.97 Å

Figure S19. Pivaloyl fentanyl

Structure #
ΔG (310.15K)
Boltzmann %
RMSD
ΔG (310.15K/haug)

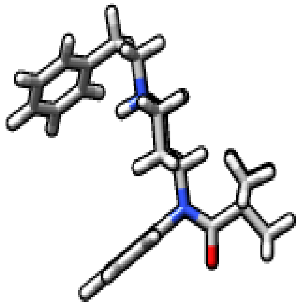

Structure 1
0.00 kcal/mol
29.06 %
0.00 Å
0.00 kcal/mol

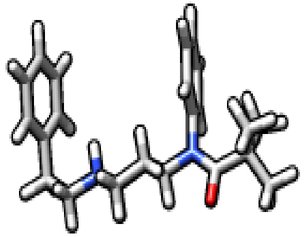

Structure 2
0.13 kcal/mol
23.65 %
1.38 Å
0.54 kcal/mol

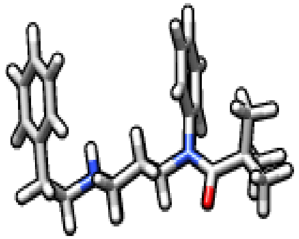

Structure 3
0.49 kcal/mol
13.20 %
1.32 Å
0.86 kcal/mol

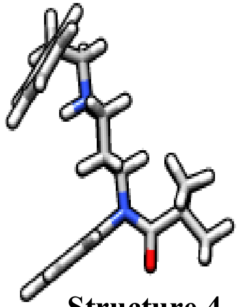

Structure 4
0.73 kcal/mol
8.94 %
1.00 Å
0.87 kcal/mol

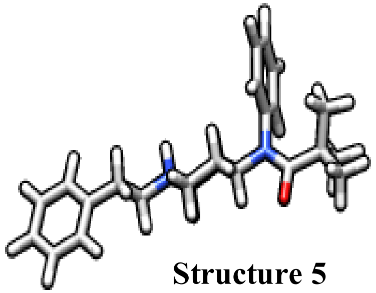

Structure 5
0.83 kcal/mol
7.55 %
1.96 Å
0.88 kcal/mol

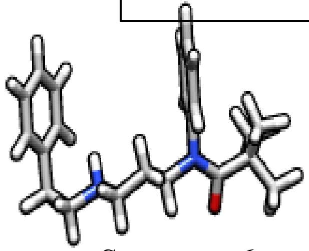

Structure 6
0.93 kcal/mol
6.44 %
1.28 Å
1.00 kcal/mol

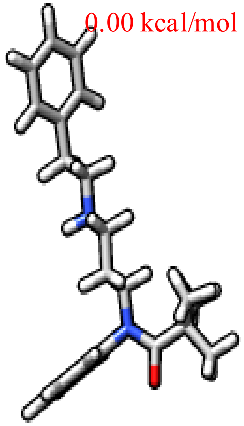

Structure 7
1.19 kcal/mol
4.23 %
1.55 Å

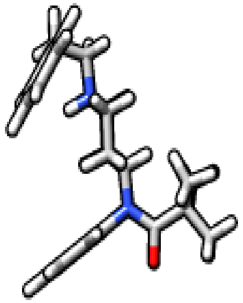

Structure 8
1.52 kcal/mol
2.49 %
1.00 Å

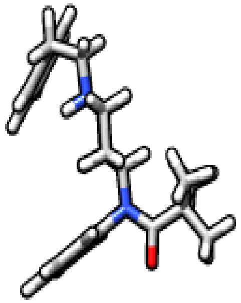

Structure 9
2.04 kcal/mol
1.07 %
0.94 Å

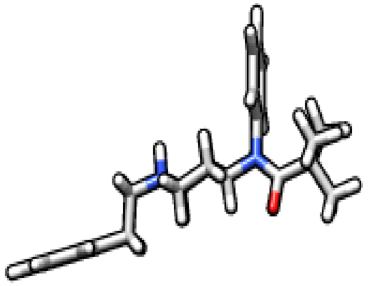

Structure 10
2.31 kcal/mol
0.69 %
2.12 Å

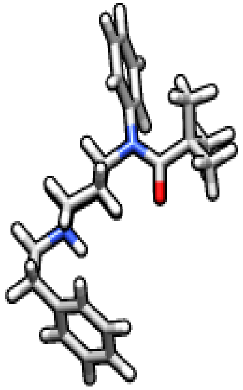

Structure 11
2.33 kcal/mol
0.67 %
1.57 Å

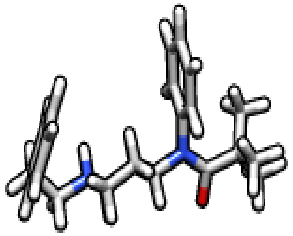

Structure 12
2.36 kcal/mol
0.63 %
1.51 Å

Figure S20. Butyryl fentanyl

Structure #
ΔG (310.15K)
Boltzmann %
RMSD

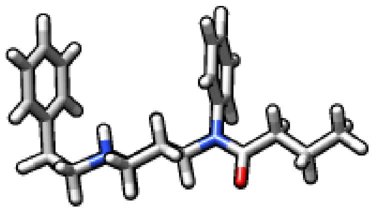

Structure 1
0.00 kcal/mol
59.65 %
0.00 Å
0.00 kcal/mol

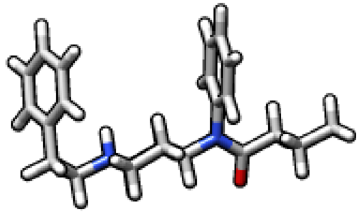

Structure 2
1.42 kcal/mol
5.96 %
0.51 Å
1.13 kcal/mol

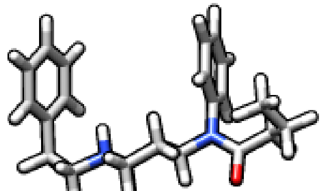

Structure 3
1.44 kcal/mol
5.80 %
0.84 Å
1.06 kcal/mol

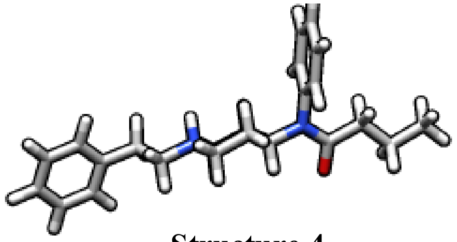

Structure 4
1.67 kcal/mol
4.00 %
1.82 Å

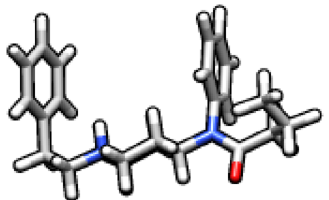

Structure 5
1.78 kcal/mol
3.33 %
0.83 Å

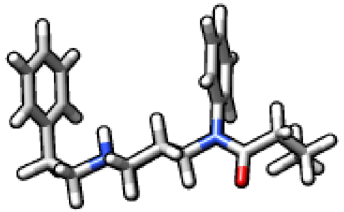

Structure 6
2.03 kcal/mol
2.23 %
0.80 Å

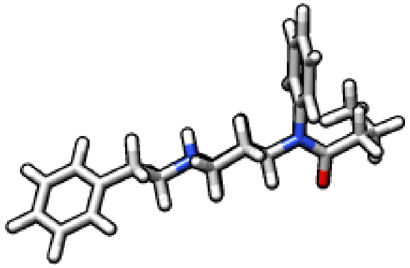

Structure 7
2.06 kcal/mol
2.11 %
1.70 Å

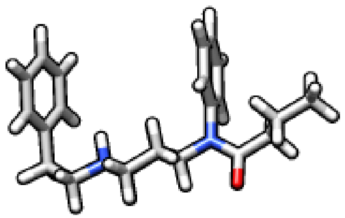

Structure 8
2.10 kcal/mol
1.98 %
0.48 Å

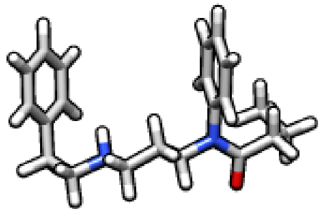

Structure 9
2.10 kcal/mol
1.98 %
1.28 Å

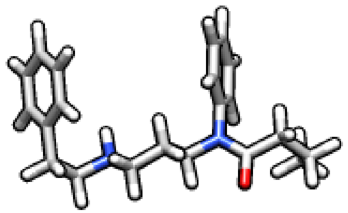

Structure 10
2.24 kcal/mol
1.57 %
0.98 Å

Figure S20. Butyryl fentanyl

Structure #
ΔG (310.15K)
Boltzmann %
RMSD
ΔG (310.15K/haug)

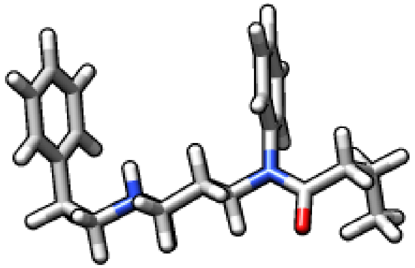

Structure 11
2.52 kcal/mol
1.00 %
0.79 Å

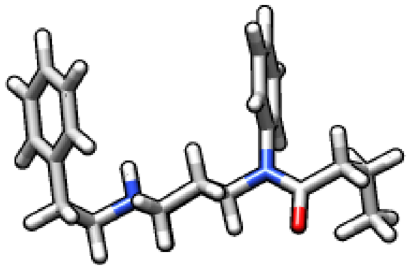

Structure 12
2.57 kcal/mol
0.93 %
0.98 Å

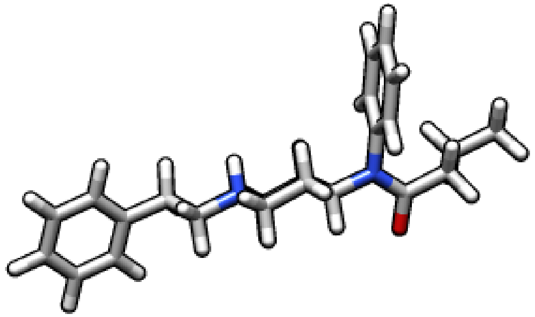

Structure 13
2.60 kcal/mol
0.88 %
1.83 Å

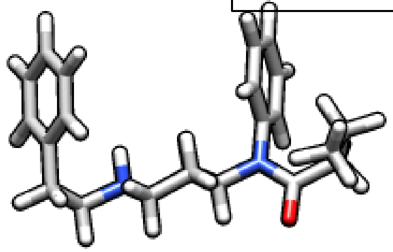

Structure 14
2.62 kcal/mol
0.85 %
1.35 Å

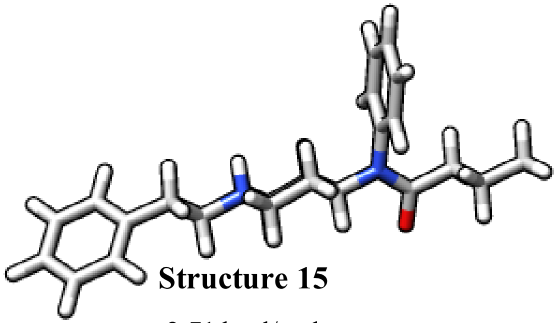

Structure 15
2.71 kcal/mol
0.73 %
1.70 Å

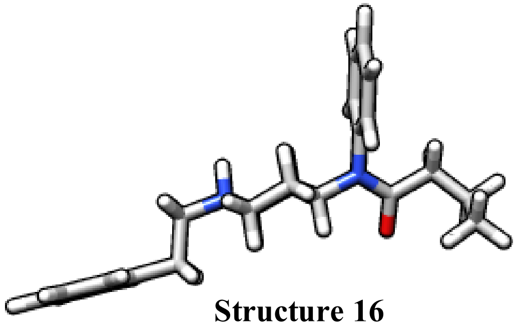

Structure 16
2.84 kcal/mol
0.60 %
2.08 Å

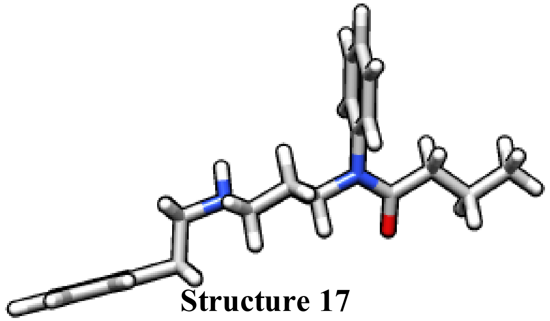

Structure 17
2.86 kcal/mol
0.58 %
1.92 Å

Figure S21. o-Fluoro butyryl fentanyl

Structure #
ΔG (310.15K)
Boltzmann %
RMSD
ΔG (310.15K/haug)

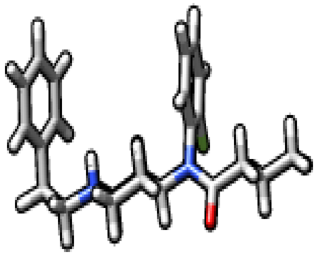

Structure 1
0.00 kcal/mol
12.74 %
0.00 Å
0.00 kcal/mol

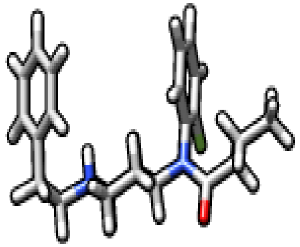

Structure 2
0.03 kcal/mol
12.09 %
1.26 Å
0.33 kcal/mol

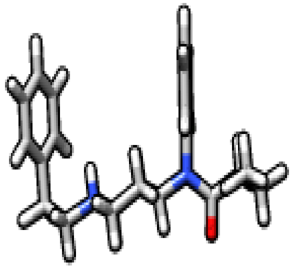

Structure 3
0.28 kcal/mol
8.15 %
0.77 Å
0.21 kcal/mol

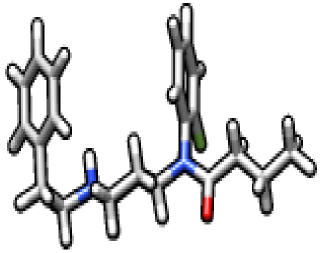

Structure 4
0.41 kcal/mol
6.59 %
1.05 Å
0.46 kcal/mol

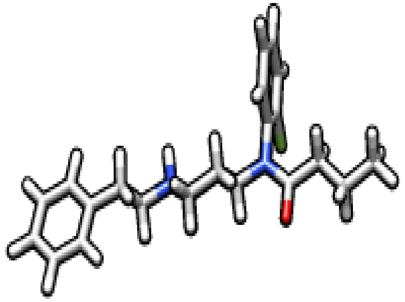

Structure 5
0.61 kcal/mol
4.75 %
1.88 Å

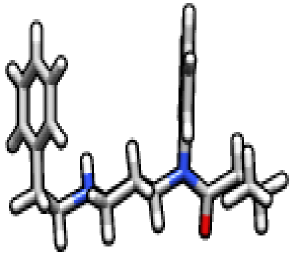

Structure 6
0.79 kcal/mol
3.55 %
1.06 Å

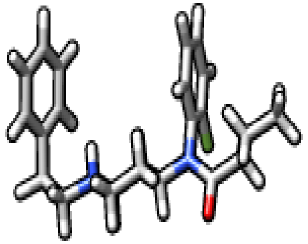

Structure 7
0.81 kcal/mol
3.41 %
0.77 Å

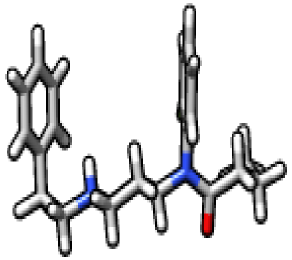

Structure 8
0.97 kcal/mol
2.64 %
1.01 Å

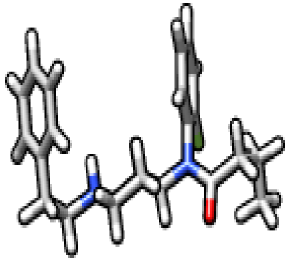

Structure 9
1.02 kcal/mol
2.43 %
0.92 Å

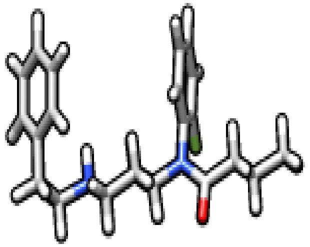

Structure 10
1.03 kcal/mol
2.41 %
0.83 Å

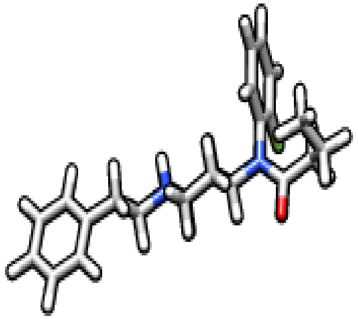

Structure 11
1.08 kcal/mol
2.23 %
1.98 Å

Figure S21. o-Fluoro butyryl fentanyl

Structure #
ΔG (310.15K)
Boltzmann %
RMSD
ΔG (310.15K/haug)

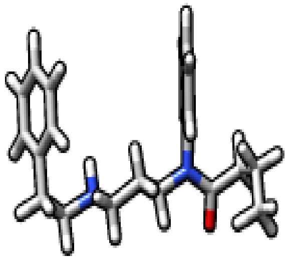

Structure 12
1.09 kcal/mol
2.19 %
1.05 Å

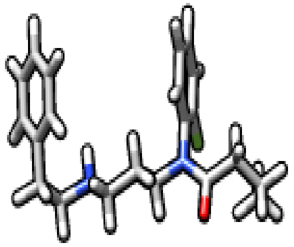

Structure 13
1.13 kcal/mol
2.03 %
0.97 Å

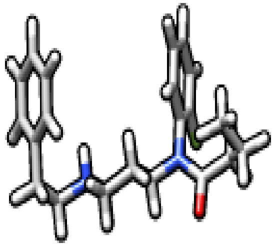

Structure 14
1.15 kcal/mol
1.98 %
1.45 Å

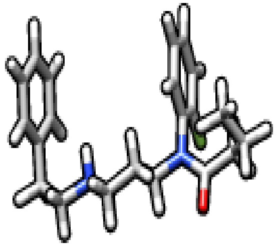

Structure 15
1.16 kcal/mol
1.95 %
0.93 Å

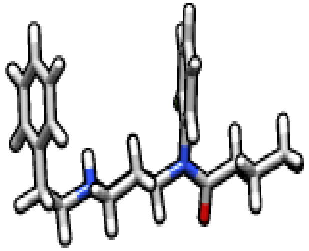

Structure 16
1.18 kcal/mol
1.88 %
0.86 Å

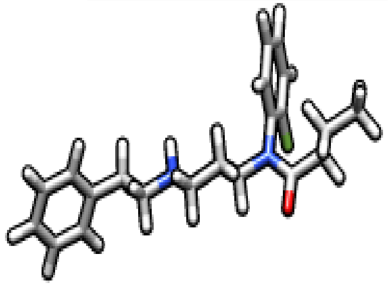

Structure 17
1.26 kcal/mol
1.65 %
1.71 Å

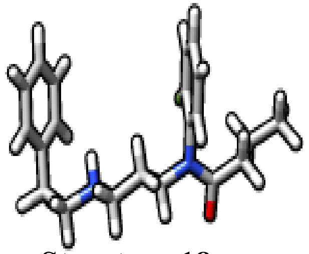

Structure 18
1.26 kcal/mol
1.64 %
1.07 Å

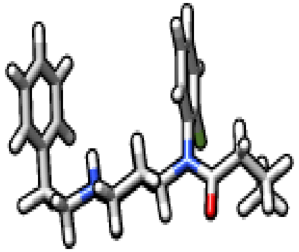

Structure 19
1.28 kcal/mol
1.61 %
0.55 Å

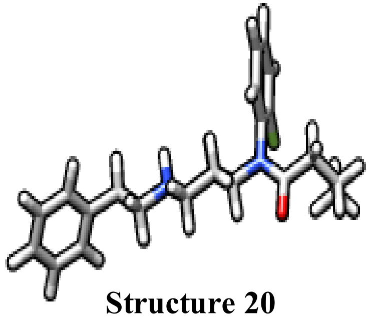

Structure 20
1.29 kcal/mol
1.57 %
1.71 Å

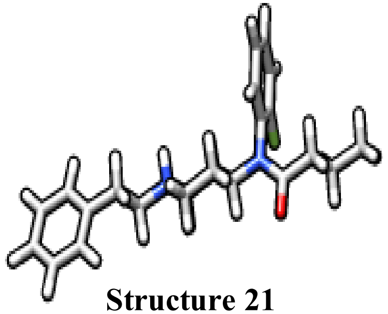

Structure 21
1.33 kcal/mol
1.49 %
1.81 Å

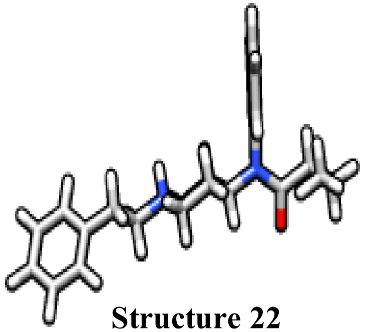

Structure 22
1.33 kcal/mol
1.47 %
1.94 Å

Figure S21. o-Fluoro butyryl fentanyl

Structure #
ΔG (310.15K)
Boltzmann %
RMSD
ΔG (310.15K/haug)

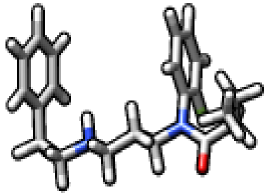

Structure 23
1.52 kcal/mol
1.09 %
1.06 Å

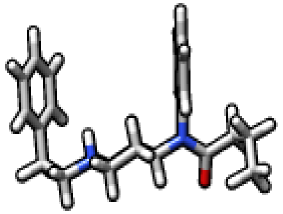

Structure 24
1.13 kcal/mol
2.03 %
0.97 Å

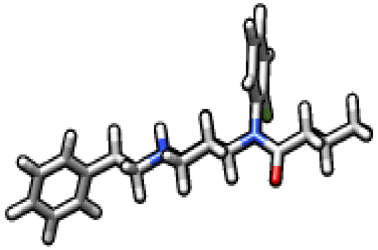

Structure 25
1.52 kcal/mol
1.08 %
0.91 Å

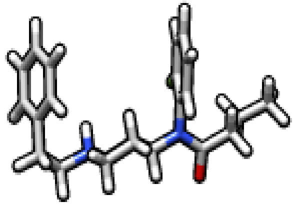

Structure 26
1.70 kcal/mol
0.82 %
0.93 Å

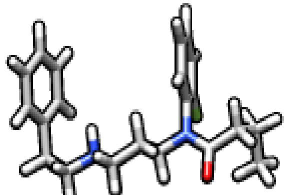

Structure 27
1.84 kcal/mol
0.65 %
0.52 Å

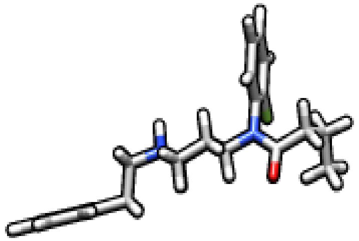

Structure 28
1.86 kcal/mol
0.63 %
1.98 Å

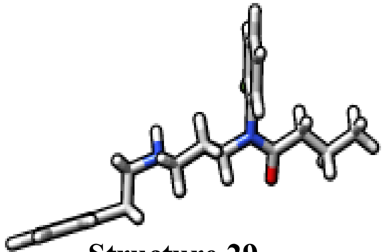

Structure 29
1.87 kcal/mol
0.61 %
2.07 Å

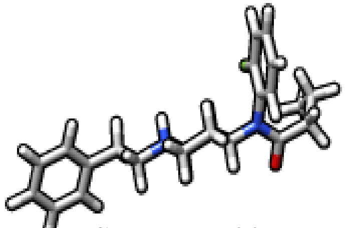

Structure 30
1.91 kcal/mol
0.58 %
2.05 Å

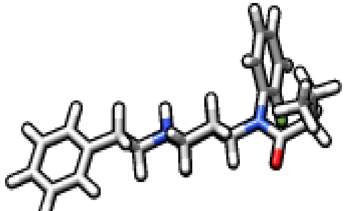

Structure 31
1.94 kcal/mol
0.55 %
2.09 Å

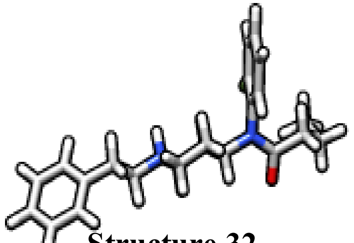

Structure 32
1.96 kcal/mol
0.53 %
1.95 Å

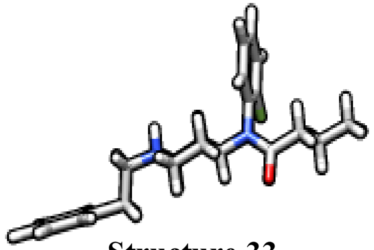

Structure 33
1.99 kcal/mol
0.51 %
1.93 Å

Figure S22. Methoxyacetyl fentanyl

Structure #
ΔG (310.15K)
Boltzmann %
RMSD
ΔG (310.15K/haug)

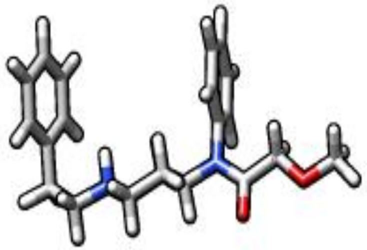

Structure 1
0.00 kcal/mol
53.08 %
0.00 Å
0.00 kcal/mol

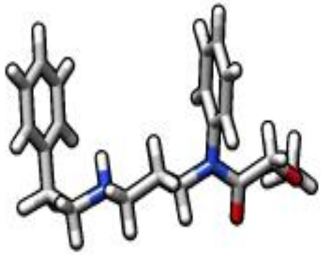

Structure 2
1.01 kcal/mol
10.38 %
1.14 Å
0.88 kcal/mol

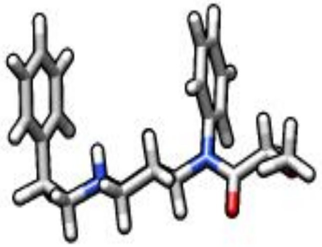

Structure 3
1.35 kcal/mol
5.98 %
0.67 Å
1.32 kcal/mol

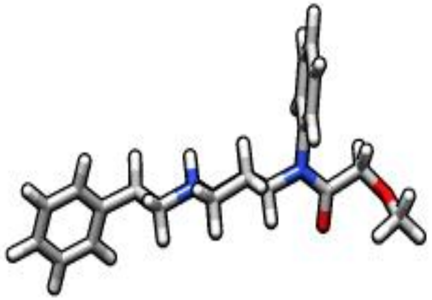

Structure 4
1.36 kcal/mol
5.90 %
1.99 Å
0.99 kcal/mol

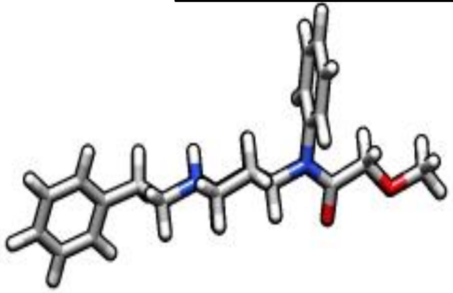

Structure 5
1.36 kcal/mol
5.88 %
1.84 Å
1.11 kcal/mol

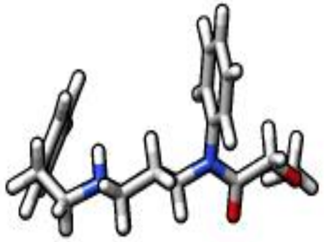

Structure 6
1.97 kcal/mol
2.17 %
1.68 Å

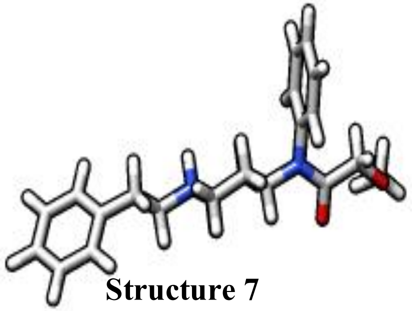

Structure 7
1.99 kcal/mol
2.12 %
1.74 Å

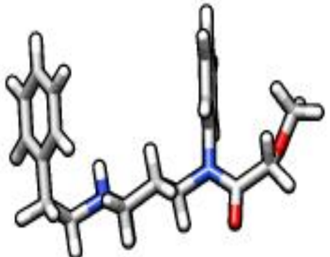

Structure 8
1.99 kcal/mol
2.10 %
0.98 Å

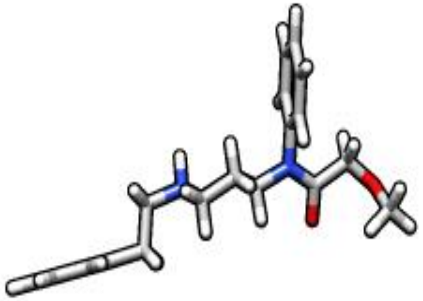

Structure 9
2.16 kcal/mol
1.60 %
2.06 Å

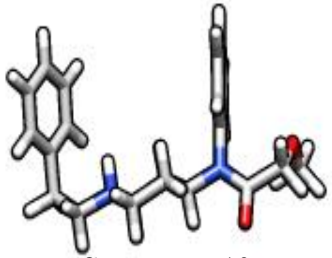

Structure 10
2.20 kcal/mol
1.50 %
0.81 Å

Figure S22. Methoxyacetyl fentanyl

Structure #
ΔG (310.15K)
Boltzmann %
RMSD
ΔG (310.15K/haug)

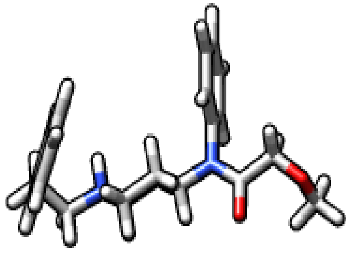

Structure 11
2.29 kcal/mol
1.30 %
1.33 Å

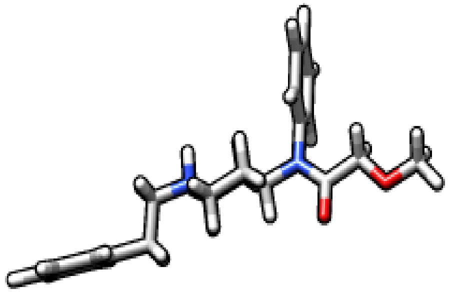

Structure 12
2.44 kcal/mol
1.01 %
2.00 Å

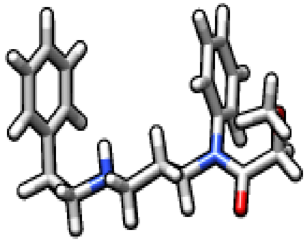

Structure 13
2.58 kcal/mol
0.81 %
0.84 Å

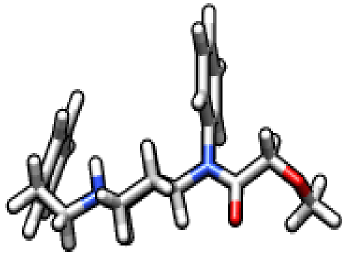

Structure 14
2.59 kcal/mol
0.80 %
1.03 Å

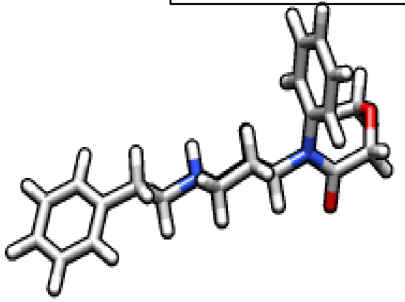

Structure 15
2.63 kcal/mol
0.75 %
1.74 Å

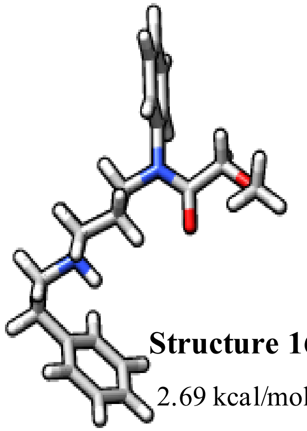

Structure 16
2.69 kcal/mol
0.67 %
1.78 Å

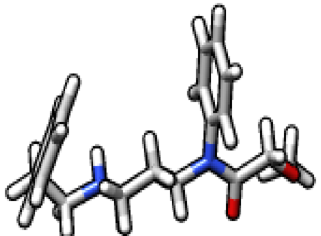

Structure 17
2.71 kcal/mol
0.65 %
0.90 Å

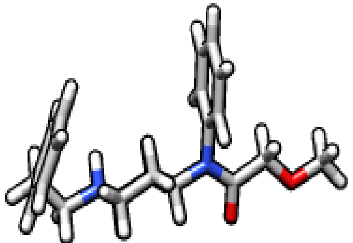

Structure 18
2.78 kcal/mol
0.58 %
0.72 Å

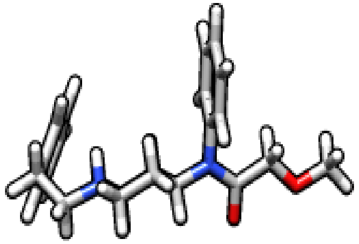

Structure 19
2.83 kcal/mol
0.54 %
1.50 Å

Figure S23. o-Fluoro fentanyl

Structure #
ΔG (310.15K)
Boltzmann %
RMSD
ΔG (310.15K/haug)

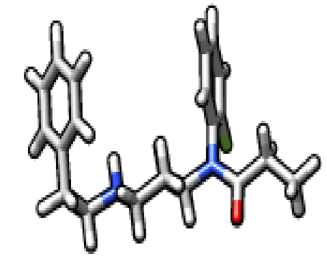

Structure 1
0.00 kcal/mol
27.28 %
0.00 Å
0.00 kcal/mol

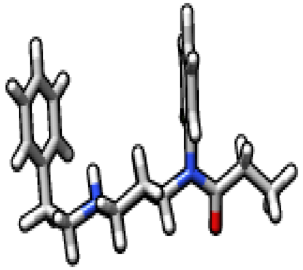

Structure 2
0.54 kcal/mol
11.40 %
0.88 Å
0.35 kcal/mol

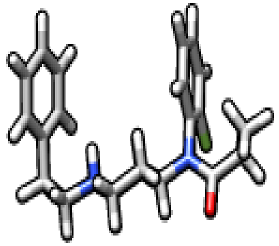

Structure 3
0.68 kcal/mol
9.11 %
0.87 Å
0.76 kcal/mol

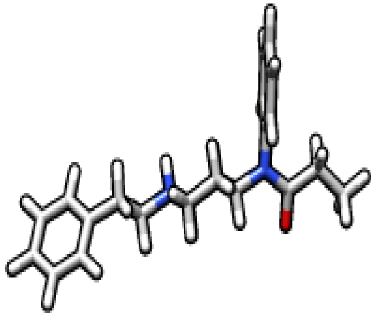

Structure 4
0.82 kcal/mol
7.22 %
1.84 Å
0.55 kcal/mol

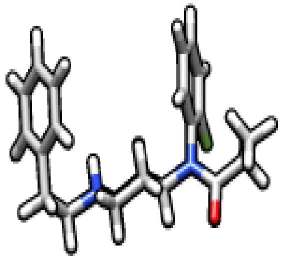

Structure 5
0.84 kcal/mol
6.94 %
0.41 Å
0.83 kcal/mol

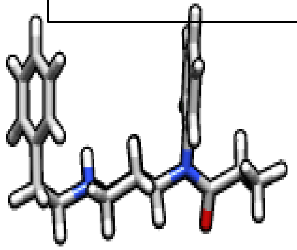

Structure 6
1.10 kcal/mol
4.55 %
0.79 Å

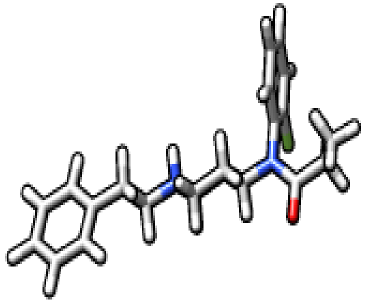

Structure 7
1.21 kcal/mol
3.86 %
1.60 Å

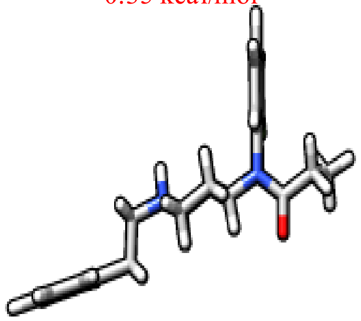

Structure 8
1.23 kcal/mol
3.70 %
2.07 Å

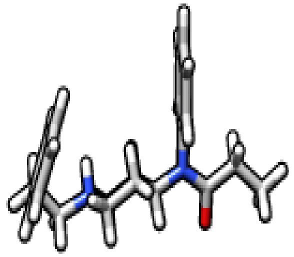

Structure 9
1.47 kcal/mol
2.51 %
0.99 Å

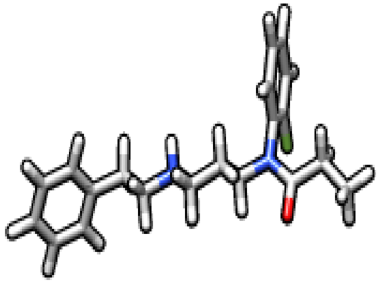

Structure 10
1.48 kcal/mol
2.49 %
1.88 Å

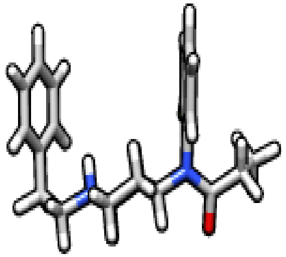

Structure 11
1.60 kcal/mol
2.04 %
0.93 Å

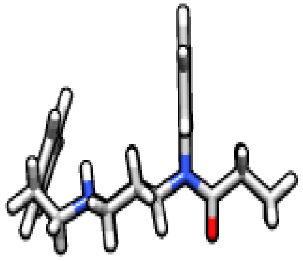

Structure 12
1.65 kcal/mol
1.89 %
1.32 Å

Figure S23. o-Fluoro fentanyl

Structure #
ΔG (310.15K)
Boltzmann %
RMSD
ΔG (310.15K/haug)

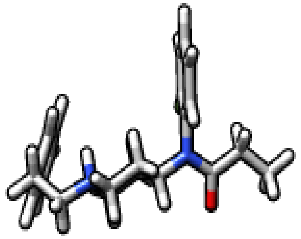

Structure 13
1.75 kcal/mol
1.59 %
1.37 Å

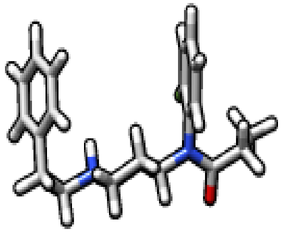

Structure 14
1.78 kcal/mol
1.53 %
1.16 Å

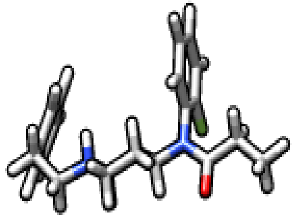

Structure 15
1.78 kcal/mol
1.53 %
1.29 Å

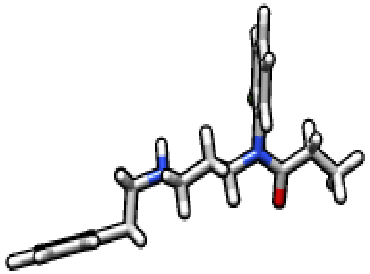

Structure 16
1.83 kcal/mol
1.41 %
2.05 Å

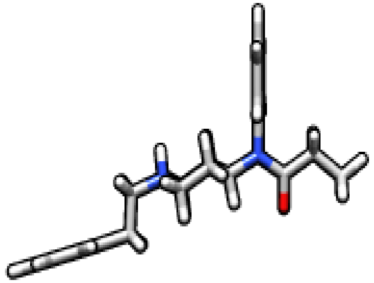

Structure1 17
1.89 kcal/mol
1.27 %
2.06 Å

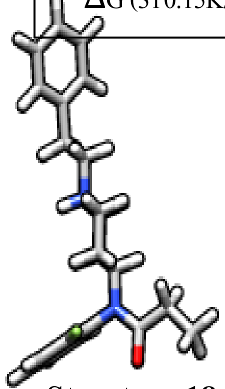

Structure 18
1.93 kcal/mol
1.20 %
2.02 Å

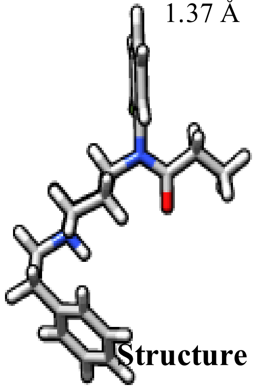

Structure 19
2.06 kcal/mol
0.97 %
1.39 Å

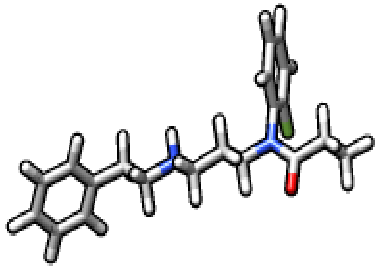

Structure 20
2.09 kcal/mol
0.92 %
1.89 Å

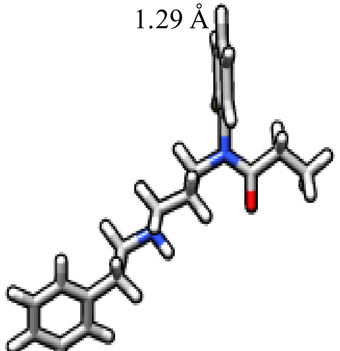

Structure 21
2.10 kcal/mol
0.90 %
2.07 Å

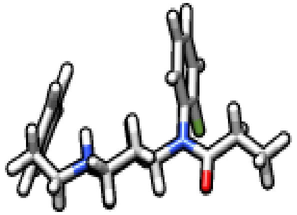

Structure 22
2.18 kcal/mol
0.80 %
1.28 Å

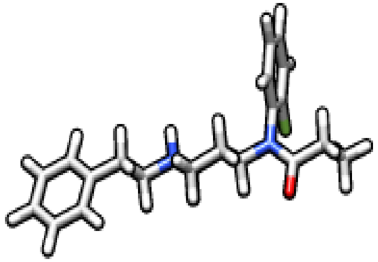

Structure 23
2.21 kcal/mol
0.76 %
1.65 Å

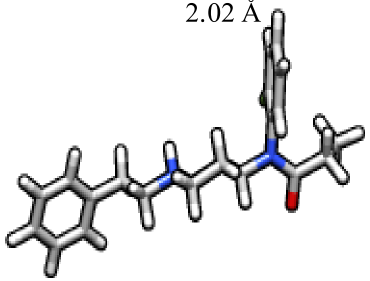

Structure 24
2.26 kcal/mol
0.69 %
1.84 Å

Figure S24. m-Fluoro fentanyl

Structure #
ΔG (310.15K)
Boltzmann %
RMSD
ΔG (310.15K/haug)

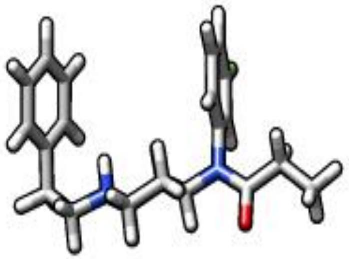

Structure 1
0.00 kcal/mol
23.34 %
0.00 Å
0.00 kcal/mol

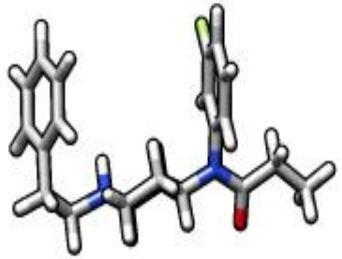

Structure 2
0.41 kcal/mol
11.97 %
0.87 Å
0.35 kcal/mol

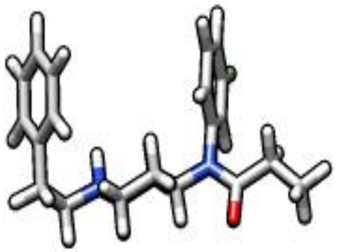

Structure 3
0.54 kcal/mol
9.68 %
0.85 Å
0.60 kcal/mol

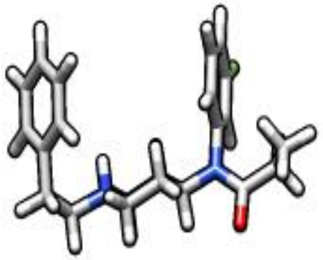

Structure 4
0.58 kcal/mol
9.16 %
0.99 Å
0.57 kcal/mol

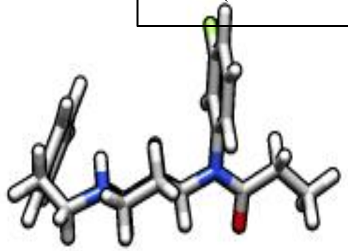

Structure 5
0.64 kcal/mol
8.33 %
1.54 Å
0.94 kcal/mol

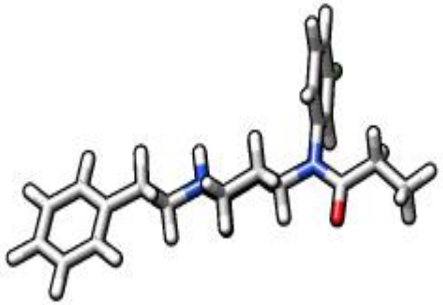

Structure 6
1.07 kcal/mol
4.14 %
1.92 Å

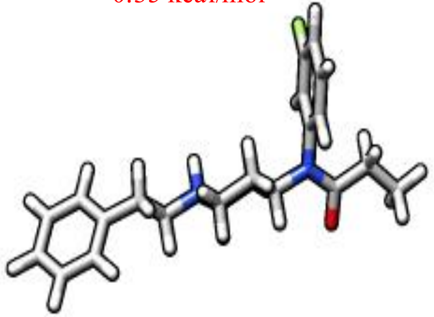

Structure 7
1.25 kcal/mol
3.10 %
1.87 Å

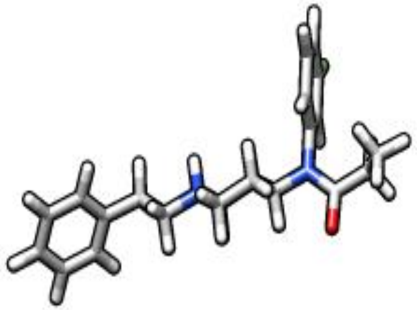

Structure 8
1.61 kcal/mol
1.71 %
1.66 Å

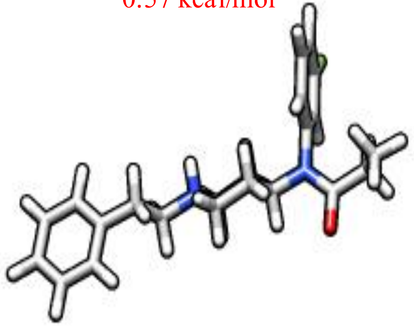

Structure 9
1.63 kcal/mol
1.67 %
1.87 Å

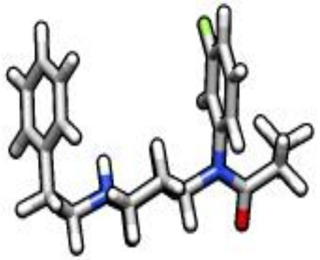

Structure 10
1.67 kcal/mol
1.56 %
0.97 Å

Figure S24. m-Fluoro fentanyl

Structure #
ΔG (310.15K)
Boltzmann %
RMSD
ΔG (310.15K/haug)

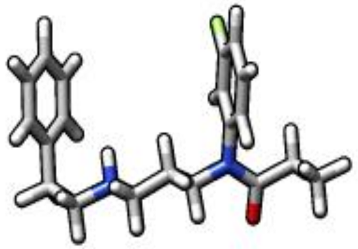

Structure 11
1.68 kcal/mol
1.53 %
1.10 Å

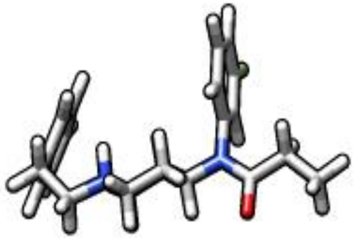

Structure 12
1.70 kcal/mol
1.49 %
1.05 Å

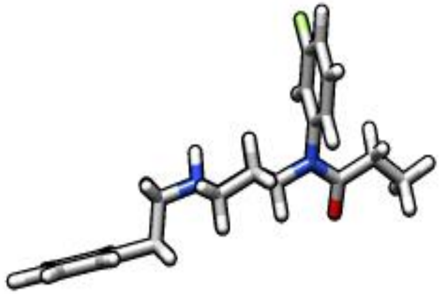

Structure 13
1.72 kcal/mol
1.44 %
2.08 Å

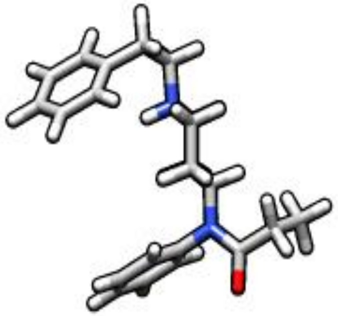

Structure 14
1.73 kcal/mol
1.42 %
1.35 Å

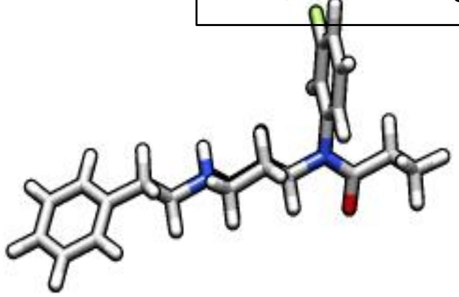

Structure 15
1.73 kcal/mol
1.42 %
1.92 Å

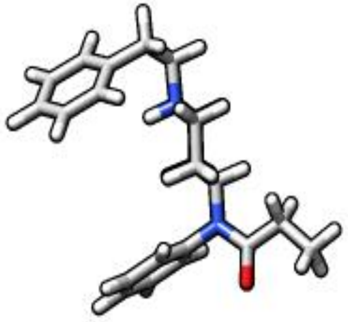

Structure 16
1.75 kcal/mol
1.38 %
1.28 Å

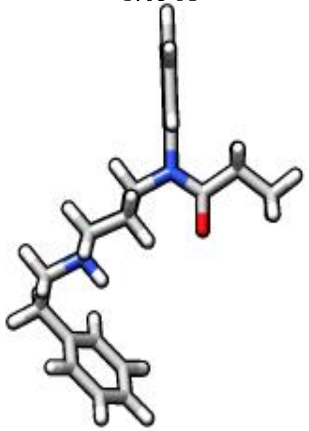

Structure 17
1.80 kcal/mol
1.27 %
1.72 Å

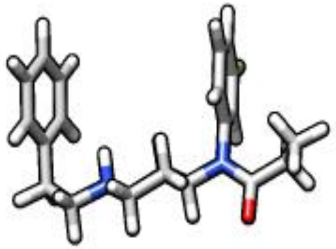

Structure 18
1.82 kcal/mol
1.22 %
0.38 Å

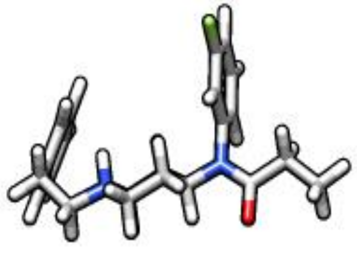

Structure 19
1.90 kcal/mol
1.07 %
0.76 Å

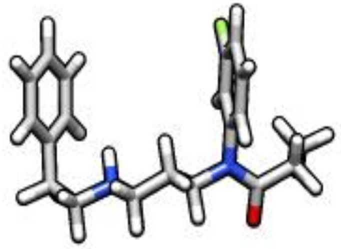

Structure 20
1.94 kcal/mol
1.00 %
1.29 Å

Figure S24. m-Fluoro fentanyl

Structure #

ΔG (310.15K)

Boltzmann %

RMSD

ΔG (310.15K/haug)

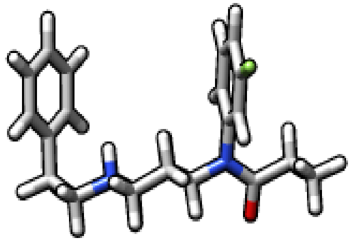

Structure 21

2.02 kcal/mol

0.88 %

0.84 Å

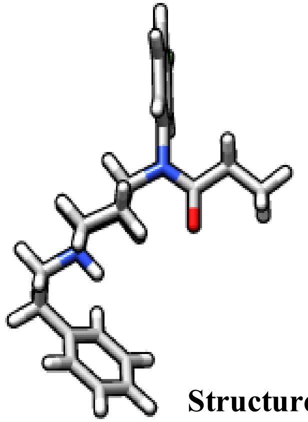

Structure 22

2.02 kcal/mol

0.88 %

1.70 Å

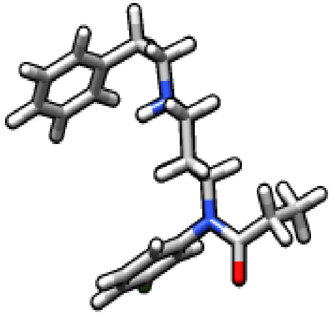

Structure 23

2.06 kcal/mol

0.83 %

1.13 Å

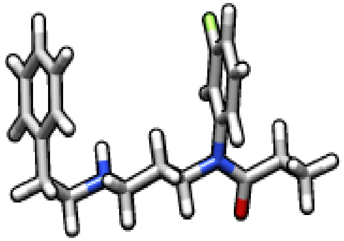

Structure 24

2.14 kcal/mol

0.73 %

0.95 Å

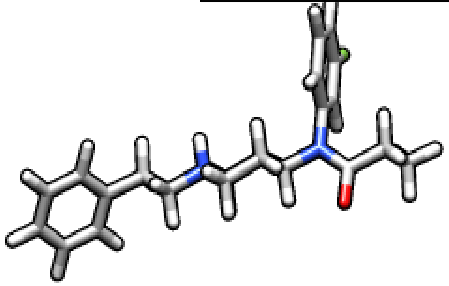

Structure 25

2.15 kcal/mol

0.71 %

1.71 Å

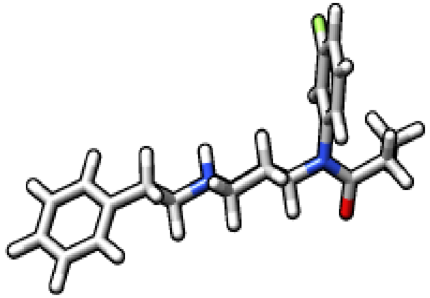

Structure 26

2.16 kcal/mol

0.70 %

1.87 Å

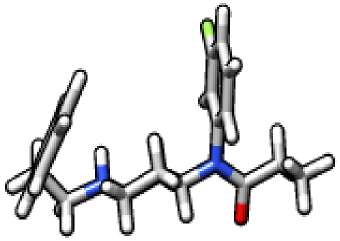

Structure 27

2.17 kcal/mol

0.69 %

0.89 Å

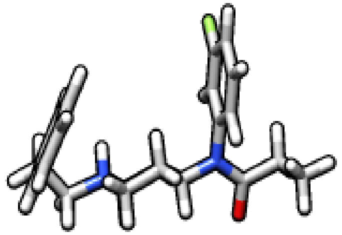

Structure 28

2.23 kcal/mol

0.62 %

0.84 Å

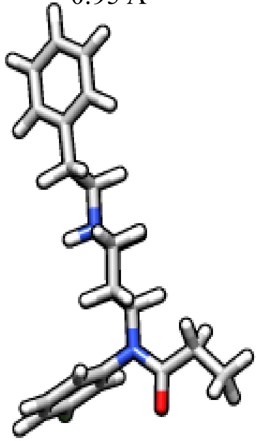

Structure 29

2.25 kcal/mol

0.61 %

2.20 Å

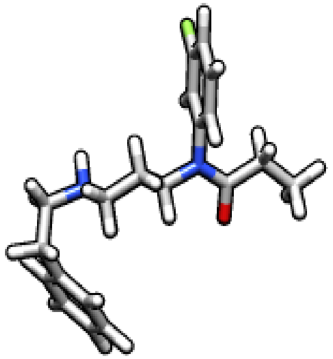

Structure 30

2.35 kcal/mol

0.51 %

1.95 Å

Figure S25. p-Fluoro fentanyl

Structure #
ΔG (310.15K)
Boltzmann %
RMSD
ΔG (310.15K/haug)

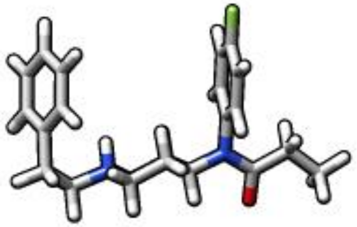

Structure 1
0.00 kcal/mol
60.93 %
0.00 Å
0.00 kcal/mol

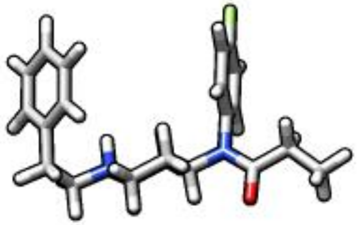

Structure 2
1.35 kcal/mol
6.87 %
0.82 Å
1.38 kcal/mol

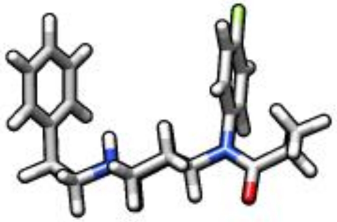

Structure 3
1.72 kcal/mol
3.73 %
0.41 Å

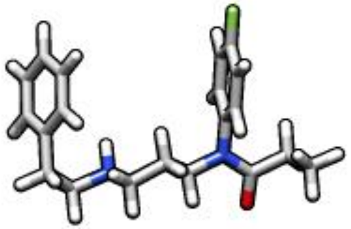

Structure 4
1.78 kcal/mol
3.42 %
0.40 Å

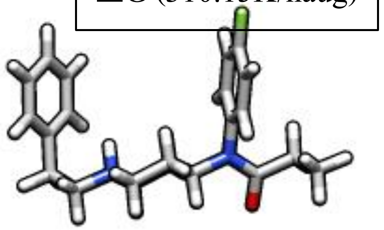

Structure 5
1.83 kcal/mol
3.12 %
0.83 Å

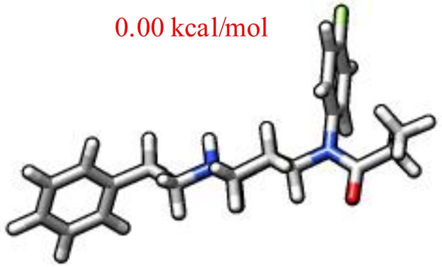

Structure 6
2.04 kcal/mol
2.24 %
1.64 Å

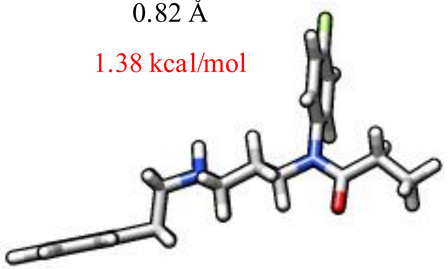

Structure 7
2.30 kcal/mol
1.47 %
2.05 Å

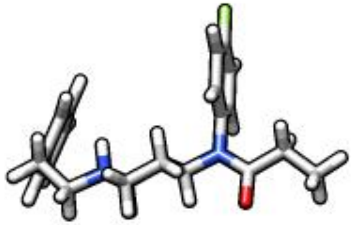

Structure 8
2.45 kcal/mol
1.15 %
0.79 Å

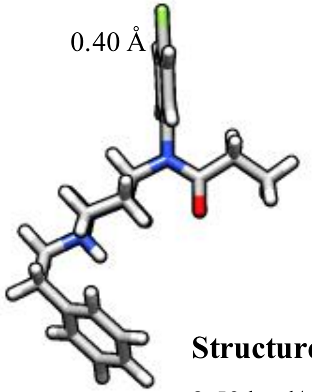

Structure 9
2.59 kcal/mol
0.92 %
2.02Å

Figure S25. p-Fluoro fentanyl

Structure #
ΔG (310.15K)
Boltzmann %
RMSD
ΔG (310.15K/haug)

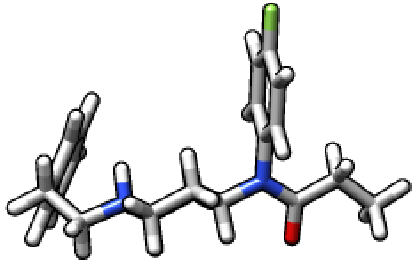

Structure 10
2.59 kcal/mol
0.92 %
1.48 Å

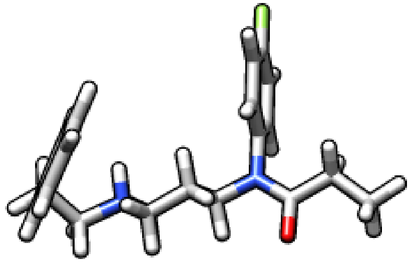

Structure 11
2.59 kcal/mol
0.92 %
1.26 Å

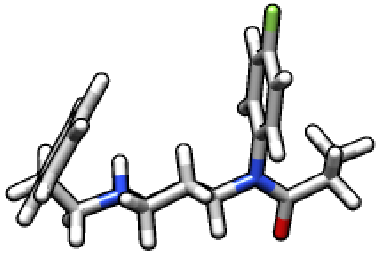

Structure 12
2.76 kcal/mol
0.70 %
0.77 Å

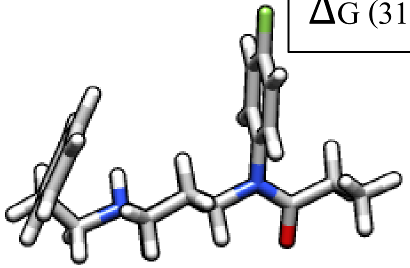

Structure 13
2.78 kcal/mol
0.67 %
0.90 Å

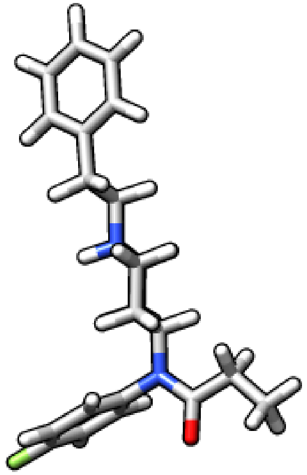

Structure 14
2.82 kcal/mol
0.63 %
2.18 Å

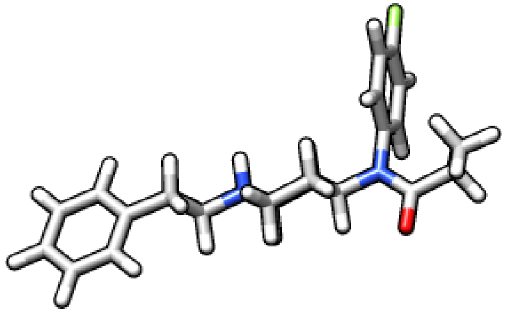

Structure 15
2.84 kcal/mol
0.61 %
1.88 Å

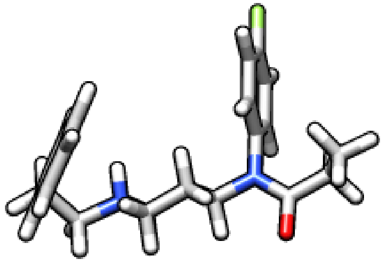

Structure 16
2.87 kcal/mol
0.58 %
1.54 Å

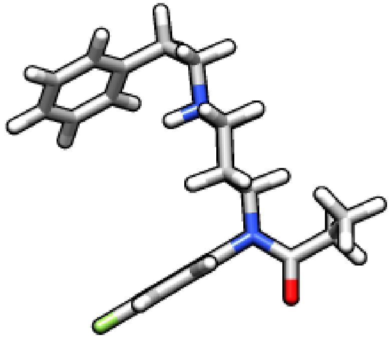

Structure 17
2.87 kcal/mol
0.58 %
1.17 Å

Figure S26. o-Methyl fentanyl

Structure #
ΔG (310.15K)
Boltzmann %
RMSD
ΔG (310.15K/haug)

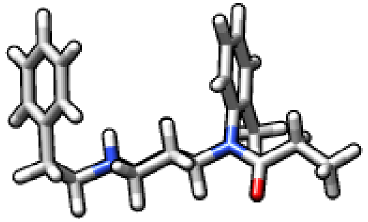

Structure 1
0.00 kcal/mol
34.95 %
0.00 Å
0.00 kcal/mol

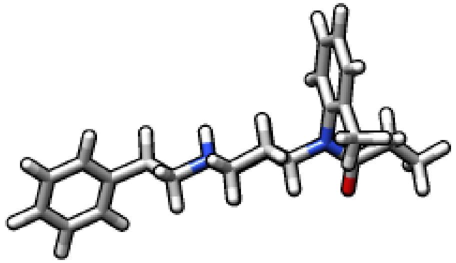

Structure 2
0.46 kcal/mol
16.71 %
1.59 Å
0.33 kcal/mol

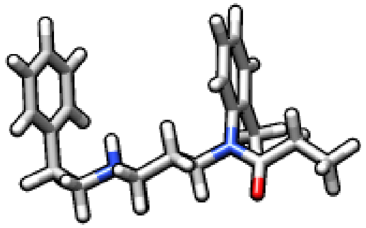

Structure 3
0.62 kcal/mol
12.76 %
0.80 Å
0.35 kcal/mol

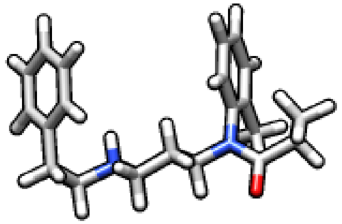

Structure 4
1.24 kcal/mol
4.68 %
0.40 Å

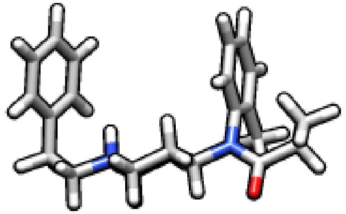

Structure 5
1.40 kcal/mol
3.62 %
0.85 Å

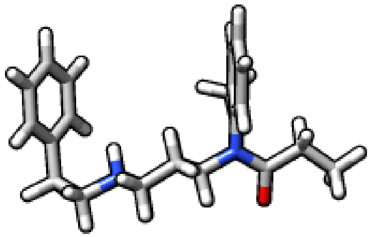

Structure 6
1.55 kcal/mol
2.81 %
0.93 Å

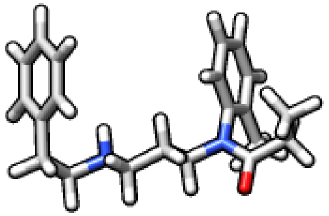

Structure 7
1.61 kcal/mol
2.58 %
0.61 Å

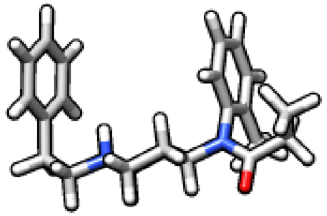

Structure 8
1.73 kcal/mol
2.11 %
0.82 Å

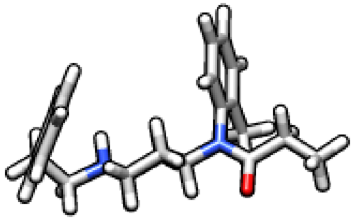

Structure 9
1.74 kcal/mol
2.08 %
1.42 Å

Figure S26. o-Methyl fentanyl

Structure #
ΔG (310.15K)
Boltzmann %
RMSD
ΔG (310.15K/haug)

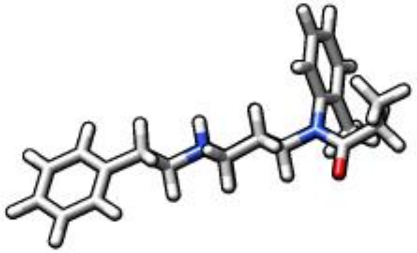

Structure 10
1.82 kcal/mol
1.83 %
1.64 Å

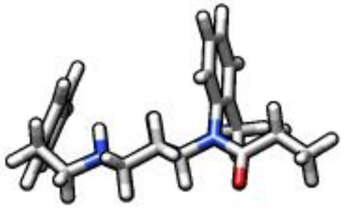

Structure 11
1.88 kcal/mol
1.66 %
1.19 Å

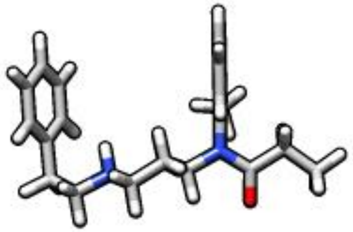

Structure 12
2.05 kcal/mol
1.25 %
1.04 Å

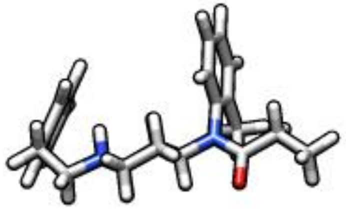

Structure 13
2.07 kcal/mol
1.21 %
1.21 Å

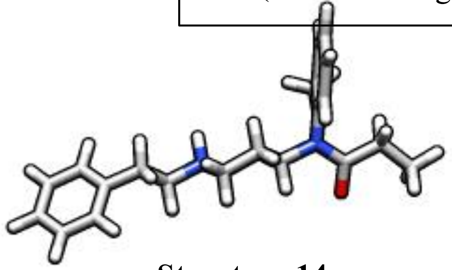

Structure 14
2.10 kcal/mol
1.16 %
1.88 Å

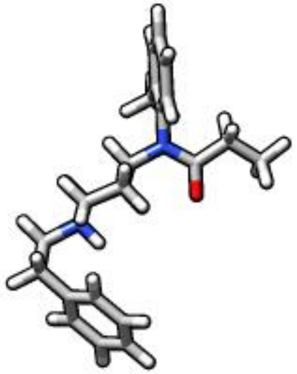

Structure 15
2.12 kcal/mol
1.13 %
1.37 Å

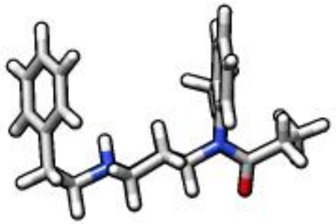

Structure 16
2.22 kcal/mol
0.96 %
1.10 Å

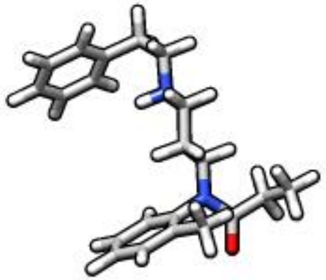

Structure 17
2.23 kcal/mol
0.94 %
1.06 Å

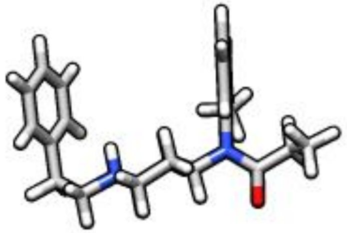

Structure 18
2.25 kcal/mol
0.91 %
1.06 Å

Figure S27. m-Methyl fentanyl

Structure #
ΔG (310.15K)
Boltzmann %
RMSD
ΔG (310.15K/haug)

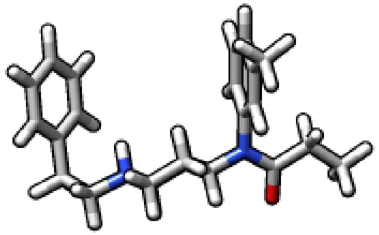

Structure 1
0.00 kcal/mol
34.10 %
0.00 Å
0.13 kcal/mol

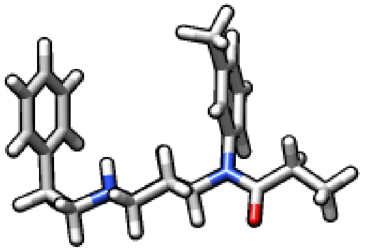

Structure 2
0.15 kcal/mol
26.73 %
1.21 Å
0.00 kcal/mol

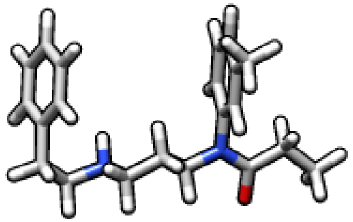

Structure 3
0.98 kcal/mol
7.01 %
0.82 Å
0.55 kcal/mol

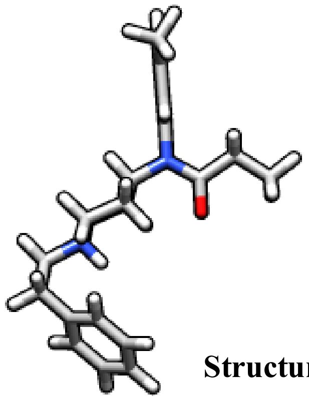

Structure 4
1.36 kcal/mol
3.73 %
1.60 Å

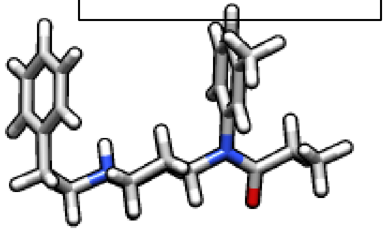

Structure 5
1.39 kcal/mol
3.59 %
1.00 Å

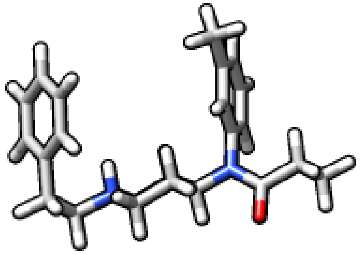

Structure 6
1.48 kcal/mol
3.11 %
0.91 Å

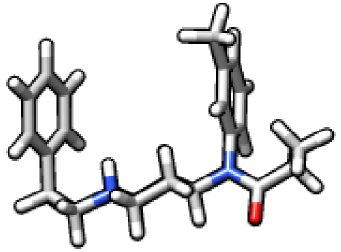

Structure 7
1.59 kcal/mol
2.60 %
1.17 Å

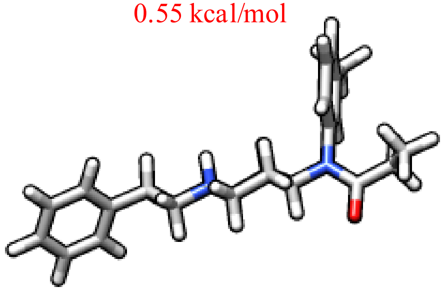

Structure 8
1.66 kcal/mol
2.33 %
1.78 Å

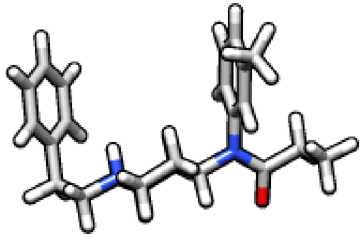

Structure 9
1.69 kcal/mol
2.19 %
0.42 Å

Figure S27. m-Methyl fentanyl

Structure #
ΔG (310.15K)
Boltzmann %
RMSD
ΔG (310.15K/haug)

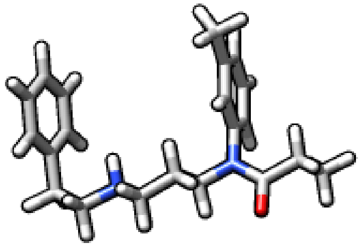

Structure 10
1.85 kcal/mol
1.69 %
1.12 Å

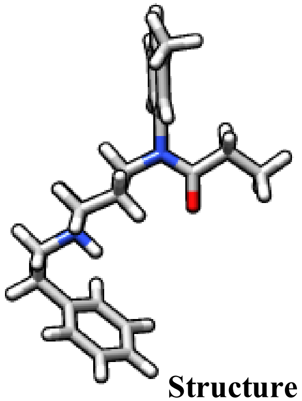

Structure 11
2.08 kcal/mol
1.17 %
1.75 Å

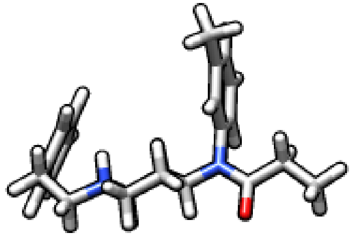

Structure 12
2.09 kcal/mol
1.15 %
1.01 Å

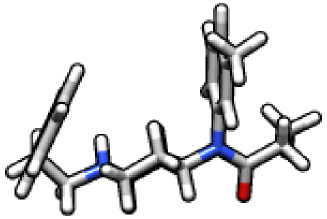

Structure 13
2.11 kcal/mol
1.11 %
1.30 Å

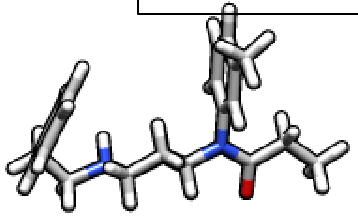

Structure 14
2.16 kcal/mol
1.04 %
1.21 Å

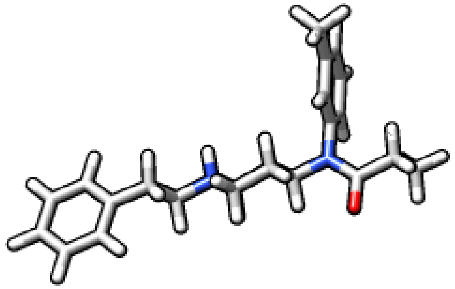

Structure 15
2.25 kcal/mol
0.89 %
1.77 Å

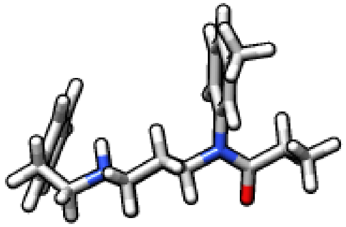

Structure 16
2.35 kcal/mol
0.76 %
1.14 Å

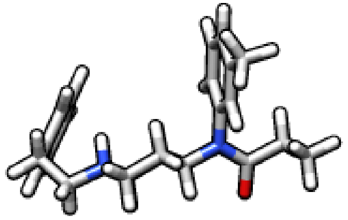

Structure 17
2.38 kcal/mol
0.72\ %
1.18 Å

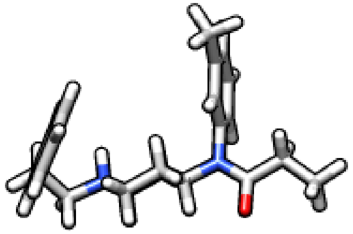

Structure 18
2.53 kcal/mol
0.56 %
1.14 Å

Figure S28. p-Methyl fentanyl

Structure #
ΔG (310.15K)
Boltzmann %
RMSD
ΔG (310.15K/haug)

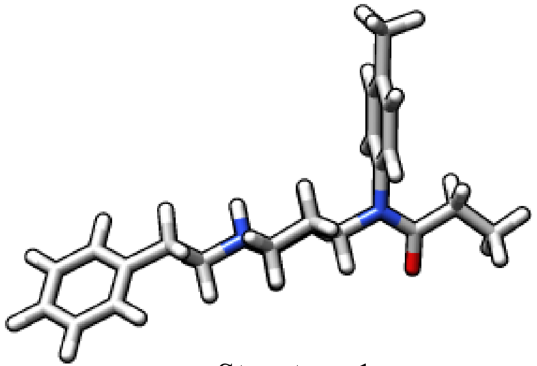

Structure 1
0.00 kcal/mol
18.80 %
0.00 Å
0.00 kcal/mol

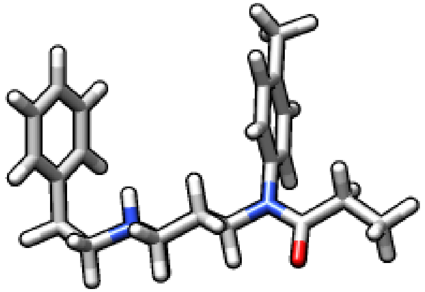

Structure 2
0.16 kcal/mol
14.50 %
1.66 Å
0.31 kcal/mol

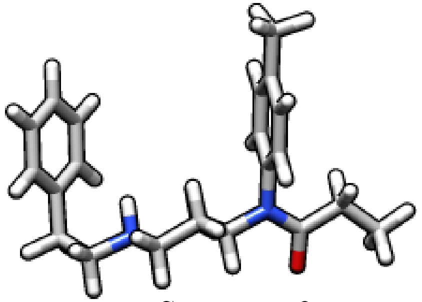

Structure 3
0.23 kcal/mol
12.96 %
1.83 Å
0.55 kcal/mol

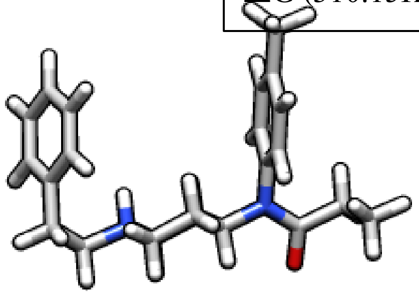

Structure 4
0.28 kcal/mol
11.94 %
1.70 Å
0.39 kcal/mol

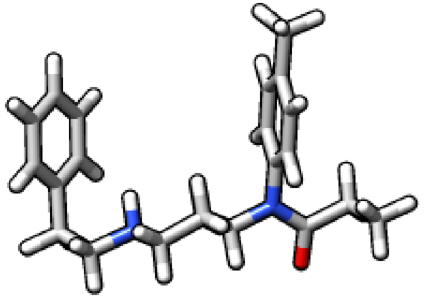

Structure 5
0.39 kcal/mol
9.96 %
1.79 Å
0.56 kcal/mol

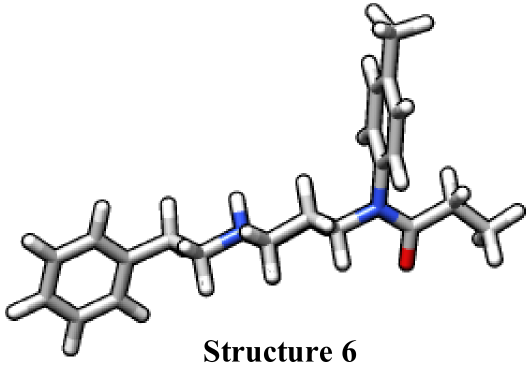

Structure 6
0.51 kcal/mol
8.26 %
0.88 Å
0.70 kcal/mol

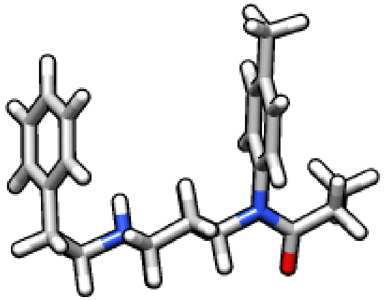

Structure 7
0.51 kcal/mol
8.25 %
1.76 Å
0.85 kcal/mol

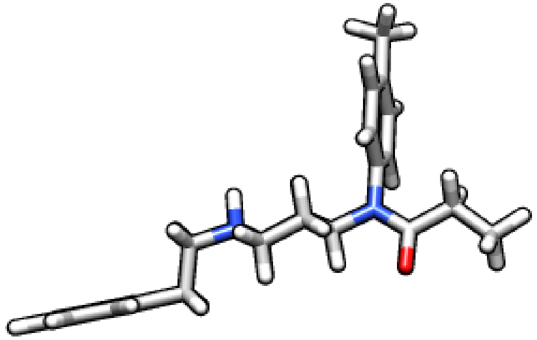

Structure 8
1.14 kcal/mol
2.97 %
0.75 Å

Figure S28. p-Methyl fentanyl

Structure #
ΔG (310.15K)
Boltzmann %
RMSD
ΔG (310.15K/haug)

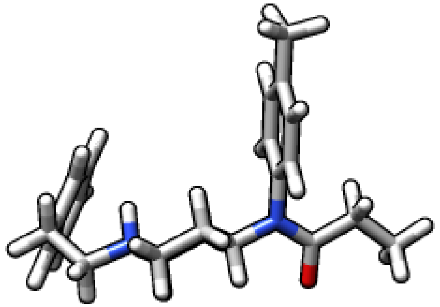

Structure 9
1.38 kcal/mol
2.02 %
1.68 Å

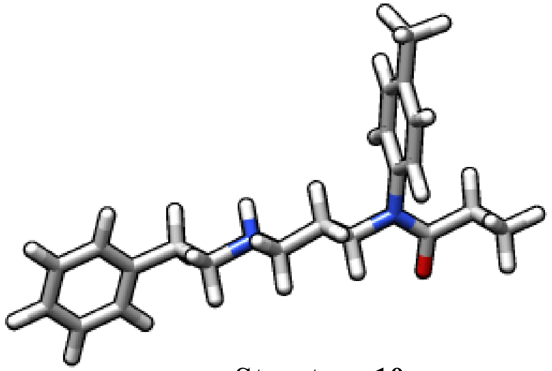

Structure 10
1.40 kcal/mol
1.94 %
0.91 Å

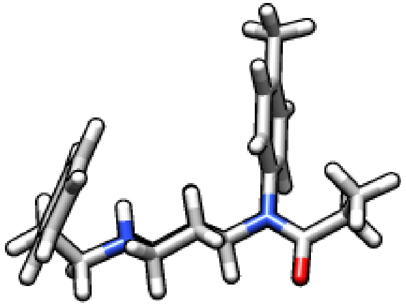

Structure 11
1.74 kcal/mol
1.11 %
1.82 Å

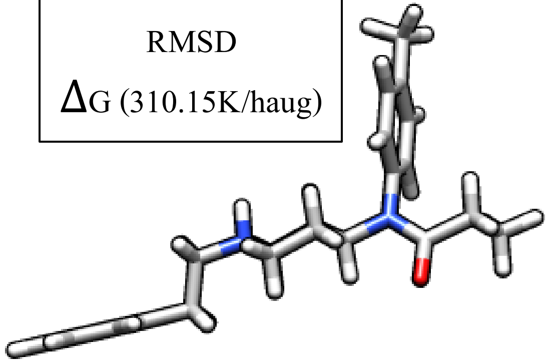

Structure 12
1.87 kcal/mol
0.91 %
0.83 Å

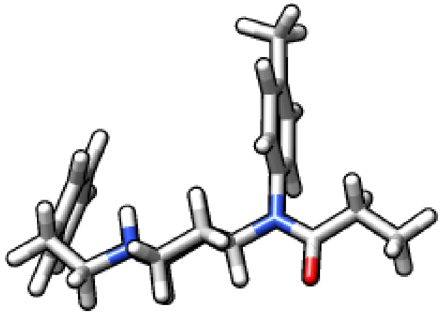

Structure 13
1.90 kcal/mol
0.87 %
1.70 Å

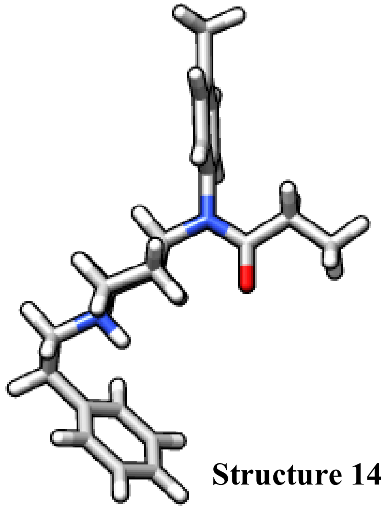

Structure 14
2.08 kcal/mol
0.65 %
1.92 Å

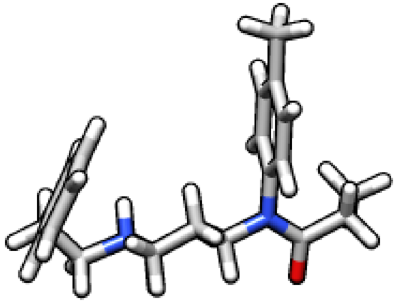

Structure 15
2.10 kcal/mol
0.63 %
1.71 Å

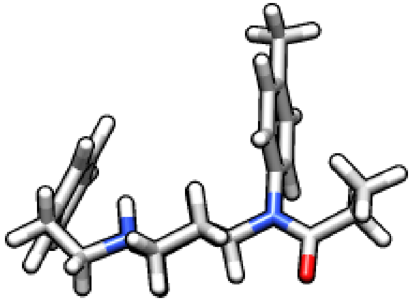

Structure 16
2.11 kcal/mol
0.61 %
1.73 Å

Figure S29. p-Chloro fentanyl

Structure #
ΔG (310.15K)
Boltzmann %
RMSD
ΔG (310.15K/haug)

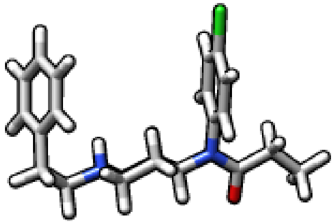

Structure 1
0.00 kcal/mol
21.44 %
0.00 Å
0.00 kcal/mol

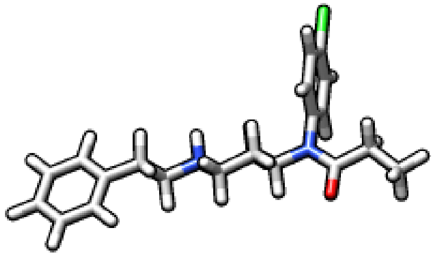

Structure 2
0.17 kcal/mol
16.37 %
1.86 Å
0.17 kcal/mol

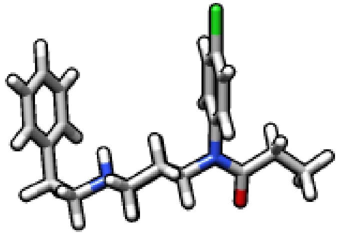

Structure 3
0.29 kcal/mol
13.30 %
0.79 Å
0.28 kcal/mol

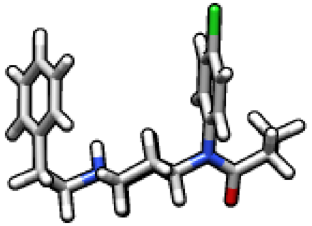

Structure 4
0.71 kcal/mol
6.75 %
0.41 Å
0.70 kcal/mol

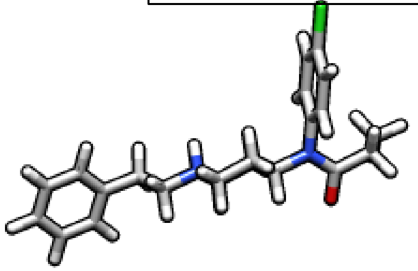

Structure 5
0.83 kcal/mol
5.61 %
1.85 Å
0.85 kcal/mol

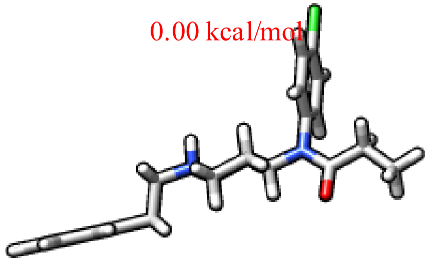

Structure 6
0.86 kcal/mol
5.36 %
2.06 Å
0.86 kcal/mol

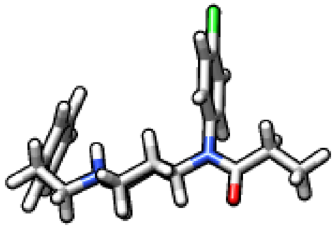

Structure 7
0.90 kcal/mol
4.95 %
0.77 Å

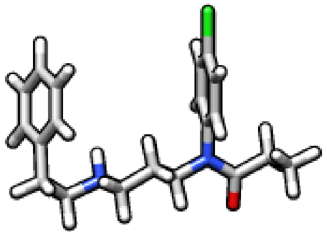

Structure 8
0.92 kcal/mol
4.85 %
0.45 Å

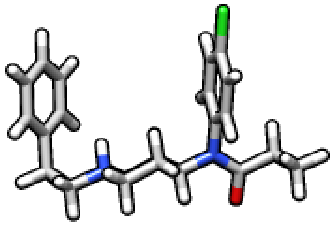

Structure 9
0.97 kcal/mol
4.43 %
0.80 Å

Figure S29. p-Chloro fentanyl

Structure #
ΔG (310.15K)
Boltzmann %
RMSD
ΔG (310.15K/haug)

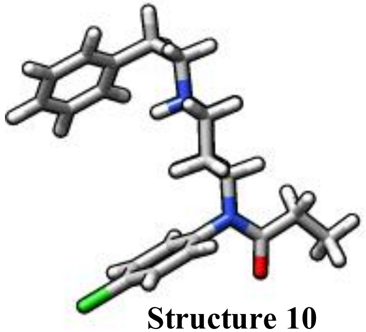

Structure 10
1.16 kcal/mol
3.27 %
1/24 Å

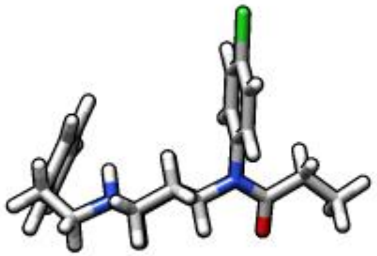

Structure 11
1.17 kcal/mol
3.19 %
1.50 Å

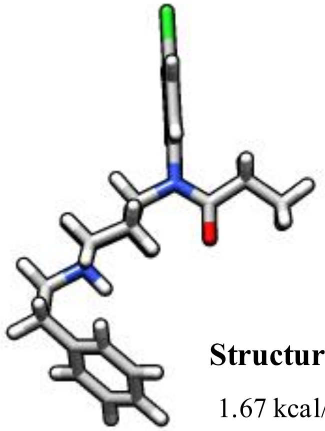

Structure 12
1.67 kcal/mol
1.43 %
2.07 Å

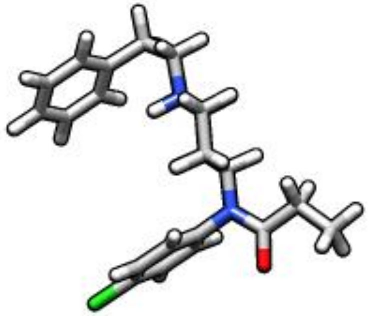

Structure 13
1.69 kcal/mol
1.38 %
0.93 Å

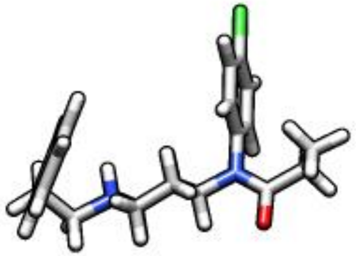

Structure 14
1.84 kcal/mol
1.09 %
1.58 Å

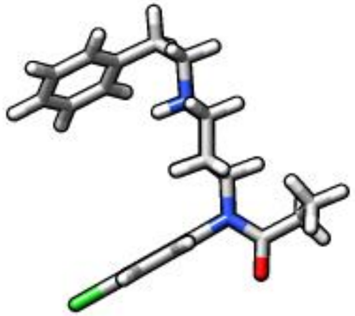

Structure 15
2.04 kcal/mol
0.79 %
1.18 Å

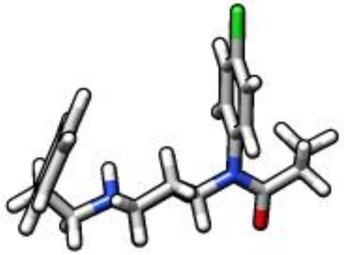

Structure 16
2.05 kcal/mol
0.78 %
0.77 Å

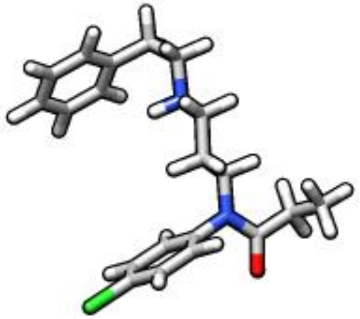

Structure 17
2.10 kcal/mol
0.71 %
1.32 Å

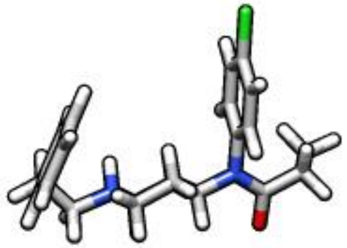

Structure 18
2.22 kcal/mol
0.58 %
0.78 Å

Figure S30. Cis-3-methyl fentanyl

Structure #
ΔG (310.15K)
Boltzmann %
RMSD
ΔG (310.15K/haug)

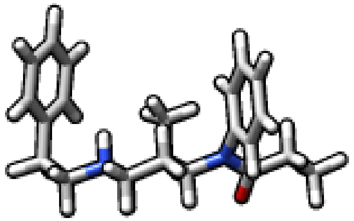

Structure 1
0.00 kcal/mol
34.73%
0.00 Å
0.00 kcal/mol

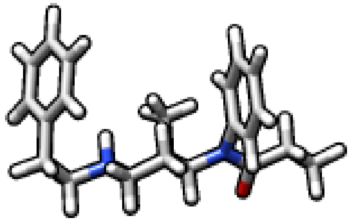

Structure 2
0.29 kcal/mol
21.26 %
0.81 Å
0.46 kcal/mol

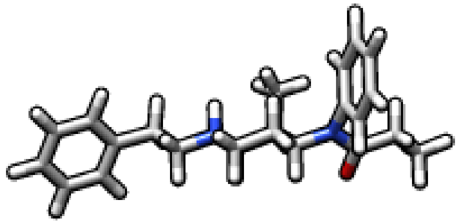

Structure 3
0.61 kcal/mol
12.45 %
1.58 Å
0.40 kcal/mol

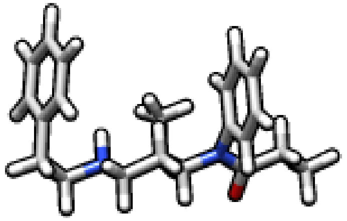

Structure 4
0.95 kcal/mol
7.04 %
0.77 Å
1.30 kcal/mol

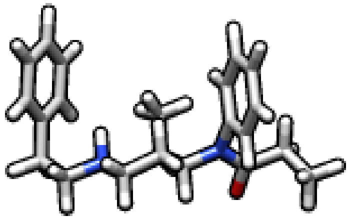

Structure 5
1.06 kcal/mol
5.81 %
0.39 Å
0.58 kcal/mol

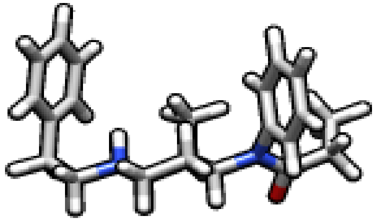

Structure 6
1.46 kcal/mol
2.94 %
0.44 Å

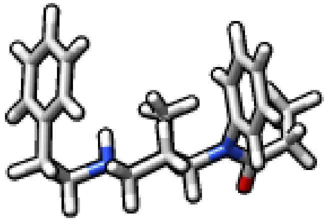

Structure 7
1.47 kcal/mol
2.91 %
0.85 Å

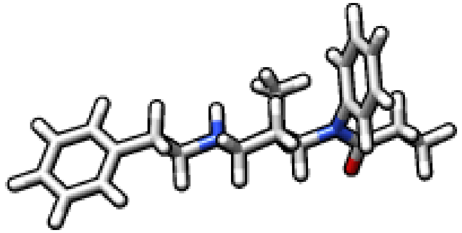

Structure 8
1.53 kcal/mol
2.64 %
1.82 Å

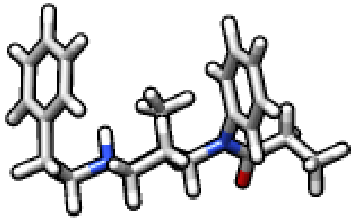

Structure 9
1.74 kcal/mol
1.84 %
0.94 Å

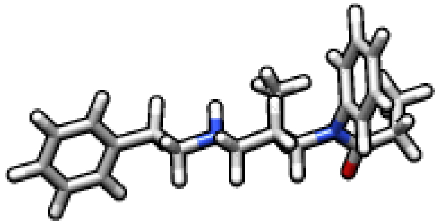

Structure 10
1.96 kcal/mol
1.27 %
1.68 Å

Figure S30. Cis-3-methyl fentanyl

Structure #
ΔG (310.15K)
Boltzmann %
RMSD
ΔG (310.15K/haug)

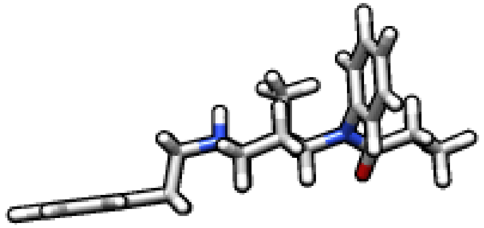

Structure 11
2.16 kcal/mol
0.92 %
1.72 Å

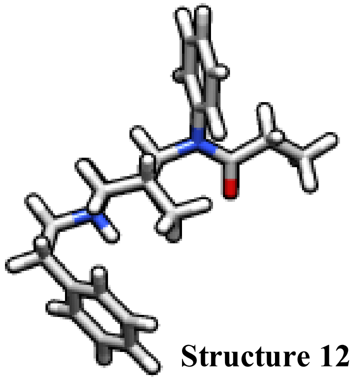

Structure 12
2.45 kcal/mol
0.56 %
1.37 Å

Figure S31. Trans-3-methyl fentanyl

Structure #
ΔG (310.15K)
Boltzmann %
RMSD
ΔG (310.15K/haug)

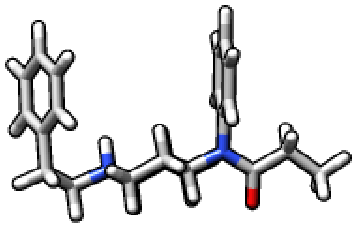

Structure 1
0.00 kcal/mol
34.02 %
0.00 Å
0.00 kcal/mol

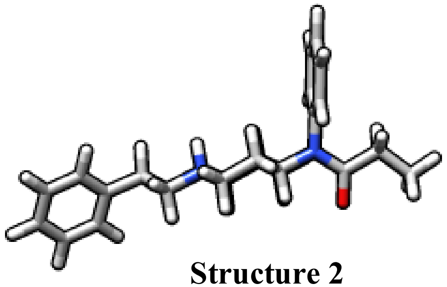

Structure 2
0.22 kcal/mol
23.46 %
1.81 Å
0.12 kcal/mol

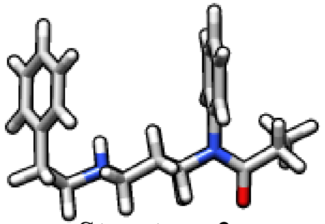

Structure 3
0.93 kcal/mol
7.05 %
0.44 Å
0.70 kcal/mol

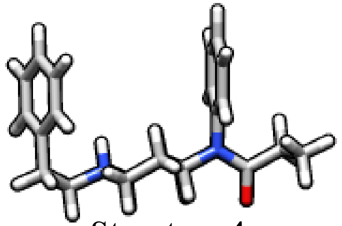

Structure 4
1.26 kcal/mol
4.04 %
0.40 Å

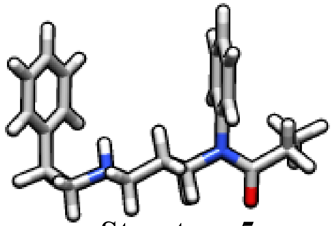

Structure 5
1.28 kcal/mol
3.92 %
0.98 Å

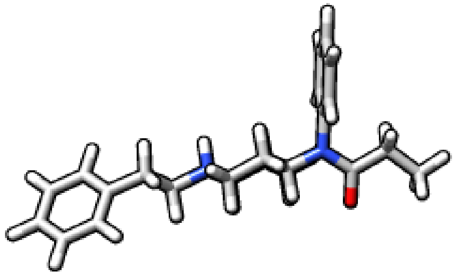

Structure 6
1.36 kcal/mol
3.41 %
1.59 Å

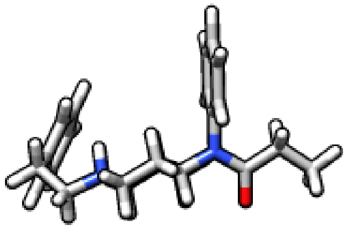

Structure 7
1.51 kcal/mol
2.69 %
1.34 Å

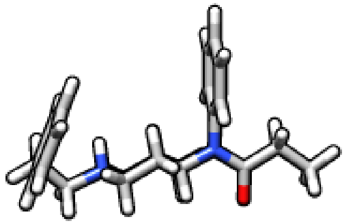

Structure 8
1.54 kcal/mol
2.54 %
1.02 Å

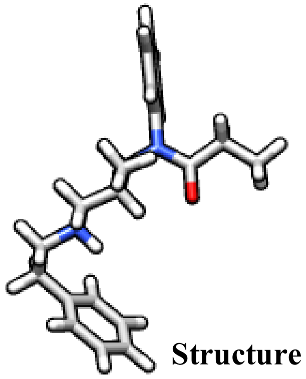

Structure 9
1.69 kcal/mol
1.97 %
1.44 Å

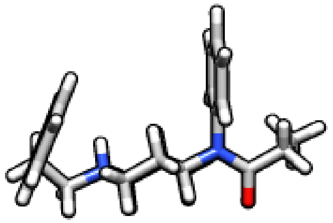

Structure 10
1.79 kcal/mol
1.67 %
1.04 Å

Figure S31. Trans-3-methyl fentanyl

Structure #
ΔG (310.15K)
Boltzmann %
RMSD
ΔG (310.15K/haug)

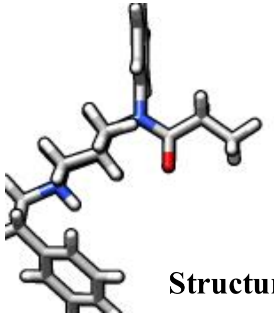

Structure 11
1.81 kcal/mol
1.61 %
1.51 Å

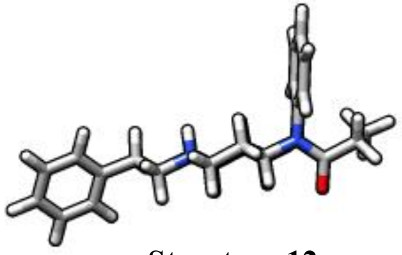

Structure 12
1.09 kcal/mol
2.19 %
1.81 Å

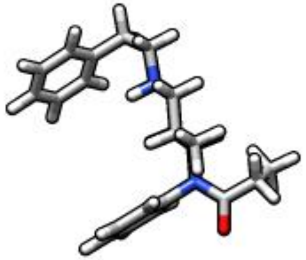

Structure 13
1.92 kcal/mol
1.35 %
1.07 Å

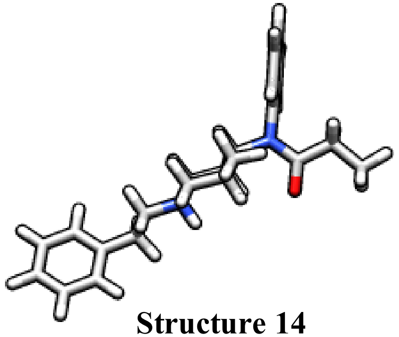

Structure 14
1.94 kcal/mol
1.28 %
2.14 Å

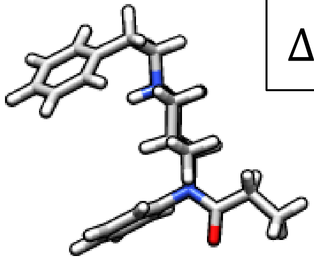

Structure 15
1.95 kcal/mol
1.28 %
1.20 Å

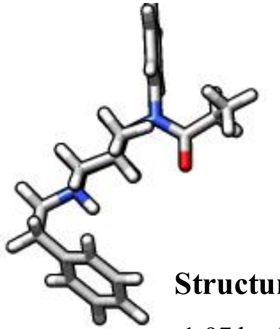

Structure 16
1.97 kcal/mol
1.24 %
1.53 Å

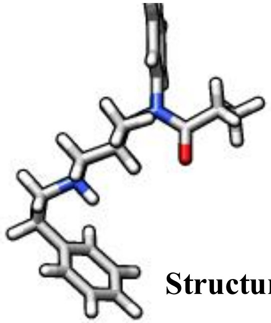

Structure 17
2.09 kcal/mol
0.99 %
1.43 Å

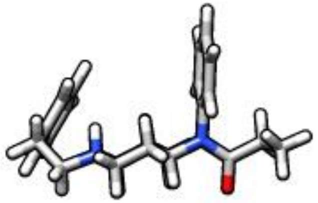

Structure 18
2.12 kcal/mol
0.96 %
1.33 Å

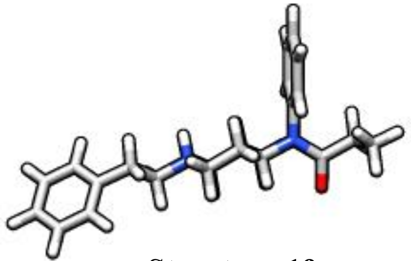

Structure 19
2.12 kcal/mol
0.95 %
1.60 Å

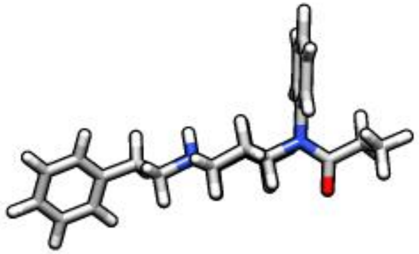

Structure 20
2.19 kcal/mol
0.84 %
1.79 Å

Figure S31. Trans-3-methyl fentanyl

Structure #
ΔG (310.15K)
Boltzmann %
RMSD
ΔG (310.15K/haug)

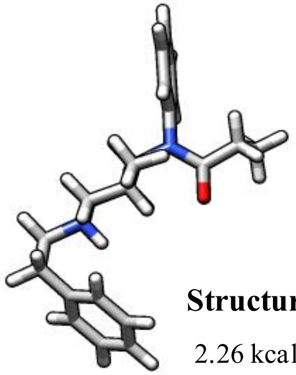

Structure 21
2.26 kcal/mol
0.75 %
1.54 Å

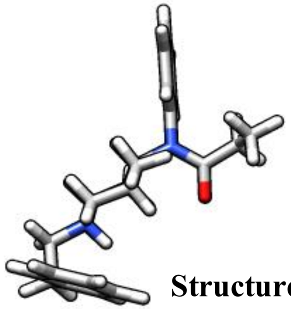

Structure 22
2.31 kcal/mol
0.69 %
1.98 Å

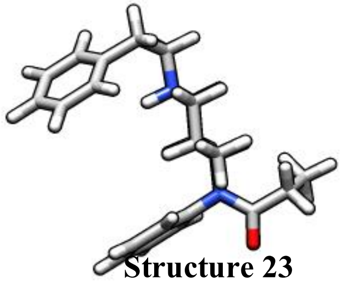

Structure 23
2.36 kcal/mol
0.64 %
1.29 Å

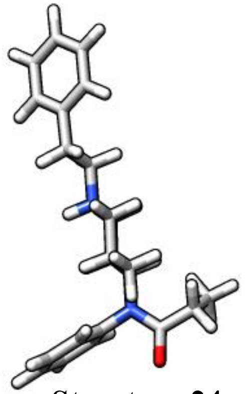

Structure 24
2.36 kcal/mol
0.64 %
2.06 Å

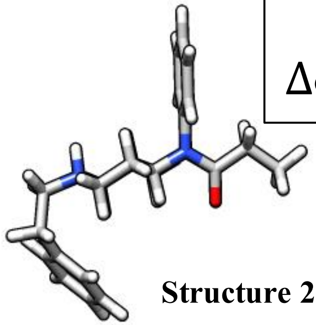

Structure 25
2.41 kcal/mol
0.58 %
1.67 Å

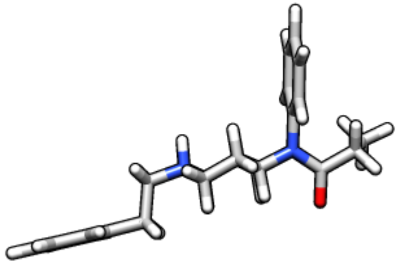

Structure 26
2.49 kcal/mol
0.51 %
1.99 Å

Figure S32. Furanylethyl fentanyl

Structure #

ΔG (310.15K)

Boltzmann %

RMSD

ΔG (310.15K/haug)

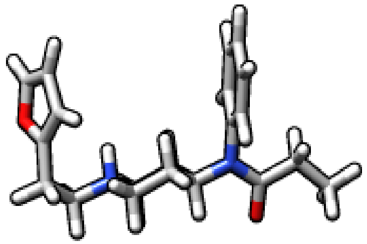

Structure 1

0.00 kcal/mol

26.71 %

0.00 Å

0.00 kcal/mol

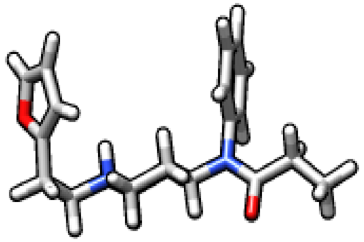

Structure 2

0.05 kcal/mol

24.52 %

0.89 Å

0.08 kcal/mol

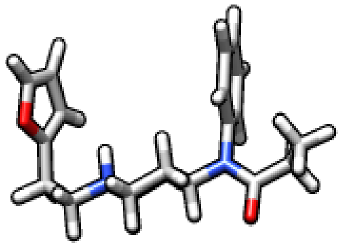

Structure 3

0.50 kcal/mol

11.53 %

0.41 Å

0.41 kcal/mol

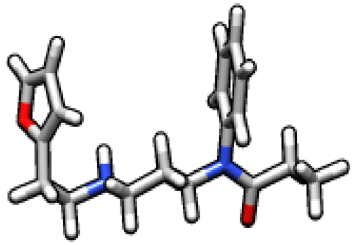

Structure 4

0.75 kcal/mol

7.51 %

0.40 Å

0.81 kcal/mol

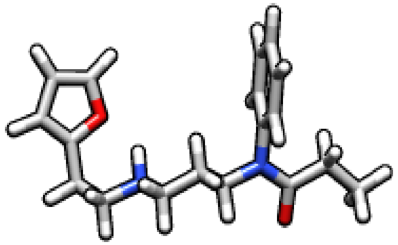

Structure 5

0.77 kcal/mol

7.26 %

0.90 Å

0.75 kcal/mol

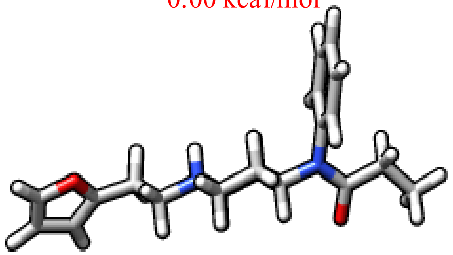

Structure 6

1.04 kcal/mol

4.60 %

1.64 Å

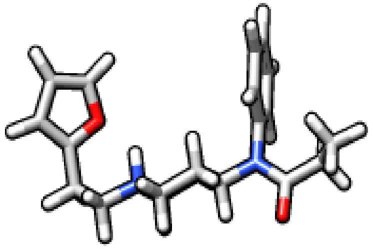

Structure 7

1.34 kcal/mol

2.80 %

0.85 Å

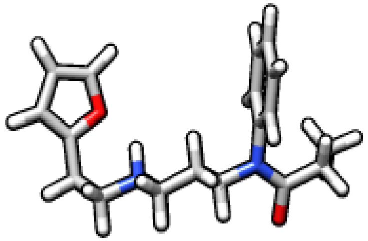

Structure 8

1.47 kcal/mol

2.25 %

1.05 Å

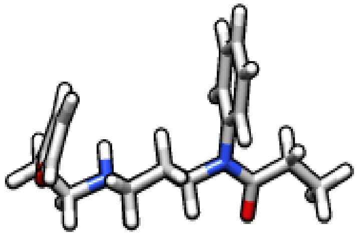

Structure 9

1.50 kcal/mol

2.12 %

0.75 Å

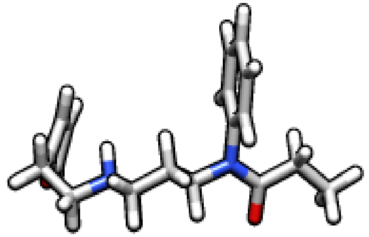

Structure 10

1.75 kcal/mol

1.39 %

1.27 Å

Figure S32. Furanylethyl fentanyl

Structure #
ΔG (310.15K)
Boltzmann %
RMSD
ΔG (310.15K/haug)

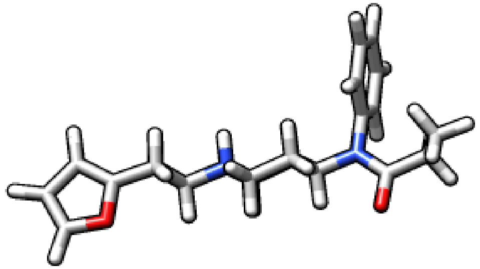

Structure 11
1.76 kcal/mol
1.36 %
1.64 Å

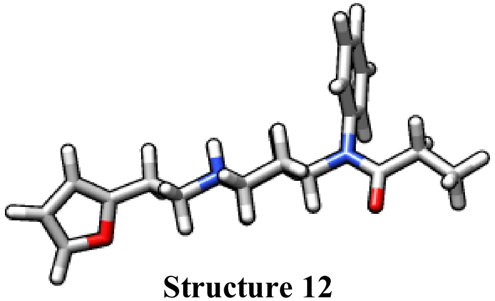

Structure 12
1.90 kcal/mol
1.09 %
1.50 Å

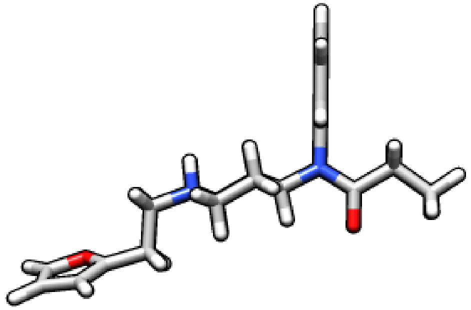

Structure 13
1.92 kcal/mol
1.04 %
1.64 Å

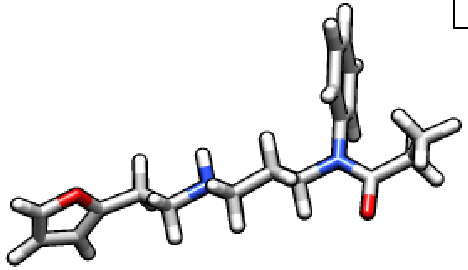

Structure 14
2.08 kcal/mol
0.80 %
1.44 Å

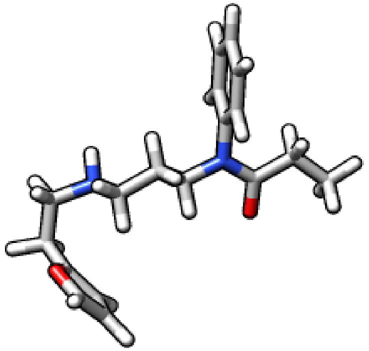

Structure 15
2.26 kcal/mol
0.59 %
1.78 Å

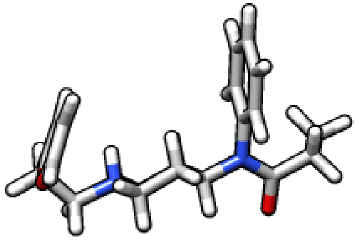

Structure 16
2.27 kcal/mol
0.58 %
0.76 Å

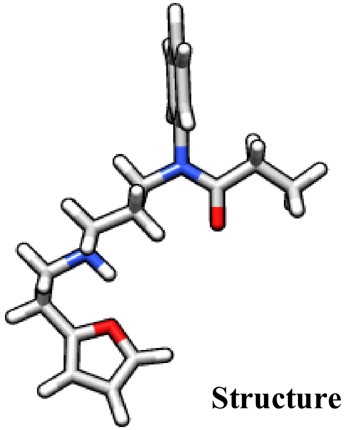

Structure 17
2.35 kcal/mol
0.50 %
1.60 Å

Figure S33. β-hydroxy thiofentanyl

Structure #
ΔG (310.15K)
Boltzmann %
RMSD
ΔG (310.15K/haug)

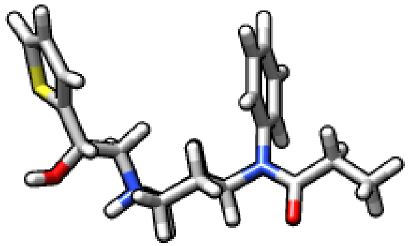

Structure 1
0.00 kcal/mol
21.68 %
0.00 Å
0.00 kcal/mol

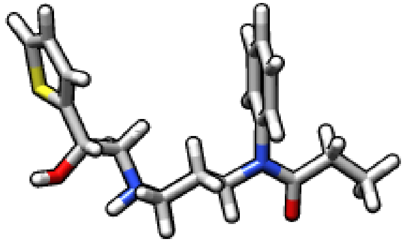

Structure 2
0.07 kcal/mol
19.15 %
0.97 Å
0.19 kcal/mol

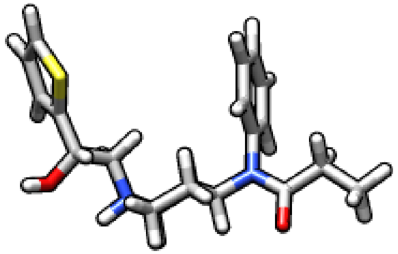

Structure 3
0.21 kcal/mol
15.19 %
0.78 Å
0.25 kcal/mol

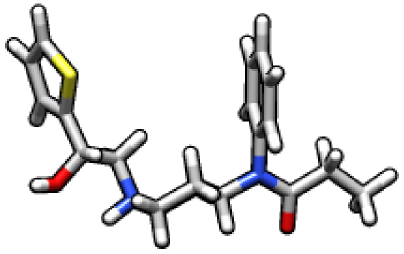

Structure 4
0.35 kcal/mol
12.11 %
0.64 Å
0.49 kcal/mol

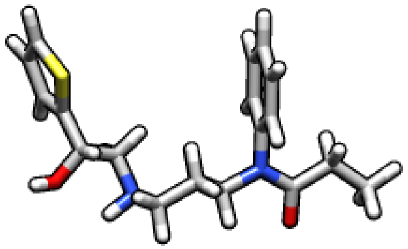

Structure 5
0.83 kcal/mol
5.39 %
0.61 Å
1.01 kcal/mol

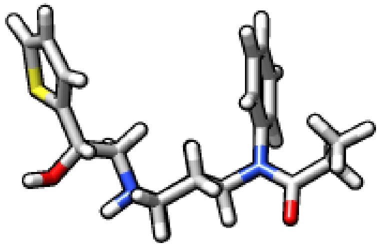

Structure 6
0.98 kcal/mol
4.18 %
0.40 Å

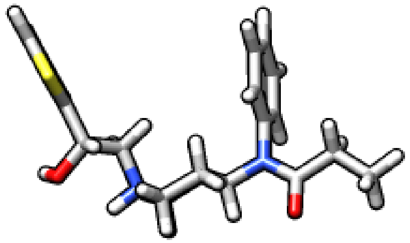

Structure 7
1.24 kcal/mol
2.69 %
0.79 Å

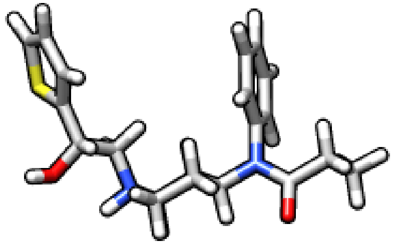

Structure 8
1.33 kcal/mol
2.30 %
0.40 Å

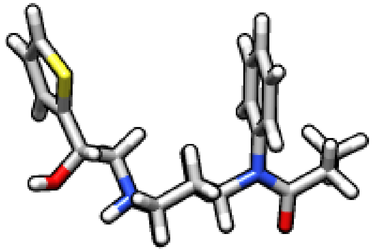

Structure 9
1.59 kcal/mol
1.48 %
0.63 Å

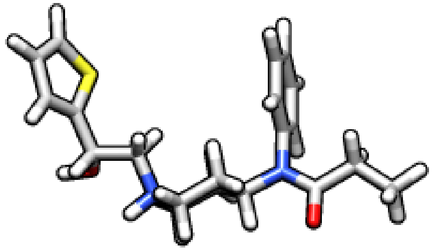

Structure 10
1.65 kcal/mol
1.34 %
0.76 Å

Figure S33. β-hydroxy thiofentanyl

Structure #
ΔG (310.15K)
Boltzmann %
RMSD
ΔG (310.15K/haug)

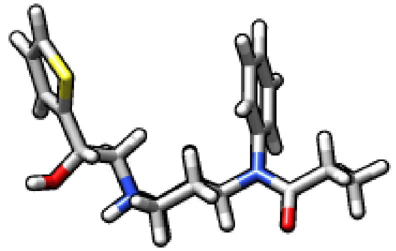

Structure 11
1.76 kcal/mol
1.12 %
0.88 Å

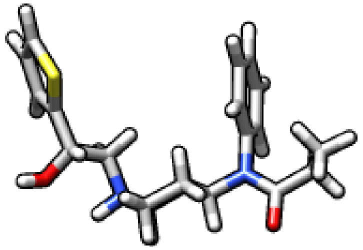

Structure 12
1.78 kcal/mol
1.09 %
0.85 Å

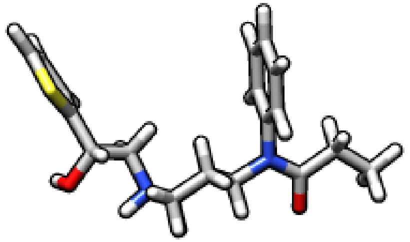

Structure 13
1.81 kcal/mol
1.03 %
0.45 Å

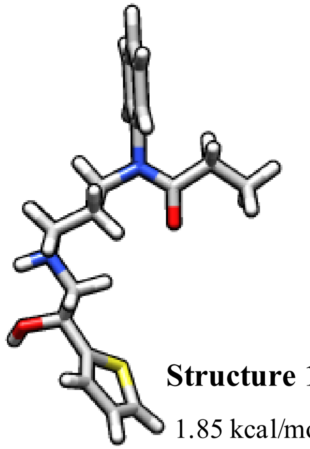

Structure 14
1.85 kcal/mol
0.96 %
1.62 Å

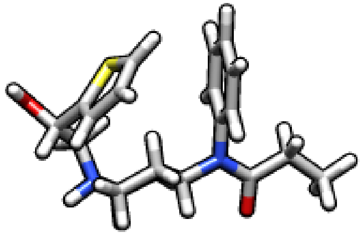

Structure 15
1.91 kcal/mol
0.86 %
1.44 Å

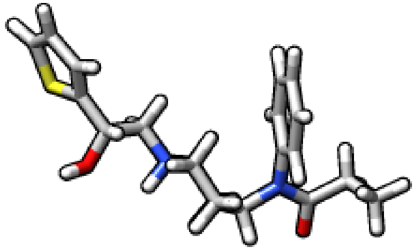

Structure 16
1.96 kcal/mol
0.80 %
1.19 Å

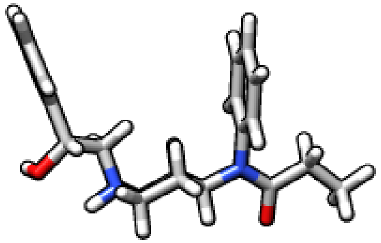

Structure 17
2.05 kcal/mol
0.68 %
0.96 Å

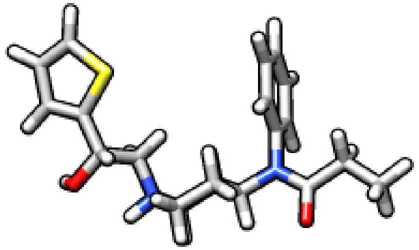

Structure 18
2.14 kcal/mol
0.58 %
0.78 Å

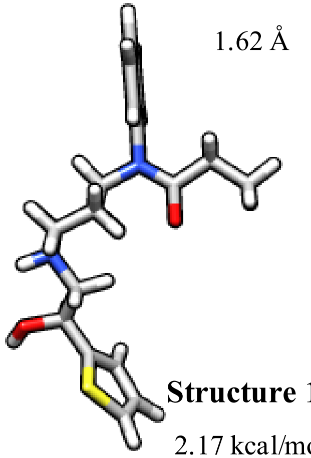

Structure 19
2.17 kcal/mol
0.56 %
1.47 Å

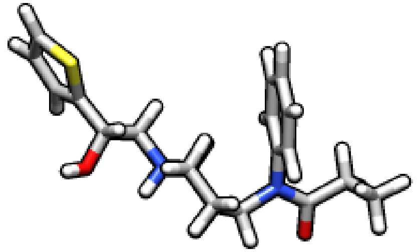

Structure 20
2.22 kcal/mol
0.52 %
1.01 Å

Figure S34. β-Methyl fentanyl

Structure #
ΔG (310.15K)
Boltzmann %
RMSD
ΔG (310.15K/haug)

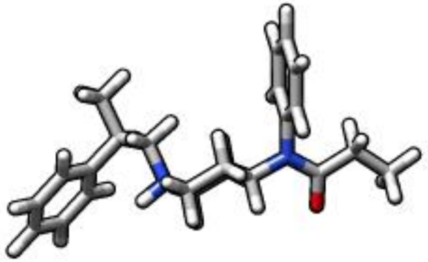

Structure 1
0.00 kcal/mol
30.25 %
0.00 Å
0.00 kcal/mol

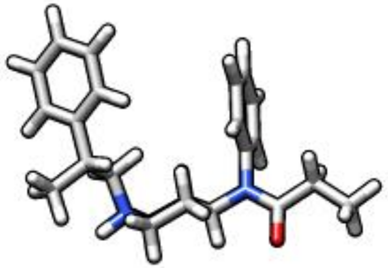

Structure 2
0.61 kcal/mol
10.86 %
1.73 Å
1.15 kcal/mol

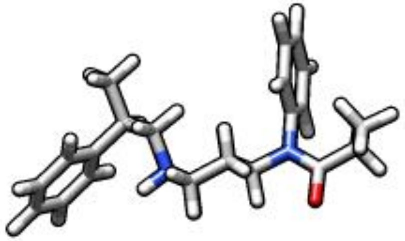

Structure 3
0.78 kcal/mol
8.15 %
0.73 Å
0.90 kcal/mol

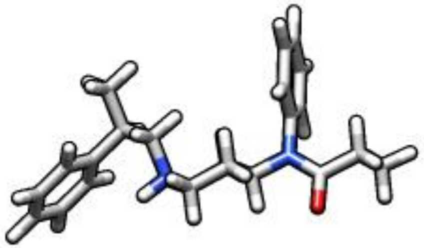

Structure 4
0.86 kcal/mol
7.15 %
0.96 Å
0.85 kcal/mol

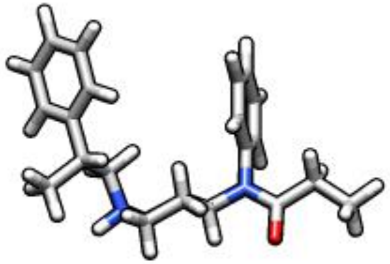

Structure 5
1.14 kcal/mol
4.39 %
1.72 Å
0.45 kcal/mol

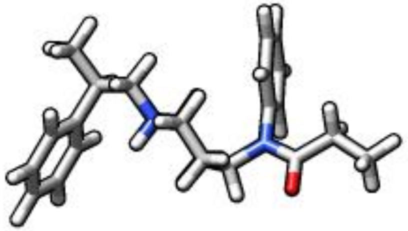

Structure 6
1.16 kcal/mol
4.25 %
0.97 Å

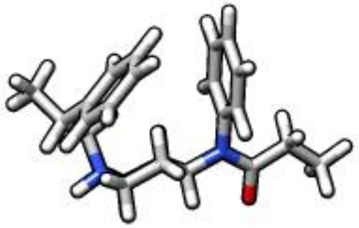

Structure 7
1.22 kcal/mol
3.89 %
2.00 Å

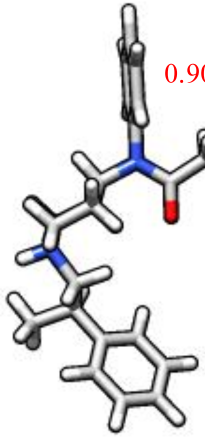

Structure 8
1.26 kcal/mol
3.62 %
1.72 Å

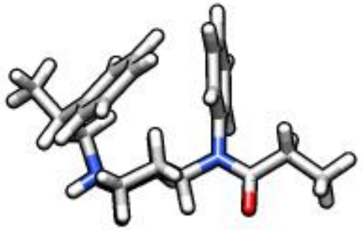

Structure 9
1.41 kcal/mol
2.80 %
1.90 Å

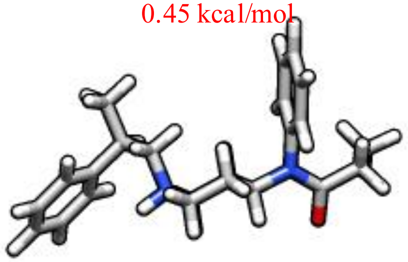

Structure 10
1.47 kcal/mol
2.53 %
0.38 Å

Figure S34. β-Methyl fentanyl

Structure #
ΔG (310.15K)
Boltzmann %
RMSD
ΔG (310.15K/haug)

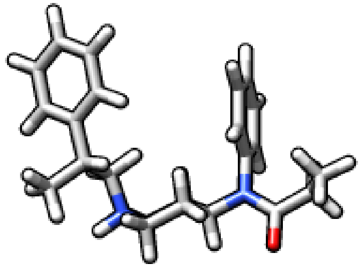

Structure 11
1.75 kcal/mol
1.58 %
1.78 Å

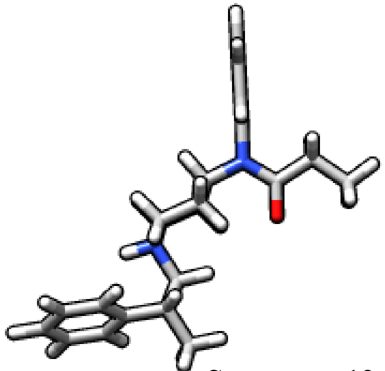

Structure 12
1.82 kcal/mol
1.41 %
1.37 Å

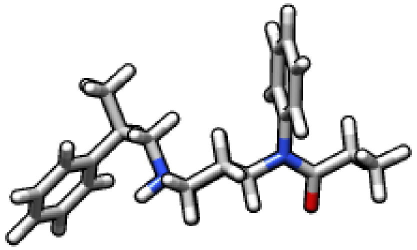

Structure 13
1.82 kcal/mol
1.39 %
0.40 Å

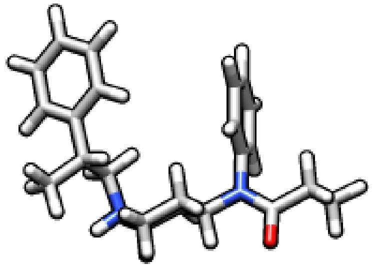

Structure 14
1.83 kcal/mol
1.39 %
1.76 Å

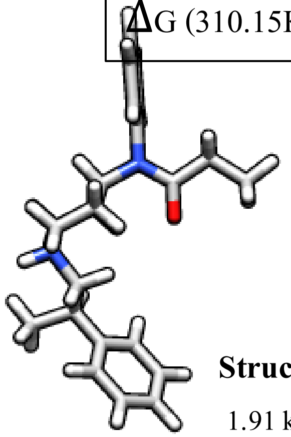

Structure 15
1.91 kcal/mol
1.20 %
1.74 Å

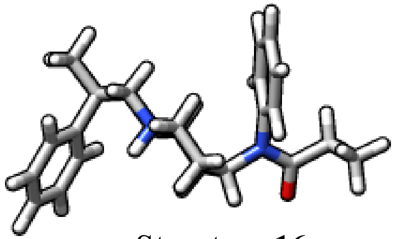

Structure 16
2.04 kcal/mol
0.96 %
0.75 Å

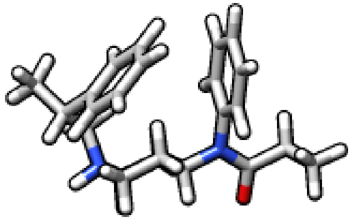

Structure 17
1.95 kcal/mol
1.14 %
2.03

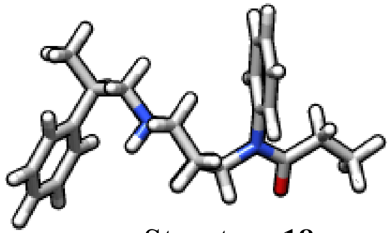

Structure 18
2.08 kcal/mol
0.90 %
0.68 Å

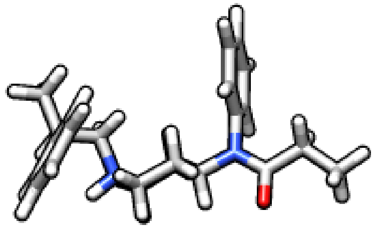

Structure 19
2.11 kcal/mol
0.87 %
1.02 Å

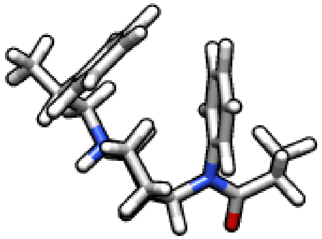

Structure 22
2.08 kcal/mol
0.90 %
1.96 Å

Figure S34. β-Methyl fentanyl

**Structure #**
ΔG (310.15K)
Boltzmann %
RMSD
ΔG (310.15K/haug)

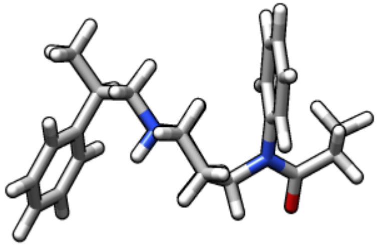

Structure 21

2.08 kcal/mol

0.90 %

0.79 Å

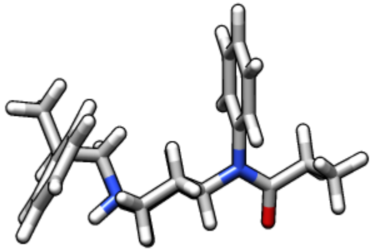

Structure 22

2.12 kcal/mol

0.85 %

1.45 Å

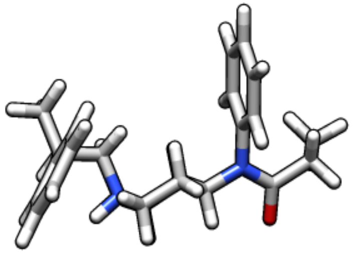

Structure 23

2.30 kcal/mol

0.63 %

1.50 Å

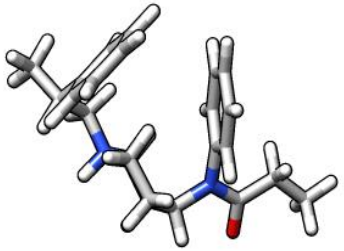

Structure 24

2.36 kcal/mol

0.68 %

1.50 Å

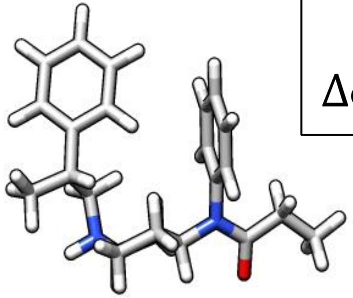

Structure 27

2.37 kcal/mol

0.56 %

2.07 Å

MD Structure, 2 Waters	wB97X-D/SMD	wB97X-D/1 Water+SMD	wB97X-D/2 Waters+SMD
cis 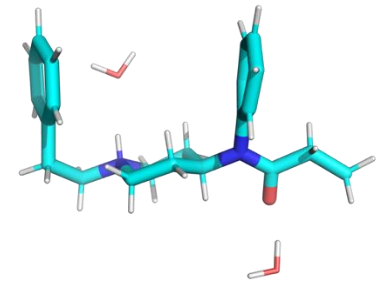	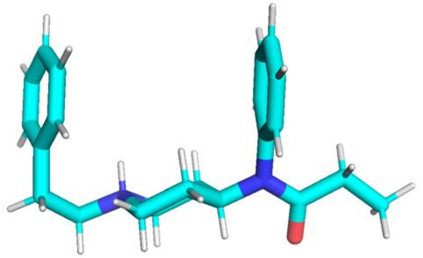	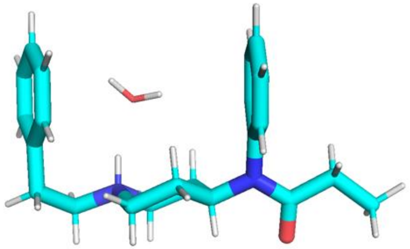	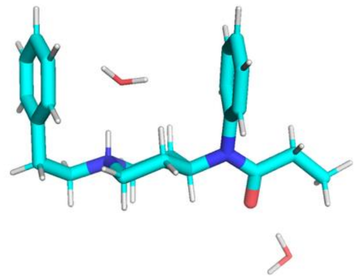
trans A 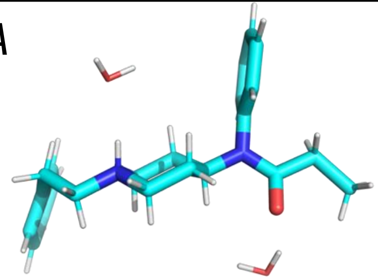	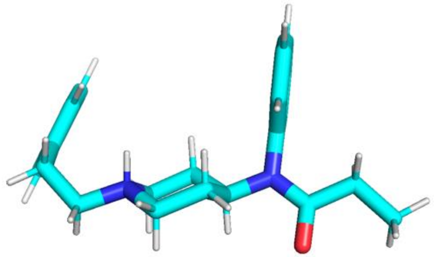	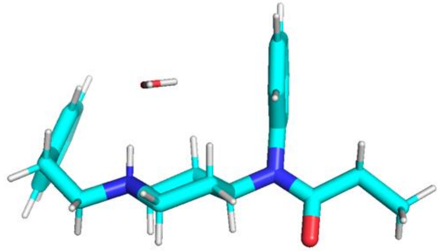	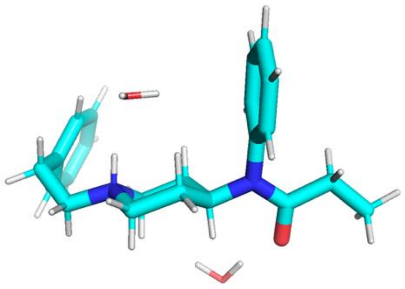
trans B 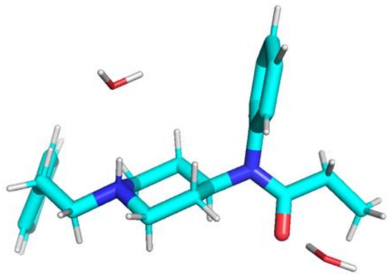	same structure as above	same structure as above	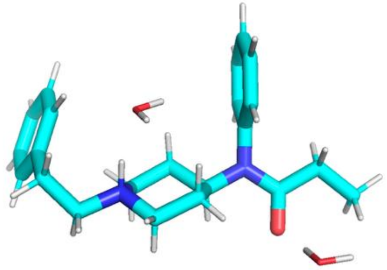
gauche 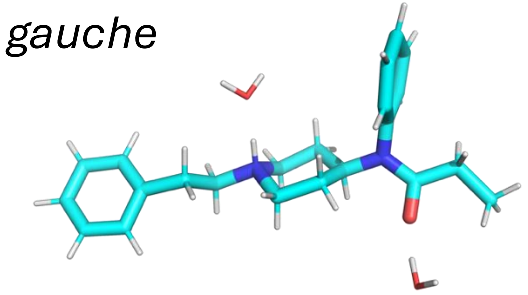	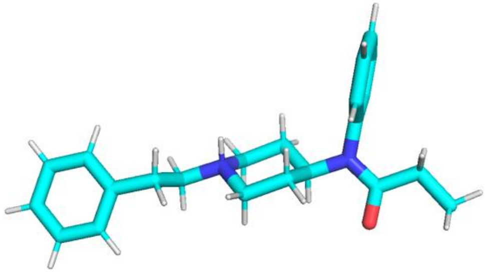	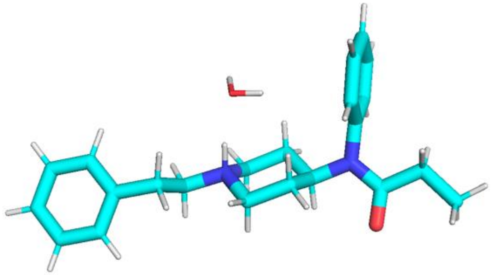	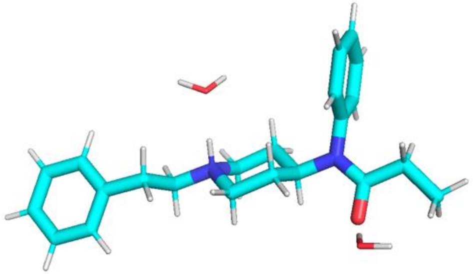

Figure S35. First column- Representative structures from MD of PEPCIT10 in water. Other columns- wB97X-D/6-31++G**/SMD-optimized representative structures when 0, 1, or 2 waters were included explicitly.

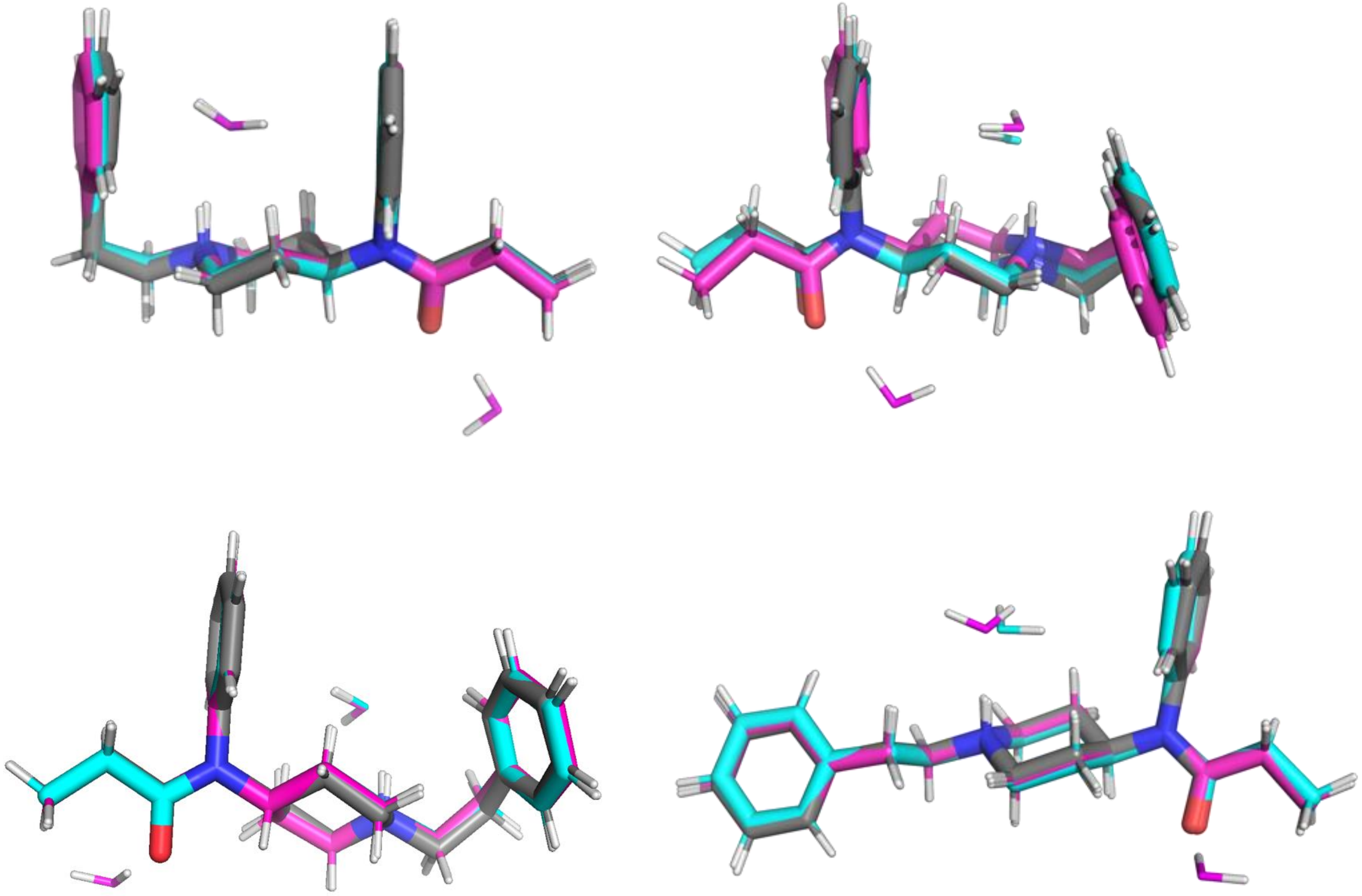

Figure S36. Overlay of representative structures from the PEPCIT10 MD simulation optimized with wB97X-3/6-31++G**/SMD and 0 (gray), 1 (blue), and 2 (pink) explicit waters. Top left: cis; top right: trans A; bottom left: trans B; bottom right: gauche.

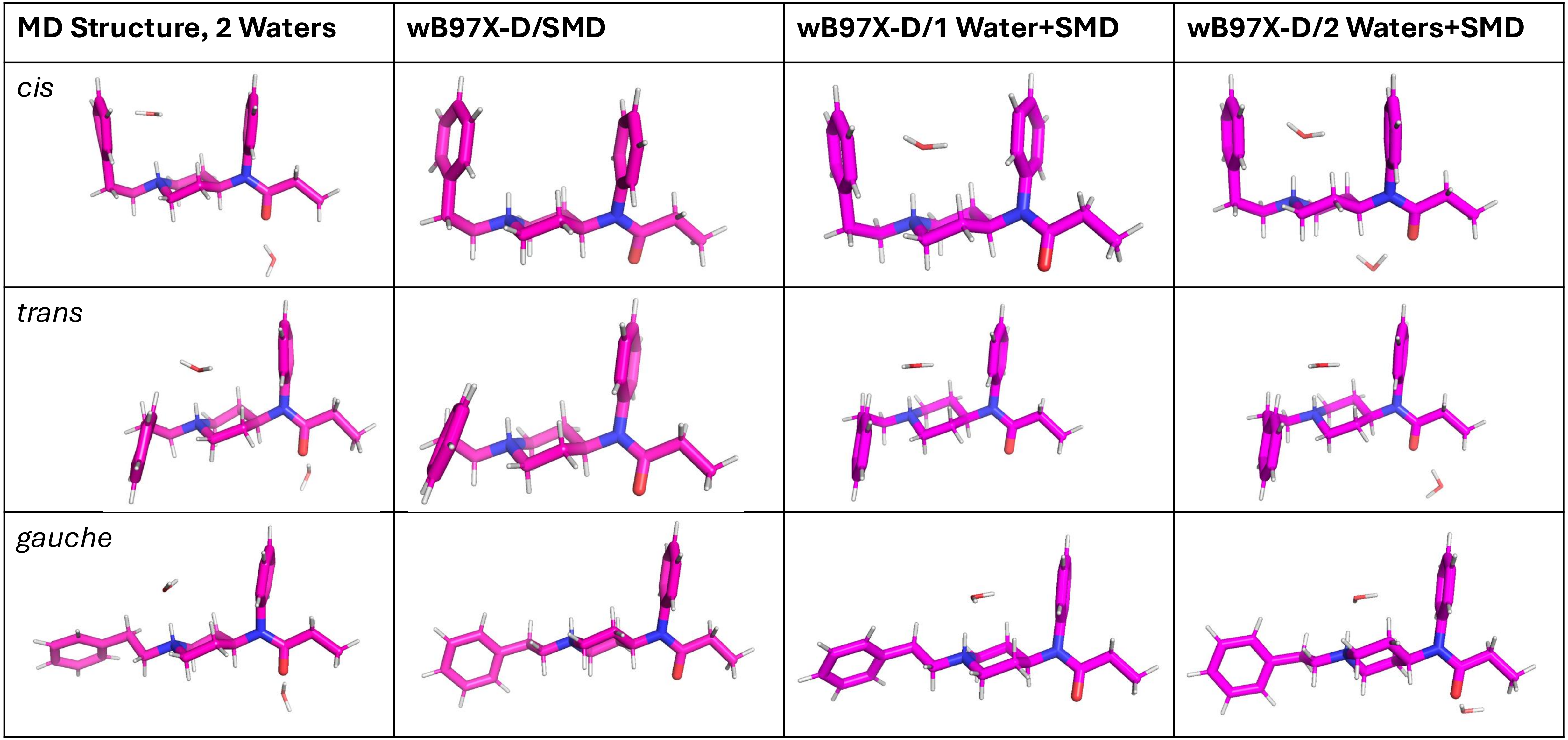

Figure S37. First column- Representative structures from MD of UGIYEP in water. Other columns- wB97X-D/6-31++G**/SMD-optimized representative structures when 0, 1, or 2 waters were included explicitly.

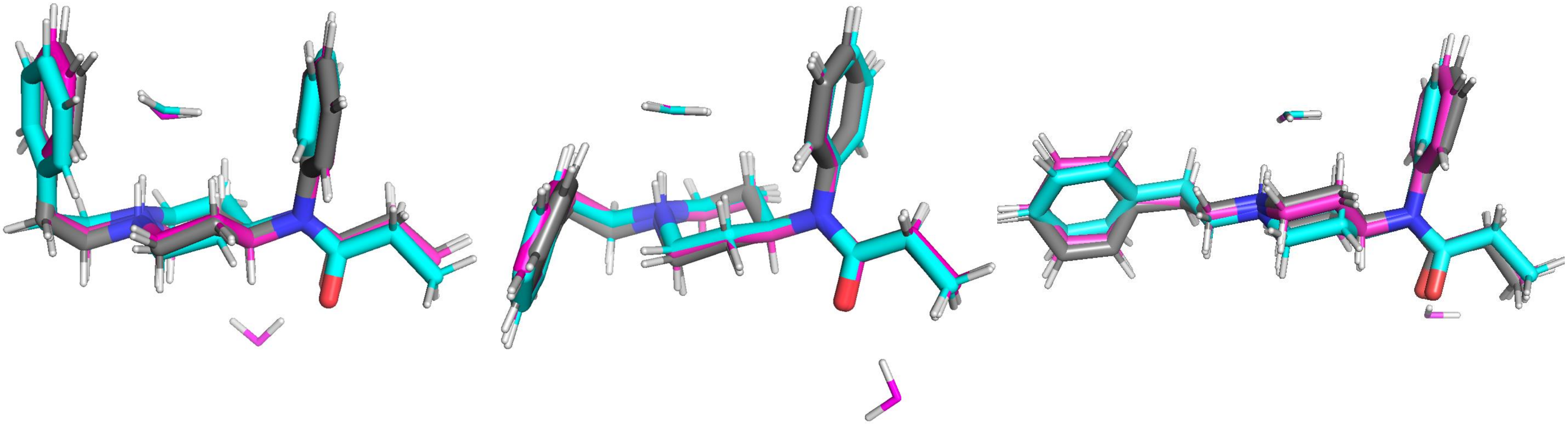

Figure S38. Overlay of representative structures from the UGIYEP MD simulation optimized with wB97X-3/6-31++G**/SMD and 0 (gray), 1 (blue),and 2 (pink) explicit waters. Left: cis; middle: trans; right: gauche.
